# A diagnostic autoantibody signature for primary cutaneous melanoma

**DOI:** 10.18632/oncotarget.25669

**Published:** 2018-07-17

**Authors:** Pauline Zaenker, Johnny Lo, Robert Pearce, Phillip Cantwell, Lester Cowell, Mark Lee, Christopher Quirk, Henry Law, Elin Gray, Mel Ziman

**Affiliations:** ^1^ School of Medical and Health Sciences, Edith Cowan University, Joondalup, Perth, WA, Australia; ^2^ School of Science, Edith Cowan University, Joondalup, Perth, WA, Australia; ^3^ Hollywood Specialist Centre, Hollywood Private Hospital, Nedlands, Perth, WA, Australia; ^4^ Level 1 Melanoma, Fremantle, Perth, WA, Australia; ^5^ St John of God Hospital, Subiaco, Perth, WA, Australia; ^6^ Dermatology Specialist Group, Ardross, Perth, WA, Australia; ^7^ Skin Check WA, Inglewood, Perth, WA, Australia; ^8^ Department of Pathology and Laboratory Medicine, The University of Western Australia, Crawley, Perth, WA, Australia

**Keywords:** melanoma, diagnosis, autoantibody, microarray, biomarker

## Abstract

Melanoma is an aggressive form of skin cancer that is curable by surgical excision in the majority of cases, if detected at an early stage. To improve early stage melanoma detection, the development of a highly sensitive diagnostic test is of utmost importance. Here we aimed to identify antibodies to a panel of tumour associated antigens that can differentiate primary melanoma patients and healthy individuals. A total of 245 sera from primary melanoma patients and healthy volunteers were screened against a high-throughput microarray platform containing 1627 functional proteins. Following rigorous statistical analysis, we identified a combination of 10 autoantibody biomarkers that, as a panel, displays a sensitivity of 79%, specificity of 84% and an AUC of 0.828 for primary melanoma detection. This melanoma autoantibody signature may prove valuable for the development of a diagnostic blood test for routine population screening that, when used in conjunction with current melanoma diagnostic techniques, could improve the early diagnosis of this malignancy and ultimately decrease the mortality rate of patients.

## INTRODUCTION

Worldwide, skin cancer remains a major health concern. The incidence of cutaneous melanoma, the most aggressive and treatment resistant type of skin cancer, continues to increase and New Zealand followed by Australia have the highest incidence rates [1]. Recent data shows that Australians are four times more likely to develop a cancer of the skin than any other type of cancer [2]. Detecting the primary melanoma tumours at an early stage results in a 5-year survival rate as high as 99%, whereas 5-year survival for late stage patients is only 15–20% [3], indicating the importance of the timely diagnosis of this malignancy. A number of diagnostic screening methods are currently available for the detection of melanoma and include the visual examination of suspicious lesions using dermoscopy, reflectance confocal microscopy, total body photography, teledermatology and mobile phone applications. However, all of these have limitations, including their high subject to observer bias. Moreover, it is questionable whether these methods are suitable for the screening of people at risk of melanoma development, such as patients with a substantial number of moles on their body (>100), those with a family history, cases of occult melanoma, or those with very thin and unpigmented primary lesions [4].

A routine blood test used as an adjunct to currently utilised diagnostic approaches may enable improved melanoma screening diagnostic efficiency, especially in cases for which current diagnostic techniques are suboptimal. To date, many blood based biomarkers have been proposed for melanoma prognosis, indication of recurrence, and assessment of treatment response, including microRNAs [5], circulating tumour cells [6] and circulating tumour DNA [7]. However, none of these appear to be sufficiently sensitive to detect the initial transformation to malignancy and may therefore not be suitable diagnostic biomarkers of early stage melanoma.

Autoantibodies (AAbs) may provide a more advantageous blood based biomarker, as they reflect the initial humoral immune response against a tumour and their increased levels can be detectable months to years prior to clinical evidence of a primary tumour [8]. While the mechanisms involved in the production of AAbs in cancer patients (reviewed recently in Zaenker *et al.* 2016 [9]), remain speculative, AAbs are well known to be sensitive biomarkers in the detection and surveillance of many types of tumours [10–14]. Their diagnostic utility in melanoma, however, is yet to be conclusively demonstrated.

**Figure 1:**
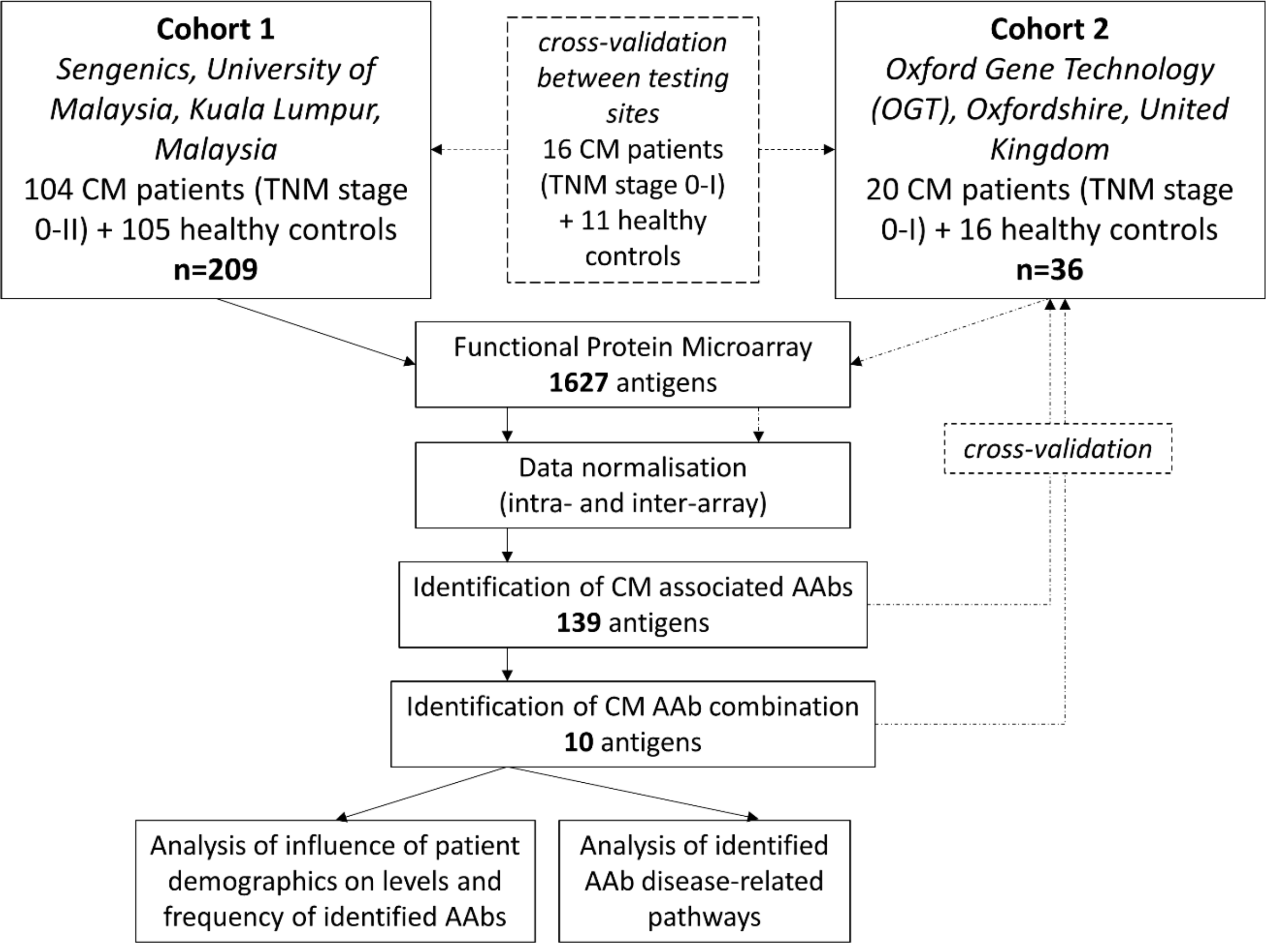
Study design flowchart

High-density protein microarrays allow the functional testing of thousands of proteins simultaneously, increasing the chance of discovery of new autoantibody signatures [15]. These microarrays, in which proteins are immobilised in their natural conformations, enable the identification of AAb profiles within patient sera [16]. Here we utilised the Immunome^™^ Protein Array containing 1627 proteins, developed by Oxford Gene Technology, Oxfordshire, United Kingdom [17], to screen sera from a total of 124 early stage melanoma patients and 121 healthy volunteers. We utilised a novel approach to the statistical analysis of protein microarray data, in order to identify the most predictive panel of AAbs for melanoma diagnosis. First, we identified individual autoantibody biomarkers that were most commonly detectable in the patient cohort. Then, a random forest and classical classification tree analysis [18, 19] was performed to identify a panel of 10 autoantibody biomarkers that in combination significantly differentiated primary melanoma patient sera from healthy control sera. We further investigated whether patient and tumour characteristics affected the breadth and magnitude of the serologic autoantibodies. Finally, we explored AAb biomarker associated disease related pathways using the STRING functional protein association network (Figure 1).

## RESULTS

### Study cohort

The 245 samples used in the study comprised a total of 124 early stage melanoma patients (TNM stages *in situ*, I or II) and 121 healthy controls. The participant characteristics are summarised in Table 1. The number of males was higher than the number of females in both cohorts and in cohort 1, patients were slightly older than healthy volunteers (mean and standard deviation of 62.5 ± 16.3 versus 56.5 ± 12.9 years, *p* = 0.003). This was largely due to the difference in age of the male participants between patients and healthy volunteers (63.7 **±** 14.7 versus 56.8 **±** 13.2 years, *p* = 0.004). There was no significant difference in the mean age of female patients relative to female controls in cohort 1 (59.8 ± 19.4 versus 55.7 ± 12.3 years, *p* = 0.309).

**Table 1:** Clinicopathological characteristics of the study participants

	Cohort 1		Cohort 2	
Group	Early-stage CM patients	Healthy volunteers	Early-stage CM patients	Healthy volunteers
total cohort number	209		36	
sample number	104	105	20	16
Female, *n* (%)	32 (30.8)	35 (33.3)	5 (25.0)	3 (18.8)
Male, *n* (%)	72 (69.2)	70 (66.7)	15 (75.0)	13 (81.2)
Mean age ± SD (years)	62.5 ± 16.3	56.5 ± 12.9	57.2 ± 13.5	55.8 ± 13.4
Age range (years)	20–96	20–83	26–76	25–80
*TNM stage, n (%)*				
0 (*in situ*)	45 (43.3)		15 (75.0)	
I	39 (37.5)		5 (25.0)	
II	20 (19.2)		0 (0.0)	
*Site of primary tumour, n (%)*				
Head and Neck	16 (15.4)		4 (20.0)	
Trunk	41 (39.4)		8 (40.0)	
Extremities	44 (42.3)		8 (40.0)	
Multiple primary melanoma with multiple tumour sites	3 (2.9)		0 (0.0)	
*Melanoma subtype, n (%)*				
SSM	19 (18.3)		4 (20.0)	
NM	10 (9.6)		0 (0.0)	
LMM	8 (7.7)		0 (0.0)	
ALM	0 (0.0)		0 (0.0)	
unclassified	26 (25.0)		9 (45.0)	
not reported	41 (39.4)		7 (35.0)	
*Breslow thickness, n (%)*				
≤1 mm	77 (74.0)		20 (100.0)	
1-2 mm	11 (10.6)		0 (0.0)	
2-4 mm	9 (8.7)		0 (0.0)	
4+ mm	7 (6.7)		0 (0.0)	
*Clark level, n (%)*				
1	44 (42.3)		14 (70.0)	
2	21 (20.2)		3 (15.0)	
3	12 (11.5)		2 (10.0)	
4	26 (25.0)		1 (5.0)	
5	1 (1.0)		0 (0.0)	
*Ulceration, n (%)*				
present	22 (21.2)		3 (15.0)	
absent	65 (62.5)		14 (70.0)	
not reported	17 (16.3)		3 (15.0)	
*Mitotic rate, n (%)*				
≤1 mm^2^	69 (66.3)		16 (80.0)	
1-5 mm^2^	12 (11.5)		0 (0.0)	
5-10 mm^2^	6 (5.8)		0 (0.0)	
10+ mm^2^	4 (3.9)		0 (0.0)	
not reported	13 (12.5)		4 (20.0)	
*Regression, n (%)*				
present	39 (37.5)		8 (40.0)	
absent	28 (26.9)		4 (20.0)	
not reported	37 (35.6)		8 (40.0)	
*History of multiple CM, n (%)*				
Yes	26 (25.0)		5 (25.0)	
No	77 (74.0)		14 (70.0)	
not reported	1 (1.0)		1 (5.0)	
*History of NMSC, n (%)*				
Yes	33 (31.7)		6 (30.0)	
No	67 (64.4)		9 (45.0)	
not reported	4 (3.9)		5 (25.0)	
*History of other cancer, n (%)*				
Yes	10 (9.6)		1 (5.0)	
No	87 (83.7)		14 (70.0)	
not reported	7 (6.7)		5 (25.0)	

Numbers are rounded to 1 decimal; SD, standard deviation; CM, cutaneous melanoma; NMSC, non-melanoma skin cancer; SSM, superficial spreading melanoma; NM, nodular melanoma; LMM, lentigo maligna melanoma; ALM, acral lentiginous melanoma

### Melanoma-associated AAb biomarkers

In cohort 1, a total of 748 antigens were identified to preferentially react with the patient sera as indicated by their positive biomarker scores. Of those, 139 resulted in biomarker scores of greater than 5 and were therefore considered to have a potential diagnostic value for melanoma (Supplementary Table 1.2). The majority of the identified 139 antigens displayed very high specificity, ranging from 86.7%–100% (mean of 97%), while their sensitivity as single biomarkers ranged from 2.9% to 18.3% (mean of 9.9%), which is comparable to similar cancer AAb studies [20, 21]. Notably, 20/139 (14.4%) antigens did not react with any of the healthy control samples.

**Figure 2:**
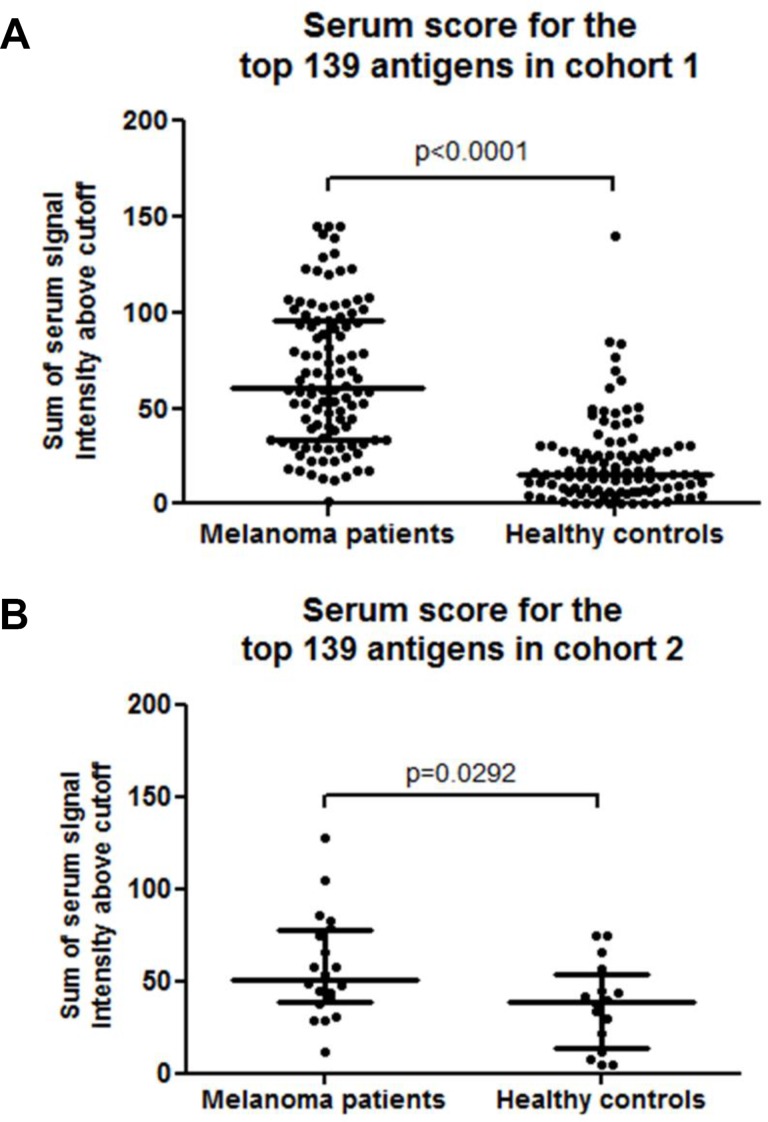
(**A**) Dot plot of melanoma patient and healthy control serum scores for the top 139 individual melanoma-associated biomarkers in cohort 1, the horizontal lines represent the median and IQR of all serum scores in each cohort, dots represent individual samples. (**B**) Dot plot of melanoma patient and healthy control serum scores for the top 139 individual melanoma-associated biomarkers in cohort 2.

The serum scores of melanoma patients (median 60.5; IQR 33.9–95.9) were significantly higher than those for healthy controls (median 15.5; IQR 6.7–27.7) (*p* < 0.001, Figure 2A). To evaluate the diagnostic performance of the identified biomarkers in a different cohort of samples, we calculated the serum scores for the top 139 biomarkers using the 36 samples included in cohort 2. Patient serum scores were again significantly higher, with a median of 51.1 (IQR 38.7–77.7) compared to healthy control median serum score of 38.9 (IQR 14.1–53.7, *p* = 0.029, Figure 2B), supporting the validity of the top 139 biomarkers.

Most of the identified markers are novel and are not known for their association with melanoma. It is however important to note that many were reactive against transcription factors, tumour suppressors and promoters, markers of apoptosis, and regulators of pigmentation and T-cell differentiation that may influence an array of cancer-related pathways. Some of the top 139 seroreactive antigens such as VEGFb, p53, MITF, KIT and MLANA [22] have previously been associated with melanoma and cancer in general, indicating that the detected autoantibody response may be derived from an antitumour response.

The generated STRING protein association network of the top 139 antigens can be viewed in the Supplementary Figure 1 and a detailed table containing a short protein description and interaction scores is summarised in Supplementary Table 1.3 and Supplementary Table 2.5. Supplementary Table 2.1, 2.2, 2.3 and 2.4 detail the biological functions, molecular pathways, cellular location and KEGG pathways associated with the submitted list of proteins. Interestingly, most of the seroreactive proteins are intracellular antigens (101/139), of which the majority are contained within the nucleus (88/139), a cellular location that is usually protected from immune surveillance. Many cancer autoantibody studies have also reported detection of AAbs against nuclear antigens and this has been suggested to be due to spillage of the intracellular contents into the surrounding tissue following cell death in cancer [9]. Nuclear antigens generally do not undergo cross-presentation in the negative selection process of B-cell maturation and may therefore result in enhanced immune reactions. Furthermore, the top 139 identified biomarkers appear primarily related to general cancer pathways, including apoptosis, pathways associated with the immune response and cell cycle, p53 signalling and the MAPK signalling pathway, the main pathway associated with melanomagenesis, highlighting the biological relevance of the identified biomarkers.

### AAb biomarker combination

Since the development of a diagnostic blood test that is comprised of 139 biomarkers is impractical and hence not clinically applicable, we utilised a two stage analysis approach involving random forest and classification tree analysis [18, 19] to identify a combination of 10 biomarkers or less with the highest diagnostic potential.

**Figure 3:**
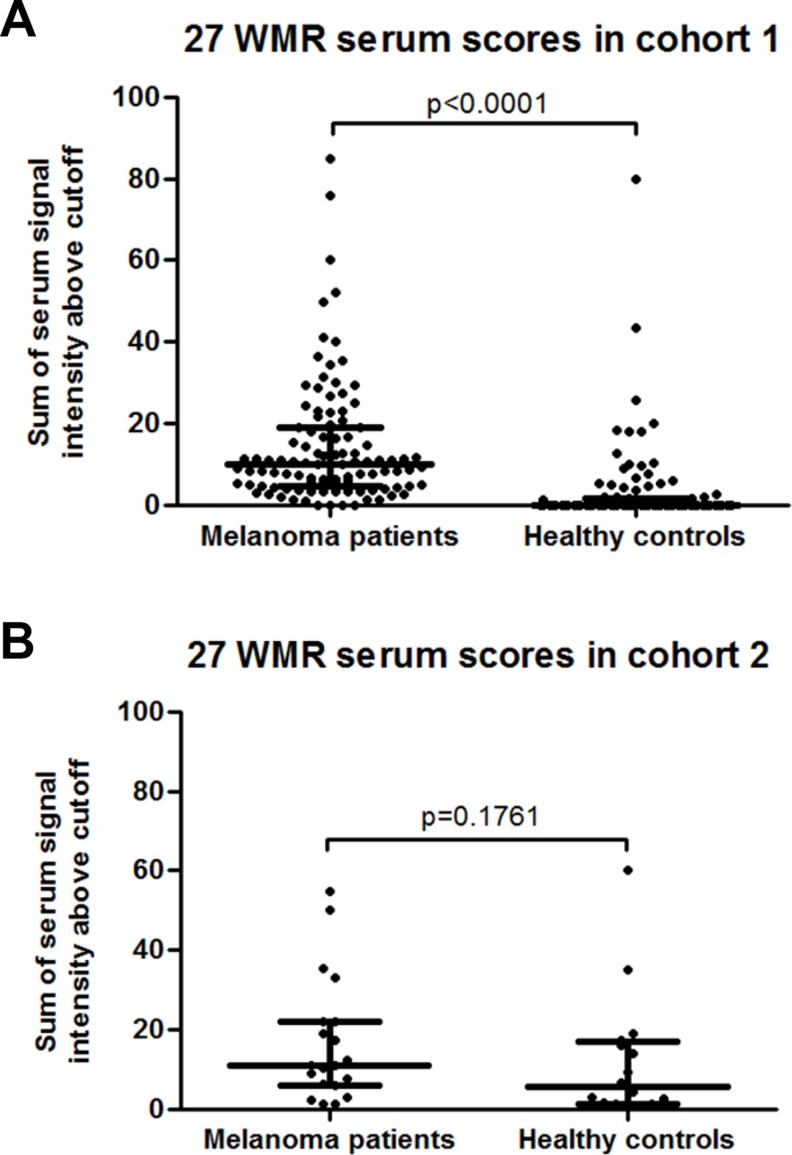
(**A**) Dot plot of melanoma patient and healthy control serum scores for the 27 melanoma-associated biomarkers with the highest weighted mean rank (WMR) score in cohort 1. (**B**) Dot plot of melanoma patient and healthy control serum scores for the 27 melanoma-associated biomarkers with the highest weighted mean rank (WMR) score in cohort 2.

Following random forest analysis of the data from cohort 1, the top 20 most influential markers for a diagnostic model were identified, and the most important AAbs were given a rank score of 20 and the least important marker a score of 1. This analysis was repeated 1000 times to generate 1000 random forests. When the top 20 markers of each of these 1000 forests were combined, a list of 27 unique biomarkers and their percent model inclusion frequency and average rank scores, were identified. The proportion of the appearance of each of these biomarkers in the 1000 top 20 AAb lists were then multiplied by the average rank score to obtain a weighted mean rank by which the overall importance of the biomarker for melanoma diagnosis was determined. In cohort 1, patient serum scores for these 27 antigens were again significantly higher with a median of 10.2 (IQR 4.7–19.1), than the healthy control median serum score of 0 (IQR 0–1.6, *p* < 0.0001, Figure 3A). However, there was no significant difference between patient and controls serum scores in cohort 2 (median of 11.2 (IQR 6.2–22.0) versus 5.5 (1.4–16.9), *p* = 0.176, Figure 3B), possibly due to the small sample size. Interferon regulator 4 (IRF4) was the most frequently included biomarker in the 1000 combinations and displayed the highest average rank score and was therefore the most important marker to contribute to the overall sensitivity and specificity in a combination of AAbs. As a single biomarker, IRF4 displayed a sensitivity of 5.8% at 100% specificity.

**Figure 4:**
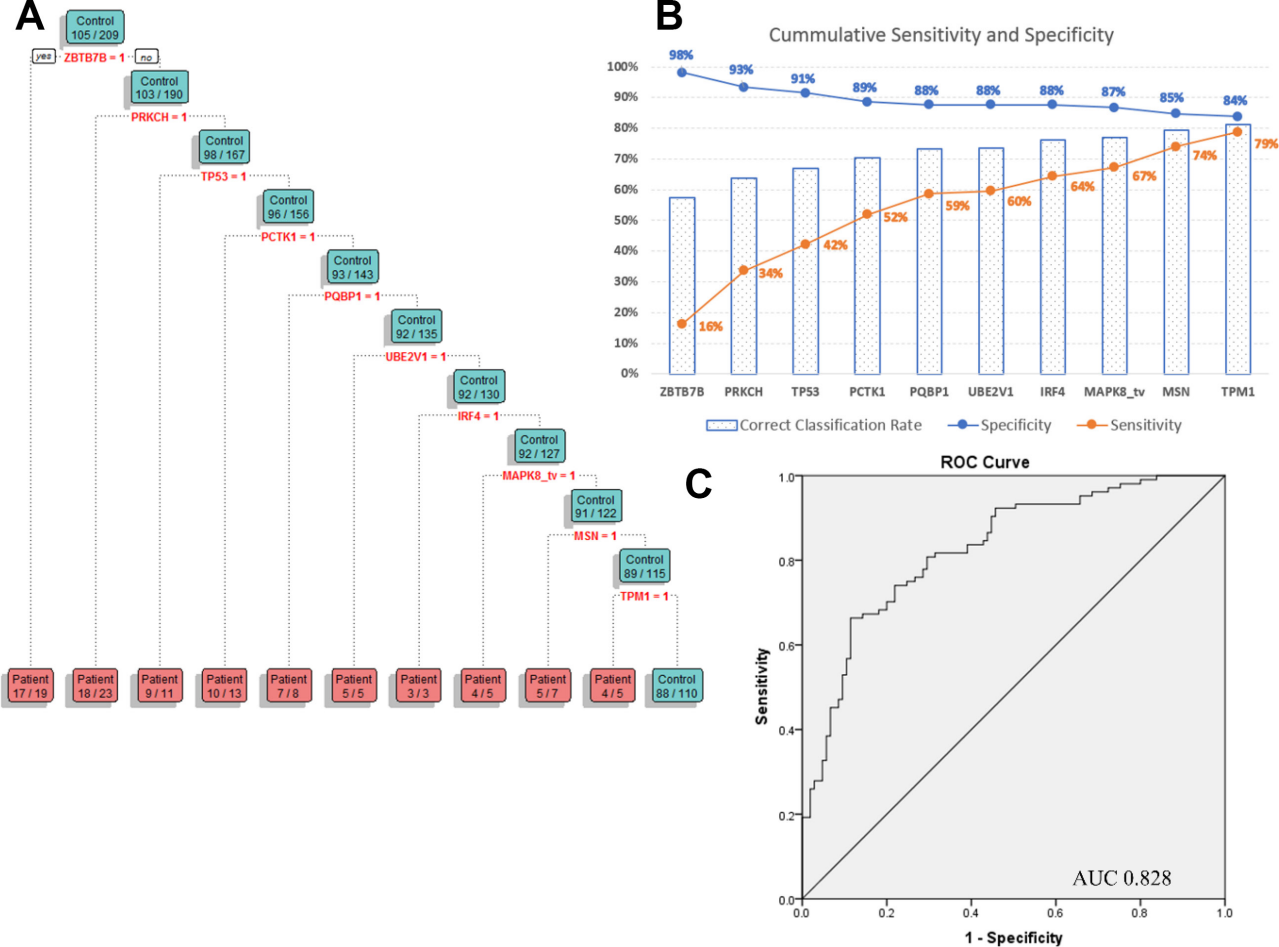
(**A**) Regression tree diagram of the best combination of the identified autoantibody biomarkers. (**B**) Cumulative sensitivity and specificity of the 10 AAb biomarker panel. (**C**) ROC curve and AUC of the biomarker combination in cohort 1.

Classification tree analysis was then applied to these 27 biomarkers and showed that the best combination of biomarkers ensuring an increased combined sensitivity and specificity for melanoma diagnosis, is a signature of 10 AAbs, including in order, ZBTB7B, PRKCH, TP53, PCTK1, PQBP1, UBE2V1, IRF4, MAPK8_tv2, MSN and TPM1, with a sensitivity of 79%, specificity of 84% and an AUC of 0.828 (Figure 4A–4C). The biomarkers included in the panel did not necessarily display the highest individual diagnostic potential (Supplementary Table 1.2). Instead, they are a combination, displaying a broader occurrence of positive seroreactivity for patient sera if a positive diagnosis is said to be represented by positive seroreactivity with at least one of the biomarkers in the combination.

### Breadth of autoantibody responses

The breadth of AAb responses against the protein microarray of 1627 proteins varied between samples. Positive autoantibody production in a sample was defined as a fluorescence reading above the protein associated cutoff. Positive autoantibody production to at least one of the proteins was observed in every study participant sample in cohort 1. Out of the 1627 antigens on the array, patient sera reacted with a median of 46.0 (IQR 36.0–70.0) antigens while healthy control sera reacted with a median of 48.0 (IQR 40.5–57.0) antigens (*p* = 0.857). All patient samples and 92.4% of healthy control samples reacted with at least one of the top 139 antigens. In total, a sum of 1426 positive antibody responses against the top 139 antigens were observed in the 104 patients while only 434 positive antibody responses were observed in the 105 healthy controls. A statistically significant difference was observed between the number of individual patient and healthy control sample AAb responses against the 139 antigens (median of 9.0 (IQR 6.0–22.0) versus 3.0 (IQR 1.0–4.0), respectively, *p* < 0.001). The median number of AAb responses was also significantly different between patient and healthy control samples (*p* < 0.001) for the identified AAb biomarker combination of 10 autoantibodies, with patient samples displaying a median of 1.0 (range 0–3.0) when compared the control median of 0 (range 0–2.0).

### Patient characteristics

Similar to other studies, we did not observe any significant differences or correlations in serum score or frequency of positive AAb reactions against the top 139 antigens when comparing age, gender, TNM stage, site of primary tumour, melanoma subtype, Breslow thickness, ulceration, mitotic rate, and history of multiple melanomas, non-melanoma skin cancers or other cancers (Supplementary Table 1.4). We did however observe a significantly lower number of AAb responses in patients with histologic regression features in their primary tumour than those who did not display features of regression (median and IQR of 7 (5.0–13.0) versus 11.5 (7.0–29.8), *p* = 0.010).

## DISCUSSION AND CONCLUSIONS

The benefit of early melanoma detection and timely surgical excision of the primary tumour is clear. Despite advances in diagnostic methods, screening large populations for melanoma remains inefficient due to the time required to screen each individual and due to a plethora of other limitations clinicians face in the current diagnosis of this cancer [4]. In particular, while individuals in countries with a high melanoma prevalence such as Australia and New Zealand are advised to maintain routine annual skin checks, there is no capacity for this practice given the limited number of dermatologists available in the country, especially in the rural regions in Australia [23]. Complementary diagnostic tools, such as blood tests, are needed to increase melanoma screening efficiency, provide diagnostic certainty and lower the overemphasis on invasive and expensive biopsies [24]. In fact, previous data has shown that only 5% of the total health care costs associated with melanoma are spent on the management of early melanoma disease including the costs of primary tumour diagnosis and excision, while the remaining 95% of all melanoma related health care costs are spent on the treatment and management of advanced melanoma [25], estimated to amount to $201 million annually in Australia [26]. Therefore, early detection and treatment could not only drastically improve patient 5-year survival rates to 99% [3], but also lower the financial burden of the disease on the health care system.

As blood samples are easily accessible from patients, various types of blood-based biomarkers have already been proposed to be utilised in a blood test for melanoma [5–7, 27], but none have yet demonstrated sufficient sensitivity to detect biological changes at the earliest stages of this malignancy. AAbs that bind to tumour-associated autoantigens, rise in serum levels at early disease stages, possibly due to a change in their expression, structural confirmation, presence of mutations or their release into the surrounding blood serum due to cancer cell lysis [9]. AAbs have already been shown to reside within the patient’s blood months to years prior to the clinical manifestation of the primary tumour [8, 28]. They have also been proposed to be valuable biomarkers for the early detection of many types of cancers and some are already being utilised in current diagnostic tests such as the EarlyCDT-Lung test for lung cancer detection and PAULA’s test for non-small cell lung cancer diagnosis [21, 29, 30]. In melanoma, AAbs have been suggested to be suitable prognostic biomarkers [31, 32], however very few studies have detailed their efficacy. In one study of an AAb signature for gastric cancer diagnosis, Zayakin and colleagues identified anti-DDX53, anti-MAGEA3 and anti-MAGEC1 antibodies in two samples with advanced melanoma that were subsequently excluded from further analysis [20].

Notably, few studies have investigated the presence of AAbs in early stage melanoma [17, 32] and those that have, commonly investigate the presence of AAbs against a list of common melanoma-associated targets compiled from a search of the literature, screen a pool of samples or utilise cell lines without performing rigorous screening of the individual patient autoimmune repertoire against an unbiased array of proteins. Hence to our knowledge, this is the first study identifying AAbs as diagnostic biomarkers in a large cohort of primary melanoma patients compared to healthy volunteers using a high-throughput functional protein microarray platform.

There are still no standardized guidelines for the analysis of protein microarray data. The approach stated by Gnjatic and colleagues, allowing the identification of biomarkers based not only on their fluorescent intensity levels but also their occurrence of positive seroreactivity in patients versus controls [17], appears to be utilised most frequently [33]. Using this approach in this study, we have successfully identified 139 potential melanoma diagnostic AAb biomarkers with individual sensitivity and specificity comparable to similar AAb studies [21]. Interestingly, most of the identified markers are novel and not known for their association with melanoma but are reactive against nuclear antigens such as transcription factors that may significantly influence cancer-related pathways. The inclusion of previously identified melanoma or cancer associated biomarkers such as VEGFb, p53, BAD, KIT MITF and MLANA [22] in the top 139 antigen list, further highlights the validity of our findings. Histologic tumour regression status significantly affected the number of positive AAb responses but although the correlation between regression and patient outcome has been investigated extensively, whether the presence of histologic regression features have a good or bad prognostic significance remains controversial and is to be elucidated [34]. Autoantibody responses were not affected by any other patient characteristic or tumour feature.

Most serum AAbs used as stand-alone diagnostic assays, show insufficient sensitivity and/or specificity to be utilised as accurate screening tools [35] and hence it is the combination of multiple reactive AAbs into a panel assay which provides the increased sensitivity and specificity. This approach has been successfully validated in several independent populations of various cancer types [36–39] as well as in this cohort of primary melanoma patients. To identify such combinations, here we proposed an analysis pipeline including random forest and decision tree analysis that adds to methods utilised by Gnjatic *et al.* 2009 [17], and enabled the identification of a 10 autoantibody biomarker signature with 79% sensitivity at 84% specificity for primary melanoma. One of these biomarkers is IRF4, a known lymphocyte specific transcription factor that negatively regulates Toll-like receptor (TLR) signalling, crucial for the activation of both the innate and adaptive immune response. Additionally, tumour suppressor p53 was included in the combination. In fact, the majority of research on autoantibodies in cancer has been focussed on p53 autoantibodies whose levels are commonly found to increase with cancer progression [40, 41], but are also detectable at early stages as in this study.

Although this analysis repeatedly showed that the best combination for melanoma diagnosis in our cohort is the identified signature of 10 AAbs, it is important to remember that there may be many other possible combinations of biomarkers generated using the list of 139 top antigens that may prove valuable for melanoma detection and should be investigated in the future. Most importantly, the panel identified here must be validated using larger cohorts of melanoma patients and should include patients with other types of cancer or autoimmune diseases to ascertain whether the combination is melanoma specific. This may be performed using alternate high- throughput platforms for the simultaneous testing of these antigens against a vast number of samples to detect AAb levels, such as the bead-based Luminex platform.

Finally, since it is known that autoantigens that are modified before or during the course of tumour formation and progression in cancer, can stimulate the immune response in patients when they are released from tumour cells [42] and that immune responses have been observed to be responsible for tumour growth promotion, but also prevention in a process called immunoediting [43, 44], the fact that some of the top 139 biomarker associated signalling pathways are involved in the immune response, is consistent with the immunoediting theory [43] and leads to the proposal that the identified AAb biomarkers may be utilised in future therapeutic interventions to treat or monitor advanced melanoma and aid in the early diagnosis and surveillance of patients with melanoma.

## MATERIALS AND METHODS

### Study participants

A total of 245 study participants were recruited by collaborating clinicians and the principal researchers (minimum sample size required per group to achieve at least an AUC of 0.66 at an alpha of 0.05 and 80% power is *n =* 47 per group). All participants provided informed consent to participate in this study, previously approved by the Edith Cowan University Ethics Committee (numbers 11543 and 12066). Patients were diagnosed by routine pathological examination of their excised primary tumour and staged according to the TNM staging system for melanoma according to the American Joint Committee on Cancer (AJCC) guidelines [3]. Healthy volunteers were defined as never having been diagnosed with melanoma, any other type of cancer or any autoimmune disease. The study cohort 1 consisted of 104 early stage melanoma patients (classified as TNM stages *in situ*, I and II) and 105 healthy volunteers. A smaller cohort including 20 early stage melanoma patients (classified as TNM stages *in situ* and I only) and 16 healthy volunteers (cohort 2) was also utilised for validation.

### Sample collection

A once-off blood sample was obtained from all study participants. For the majority of melanoma patients, the sample was obtained within 1 month of patient primary tumour diagnosis and excision, some samples were obtained within a maximum of 3 months of diagnosis. Venous blood from all study participants was collected into one 8.5 ml serum separator tube (SST) (BD, New Jersey, United States). The blood was allowed to clot at room temperature for a minimum of 30 minutes and centrifuged at 1600 g for 10 mins. A small number of healthy volunteer samples (*n =* 8) which had been collected into EDTA tubes were analysed from plasma. These samples were included in this study as serum and plasma samples have previously been found to yield comparable results in functional protein microarray studies [17]. Following centrifugation, serum was aliquoted as soon as possible but within 24 hours and stored at −80° C.

### Protein microarray profiling

The functional protein microarray was developed and constructed by Oxford Gene Technology (OGT), Oxfordshire, United Kingdom, as described previously [17]. Patient or control serum samples were diluted 1:200 in 2 ml buffer (0.1% Triton X100 (v/v), 0.1% BSA (w/v) in PBS) and applied to the array (one array per sample). The arrays were incubated in Quadriperm dishes (Greiner BioOne, Stonehouse, UK) and placed on a horizontal shaker at 50 rpm for a period of 2 hours at room temperature. After several washes, anti-human IgG was diluted 1:1000 in assay buffer and Cy3-rabbit anti-human IgG (Dako Cytomation) by incubation for 2 hours at room temperature according to the manufacturer´s recommendations. The array was washed again and dried by centrifugation. All arrays were scanned at 10 µm resolution using a microarray scanner (Axon 4200AL with GenePix Pro Software, Molecular Devices, Sunnyvale, CA, USA) and fluorescence of labelled IgG was detected according to the manufacturer’s instructions. Images were saved as 16-bit tiff files and analysis was performed using GenePix software [17]. Interaction between microarray antigens and serum autoantibodies was detected as fluorescence of the bound fluorescently-labelled IgG at the protein specific position on the microarray. The intensity of fluorescence is proportional to the amount of autoantibody present in the serum [17, 45]. Local background obtained from control spots on the array was subtracted automatically and relative fluorescence units (rfu) for each microarray spot were recorded. Each antigen was immobilised in quadruplicate on the array. The median rfu for the four readings of each antigen was utilised for further analysis. A reference serum was included in each microarray experiment run. Arrays that did not pass quality control tests were repeated or the spots were realigned in the software or excluded. Thereafter, arrays were excluded from the analysis if they did not pass quality control.

Samples were tested by Sengenics, University of Malaysia, Kuala Lumpur, Malaysia, for the microarray screening of cohort 1 or by Oxford Gene Technology (OGT), Oxfordshire, United Kingdom, for the microarray screening of cohort 2. Both locations utilised the same Immunome^™^ microarray platform (Sengenics). OGT and Sengenics staff were blinded to the fact that, for the purpose of cross-validation between the two screening sites, identical aliquots from 16 randomly selected patients and 11 healthy control samples were screened at both sites, and showed comparable results (Spearman rho > 0.5, Supplementary Table 1.1) enabling the use of cohort 2 as an independent validation cohort.

### Statistical analysis

#### Data normalisation

Intra- and inter-array data normalisation was performed to ensure data comparability between samples. First, the overall median value of all median rfu values of the 1627 printed proteins (excluding data from controls spots) was calculated and intra-array normalisation was achieved by dividing the median of the quadruplicate spots of each protein on the array, by the overall median value of all the proteins on the array in each sample. Inter-array normalisation was achieved by utilisation of the *normalize.quantiles* package [46] in R.

### Selection of melanoma associated autoantibodies

Once normalised, data analysis was performed as described by Gnjatic *et al.* 2009 [17] to determine the proteins with the highest and most frequent seroreactivity in patient samples relative to healthy volunteer sera. For this, the interquartile range (IQR) was calculated for each protein to establish a cutoff. This cutoff (2.5 × IQR above the 75th percentile) was used to dichotomise the data, whereby a value was defined as positive (for seroreactivity) if it was above the cutoff; otherwise it was defined as negative. This criterion was used to ensure false positive data was minimised while providing increased specificity and sensitivity [17]. For cases with positive seroreactivity, the ratio between the signal and cutoff (S/C ratio) was calculated. Thereafter, the average S/C ratio was calculated per protein for each cohort, i.e. melanoma patient or healthy control.

Finally, a “biomarker score” was assigned to each protein by multiplying the number of positive samples by the cubic root of the corresponding S/C ratio average for each cohort. This score is a reflection of the strength and frequency of the signal in patients relative to healthy subjects. The proteins were then ranked based on the differences in the biomarker scores (patients–healthy controls). A large AAb biomarker score (>5) indicates that most seroreactivity is attributable to the patients [17]. This reduced the number of potential diagnostic melanoma autoantibody biomarkers from 1627 to 139 in cohort 1 (Supplementary Table 1.2).

### Selection of biomarker panel

The analysis proposed here furthers the methodology proposed by Gnjatic *et al.* 2009 [17] by subsequently exploring combinations of identified biomarkers for early melanoma detection, rather than individual biomarkers, to achieve increased combined sensitivity and specificity. The *classification tree* method was selected for this task and this analysis was performed using data from cohort 1 only as cohort 2 was not sufficiently powered. The number of variables (i.e. 139 antigens) at this stage was still reasonably large relative to the overall sample size. To avoid the possibility of overfitting, a two-stage process was utilised, as follows:

Stage l involved the use of random forest regression analysis [18] for identifying key biomarker proteins and to further reduce the number of biomarkers in contention for the next modelling stage. Stage 2 utilised the classical classification tree approach [19] to develop a tree model based on the reduced list of biomarkers.

All analyses were implemented with the R software package (Version 3.2.2). The key R packages used were *randomForest* [47] (based on the original Fortran code by Breiman and Cutler), *rpart* [48] and *caret* [49].

### Additional statistical analysis

The previously established antigen-associated cutoffs were, additionally, used to determine (1) the number of positive AAbs (i.e. above the cutoffs) and (2) the serum score for each sample. The “serum score” is the sum of all signal intensities above the said cutoffs. To test whether these data were approximately normally distributed, a Shapiro-Wilk’s test (*p* > 0.05) [50], visual inspection of histograms, normal Q-Q plots and box-plots were performed. As the majority of the data in this study were not normally distributed, non-parametric methods were utilised. The Mann–Whitney *U* test was used to test for differences in serum scores between patients and controls. Spearman rho correlation analysis was used to test for a statistical relationship between patient age, tumour Breslow thickness and mitotic rate with the number of positive AAbs and patient serum scores. Differences in patient serum score or the number of positive AAbs with regards to gender, tumour ulceration, histologic regression, history of multiple diagnosed melanoma in the patient’s lifetime, a history of non-melanoma skin cancer or other cancer were assessed by Mann–Whitney *U* test. Serum score or number of positive AAb differences with regards to tumour TNM stage, primary tumour site, melanoma subtype and Clark level were assessed by Kruskal-Wallis analysis. Sensitivity and specificity of individual and combinations of AAbs were evaluated by ROC. These analyses were performed using Microsoft Excel, IBM SPSS statistical software (version 22.0) and GraphPad Prism (version 5). A *p*-value of < 0.05 was defined as statistically significant.

Finally, to explore the biological relevance of the identified autoantibody biomarkers and their interactions, we submitted the top 139 antigen names to the online functional protein association network, STRING, to explore the main shared antigen pathways at medium protein interaction confidence of 0.400. The submitted protein names are identical to Supplementary Table 1.2 while protein PCTK1 and SDCCAD10 were searched by their alternative names CDK16 and CWC27, respectively.

## SUPPLEMENTARY MATERIALS FIGURE AND TABLES



















## Abbreviations

AAbs: autoantibodies
AJCC: American Joint Committee on Cancer
SST: serum separator tube
OGT: Oxford Gene Technology
rfu: relative fluorescence units
AUC: area under the curve
ROC: receiver operating curve
IQR: inter quartile range
SSM: superficial spreading melanoma
NM: nodular melanoma
LMM: lentigo maligna melanoma
ALM: acral lentiginous melanoma
