# Supplementary material for: A diagnostic autoantibody signature for primary cutaneous melanoma
Source: Oncotarget. 2018 Jul 17;9(55):30539–51. doi: 10.18632/oncotarget.25669 (PMC6078131; doi:10.18632/oncotarget.25669)
Supplement: Supplementary file 1 [file oncotarget-09-30539-s001.pdf]

A diagnostic autoantibody signature for primary cutaneous melanoma

SUPPLEMENTARY MATERIALS

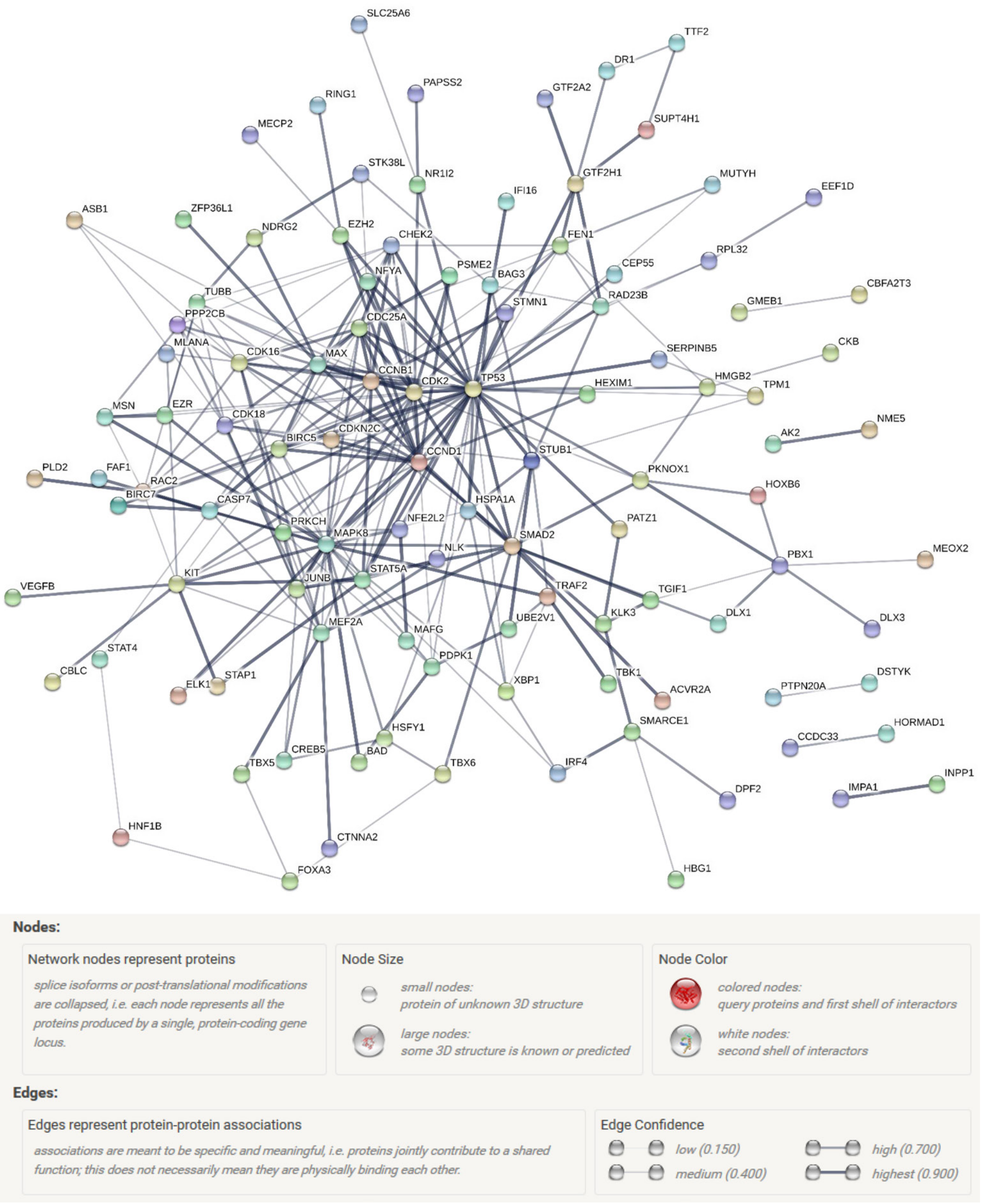

Supplementary Figure 1: STRING protein interaction network for the top 139 individual biomarkers. This STRING protein interaction network displays possible interactions between the 139 top proteins. Altogether, 260 possible interactions have been identified between the submitted proteins at a confidence >0.400. The thickness of the line indicates the level of confidence. Proteins without identified interactions were not included in this graph.

Supplementary Table 1.1: Comparison of 27 samples that were part of both study cohorts and tested against the same microarray at two different testing sites. See Supplementary_Table_1.1

Supplementary Table 1.2: Top 139 autoantibodies in cohort 1 displaying an overall biomarker score of >5. See Supplementary_Table_1.2

Supplementary Table 1.3: STRING protein description for the top 139 individual biomarkers. See Supplementary_Table_1.3

Supplementary Table 1.4: Patient serum scores and frequency of positive AAb reactions against the top 139 antigens compared with patient characteristics and pathological features of the primary melanoma tumour

	number of AAbs	serum score
Age	0.387	0.327
Gender	0.724	0.860
TNM stage	0.904	0.471
Site of primary tumour	0.151	0.583
Melanoma subtype	0.153	0.297
Breslow thickness	0.304	0.191
Clark level	0.338	0.544
Ulceration	0.706	0.762
Mitotic rate	0.161	0.451
Regression	**0.010**	0.101
History of multiple CM	0.964	0.903
History of NMSC	0.465	0.823
History of other cancer	0.593	0.577

Significance numbers are rounded to 3 decimals; results obtained through Spearman rho correlation, Mann-Whitney-U or Kruskal-Wallis analysis, $p < 0.05$ is deemed significant.

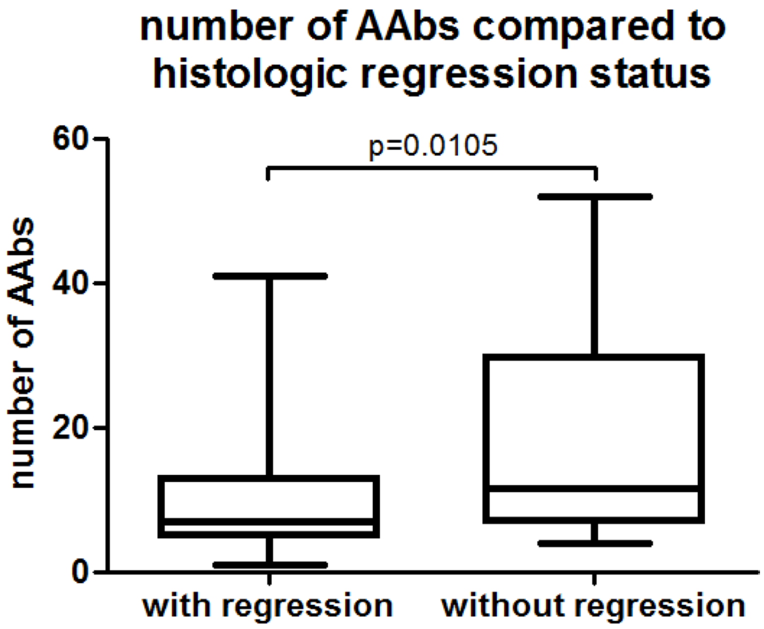

Supplementary Table 2.1: Biological function. See Supplementary_Table_2.1

Supplementary Table 2.2: Molecular pathway. See Supplementary_Table_2.2

Supplementary Table 2.3: Cellular location. See Supplementary_Table_2.3

Supplementary Table 2.4: KEGG pathways. See Supplementary_Table_2.4

Supplementary Table 2.5: STRING protein interactions. See Supplementary_Table_2.5
