# Supplementary material for: A diagnostic autoantibody signature for primary cutaneous melanoma
Source: Oncotarget. 2018 Jul 17;9(55):30539–51. doi: 10.18632/oncotarget.25669 (PMC6078131; doi:10.18632/oncotarget.25669)
Supplement: Supplementary file 2 [file oncotarget-09-30539-s002.docx]

Supplementary Table 1.1: Comparison of 27 samples that were part of both study cohorts and tested against the same microarray at two different testing sites. The correlation coefficient and p-value were obtained by investigating the correlation between the two results from the different cohorts/ testing site against the identified top 139 antigens

| **Sample ID** | **Correlation coefficient (r)** | **p-value** |
| --- | --- | --- |
| **CM17** | 0.769 | **<0.001** |
| **CM1** | 0.545 | **<0.001** |
| **CM10** | 0.849 | **<0.001** |
| **CM12** | 0.076 | 0.372 |
| **CM13** | 0.847 | **<0.001** |
| **CM14** | 0.605 | **<0.001** |
| **CM15** | 0.803 | **<0.001** |
| **CM19** | 0.750 | **<0.001** |
| **CM22** | 0.711 | **<0.001** |
| **CM24** | 0.851 | **<0.001** |
| **CM25** | 0.605 | **<0.001** |
| **CM3** | 0.586 | **<0.001** |
| **CM5** | 0.921 | **<0.001** |
| **CM6** | 0.808 | **<0.001** |
| **CM7** | 0.892 | **<0.001** |
| **CM8** | 0.712 | **<0.001** |
| **HC14** | 0.583 | **<0.001** |
| **HC17** | 0.579 | **<0.001** |
| **HC20** | 0.687 | **<0.001** |
| **HC22** | 0.699 | **<0.001** |
| **HC23** | 0.374 | **<0.001** |
| **HC25** | 0.628 | **<0.001** |
| **HC27** | 0.799 | **<0.001** |
| **HC28** | 0.678 | **<0.001** |
| **HC29** | 0.771 | **<0.001** |
| **HC30** | 0.220 | **0.009** |
| **HC32** | 0.487 | **<0.001** |
| A total of 16 patient (CM) and 11 healthy control (HC) samples were run in both study cohorts, r and p-values obtained through Spearman's Rho correlation, p<0.05 was considered significant (bold) | | |

Serum aliquots of 27 identical samples (16 patient and 11 healthy control samples) were included in both study cohorts. Spearman rho’s correlation was utilised to assess the reproducibility of the microarray data and to test whether results obtained from the two cohorts for the 27 samples correlated between the two testing sites. The results from each site for each of the 139 antigens correlated strongly (rho= 0.5-1, p<0.05) in 23/27 samples (85.2%) while a moderate correlation (r= 0.3-0.5, p<0.05) was observed in 2/27 samples (7.4%) and weak correlation (r= 0.1-0.3, p=0.37) in 1/27 samples (3.7%). Since 92.9% of the re-tested samples displayed moderate to strong correlation in their twice normalised microarray data for all top 139 biomarkers, it is reasonable to conclude that the results are comparable between the two cohorts and testing sites and cohort 2 may serve as an individual validation cohort whilst considering its smaller sample size and hence lower statistical power.
