# Supplementary material for: A diagnostic autoantibody signature for primary cutaneous melanoma
Source: Oncotarget. 2018 Jul 17;9(55):30539–51. doi: 10.18632/oncotarget.25669 (PMC6078131; doi:10.18632/oncotarget.25669)
Supplement: Supplementary file 3 [file oncotarget-09-30539-s003.docx]

**Supplementary Table 1.2** Top 139 autoantibodies in cohort 1 displaying an overall biomarker score of >5

| **Protein** | **Sensitivity (%)** | **Specificity (%)** | **cutoff** | **average signal to cutoff ratio for patients** | **average signal to cutoff ratio for healthy volunteers** | **overall score patients** | **overall score healthy volunteers** | **Biomarker score (patients - healthy volunteers)** |
| --- | --- | --- | --- | --- | --- | --- | --- | --- |
|  |  |  |  |  |  |  |  |  |
|  |  |  |  |  |  |  |  |  |
| **PRKCH** | 18.3 | 95.2 | 1.21 | 3.73 | 1.44 | 29.47 | 5.65 | 23.82 |
| **PCTK3** | 16.3 | 96.2 | 1.25 | 7.42 | 20.62 | 33.16 | 10.97 | 22.19 |
| **DPF2** | 15.4 | 96.2 | 1.47 | 5.59 | 4.75 | 28.40 | 6.72 | 21.68 |
| **KIT** | 17.3 | 95.2 | 1.06 | 2.50 | 2.36 | 24.42 | 6.65 | 17.77 |
| **KLK3** | 16.3 | 95.2 | 1.25 | 2.53 | 2.24 | 23.16 | 6.54 | 16.62 |
| **STK38L** | 15.4 | 95.2 | 1.60 | 3.21 | 2.85 | 23.60 | 7.09 | 16.50 |
| **STMN1** | 16.3 | 92.4 | 2.65 | 3.38 | 1.47 | 25.51 | 9.11 | 16.41 |
| **DLX3** | 16.3 | 98.1 | 0.96 | 1.11 | 1.14 | 17.60 | 2.09 | 15.51 |
| **ZBTB7B** | 16.3 | 98.1 | 0.92 | 1.13 | 1.49 | 17.71 | 2.29 | 15.43 |
| **ASB1** | 16.3 | 95.2 | 1.17 | 2.00 | 1.76 | 21.43 | 6.04 | 15.40 |
| **CASP7** | 14.4 | 95.2 | 1.64 | 4.27 | 6.22 | 24.33 | 9.19 | 15.14 |
| **RAC2** | 16.3 | 96.2 | 1.10 | 1.56 | 1.78 | 19.70 | 4.85 | 14.85 |
| **HBG1** | 13.5 | 98.1 | 1.14 | 1.73 | 1.16 | 16.81 | 2.10 | 14.71 |
| **NFE2L2** | 15.4 | 98.1 | 1.14 | 1.11 | 1.09 | 16.56 | 2.06 | 14.50 |
| **ELK1** | 12.5 | 97.1 | 3.52 | 2.64 | 1.65 | 17.97 | 3.54 | 14.43 |
| **EZH2** | 15.4 | 98.1 | 1.03 | 1.09 | 1.33 | 16.47 | 2.20 | 14.27 |
| **PDGFRL** | 18.3 | 93.3 | 1.07 | 1.64 | 1.58 | 22.42 | 8.15 | 14.27 |
| **HRH2** | 16.3 | 93.3 | 1.20 | 2.54 | 2.14 | 23.19 | 9.02 | 14.17 |
| **TP53** | 10.6 | 98.1 | 5.79 | 3.15 | 1.19 | 16.12 | 2.12 | 14.01 |
| **EXT2** | 17.3 | 95.2 | 1.09 | 1.20 | 1.16 | 19.12 | 5.25 | 13.87 |
| **PYGO2** | 13.5 | 94.3 | 1.90 | 3.29 | 1.83 | 20.83 | 7.34 | 13.48 |
| **SERPINB5** | 14.4 | 98.1 | 1.02 | 1.14 | 1.32 | 15.68 | 2.20 | 13.48 |
| **NR1I2** | 13.5 | 99 | 0.99 | 1.11 | 1.11 | 14.49 | 1.04 | 13.46 |
| **XYLB** | 16.3 | 95.2 | 1.48 | 1.32 | 1.24 | 18.67 | 5.37 | 13.31 |
| **PHIP** | 16.3 | 95.2 | 1.22 | 1.31 | 1.30 | 18.59 | 5.46 | 13.13 |
| **CCNB1** | 10.6 | 96.2 | 3.10 | 4.43 | 2.17 | 18.07 | 5.18 | 12.89 |
| **STAT5A** | 13.5 | 97.1 | 1.05 | 1.44 | 1.09 | 15.81 | 3.09 | 12.72 |
| **RAD23B** | 15.4 | 94.3 | 3.16 | 1.92 | 1.82 | 19.88 | 7.33 | 12.55 |
| **IFI16** | 13.5 | 98.1 | 1.03 | 1.14 | 1.27 | 14.62 | 2.16 | 12.45 |
| **TUBB** | 10.6 | 97.1 | 1.18 | 3.40 | 2.78 | 16.54 | 4.22 | 12.33 |
| **SMARCE1** | 16.3 | 94.3 | 0.80 | 1.34 | 1.28 | 18.73 | 6.52 | 12.21 |
| **MSN** | 9.6 | 98.1 | 2.42 | 2.92 | 1.90 | 14.29 | 2.48 | 11.81 |
| **ZNF169** | 10.6 | 98.1 | 1.31 | 2.34 | 2.77 | 14.60 | 2.81 | 11.79 |
| **HSPA1A** | 10.6 | 97.1 | 1.48 | 2.55 | 1.28 | 15.04 | 3.26 | 11.78 |
| **DR1** | 14.4 | 91.4 | 1.71 | 3.07 | 1.40 | 21.80 | 10.07 | 11.73 |
| **SLC25A6** | 14.4 | 95.2 | 1.09 | 1.42 | 1.48 | 16.86 | 5.69 | 11.17 |
| **TRAF2** | 9.6 | 98.1 | 4.39 | 2.13 | 1.18 | 12.87 | 2.11 | 10.76 |
| **BAD** | 16.3 | 94.3 | 1.45 | 2.82 | 11.15 | 24.03 | 13.41 | 10.62 |
| **PKNOX1** | 9.6 | 98.1 | 6.32 | 2.15 | 2.00 | 12.91 | 2.52 | 10.39 |
| **PCTK1** | 11.5 | 97.1 | 6.05 | 1.65 | 2.35 | 14.19 | 3.99 | 10.20 |
| **FOXR2** | 17.3 | 91.4 | 5.94 | 1.98 | 2.70 | 22.60 | 12.53 | 10.06 |
| **EZR** | 7.7 | 98.1 | 2.09 | 3.39 | 1.22 | 12.02 | 2.14 | 9.88 |
| **PPP2CB** | 12.5 | 96.2 | 1.45 | 1.33 | 1.41 | 14.31 | 4.49 | 9.82 |
| **UBE2V1** | 4.8 | 99 | 1.70 | 9.97 | 1.27 | 10.76 | 1.08 | 9.68 |
| **JUNB** | 10.6 | 98.1 | 1.18 | 1.20 | 1.16 | 11.69 | 2.10 | 9.60 |
| **BIRC7** | 12.5 | 95.2 | 3.46 | 1.63 | 1.59 | 15.31 | 5.83 | 9.48 |
| **STK10** | 9.6 | 98.1 | 1.81 | 1.53 | 1.31 | 11.53 | 2.19 | 9.35 |
| **DSTYK** | 13.5 | 95.2 | 1.18 | 1.10 | 1.12 | 14.45 | 5.19 | 9.26 |
| **MTERF** | 10.6 | 98.1 | 0.99 | 1.13 | 1.86 | 11.46 | 2.46 | 9.00 |
| **FEN1** | 8.7 | 92.4 | 4.47 | 8.88 | 1.86 | 18.64 | 9.85 | 8.79 |
| **KLF12** | 7.7 | 100 | 1.24 | 1.28 | 0.00 | 8.68 | 0.00 | 8.68 |
| **MEF2A** | 9.6 | 96.2 | 2.23 | 2.97 | 3.01 | 14.37 | 5.77 | 8.60 |
| **SCFD1** | 8.7 | 97.1 | 1.49 | 2.37 | 1.46 | 12.00 | 3.40 | 8.60 |
| **ZNF444** | 8.7 | 99 | 1.08 | 1.21 | 1.06 | 9.60 | 1.02 | 8.58 |
| **STAP1** | 13.5 | 92.4 | 3.61 | 2.29 | 1.93 | 18.45 | 9.96 | 8.49 |
| **CDK2** | 9.6 | 96.2 | 3.94 | 2.41 | 1.86 | 13.41 | 4.92 | 8.49 |
| **NFYA** | 8.7 | 98.1 | 1.30 | 1.62 | 1.15 | 10.56 | 2.10 | 8.47 |
| **TGIF1** | 8.7 | 99 | 1.26 | 1.12 | 1.09 | 9.36 | 1.03 | 8.33 |
| **RPL32** | 10.6 | 97.1 | 1.28 | 1.11 | 1.10 | 11.38 | 3.10 | 8.29 |
| **DLX1** | 7.7 | 100 | 1.09 | 1.10 | 0.00 | 8.26 | 0.00 | 8.26 |
| **XBP1** | 9.6 | 98.1 | 1.07 | 1.17 | 1.57 | 10.54 | 2.32 | 8.22 |
| **IMPA1** | 8.7 | 99 | 1.51 | 1.08 | 1.08 | 9.22 | 1.03 | 8.20 |
| **PLD2** | 9.6 | 98.1 | 1.06 | 1.07 | 1.17 | 10.23 | 2.11 | 8.12 |
| **ACVR2A** | 15.4 | 90.5 | 2.23 | 2.18 | 2.03 | 20.75 | 12.66 | 8.09 |
| **PQBP1** | 9.6 | 97.1 | 2.99 | 1.40 | 1.42 | 11.18 | 3.37 | 7.81 |
| **TTF2** | 5.8 | 100 | 1.27 | 2.16 | 0.00 | 7.76 | 0.00 | 7.76 |
| **USH1C** | 7.7 | 98.1 | 4.74 | 1.90 | 1.42 | 9.91 | 2.25 | 7.67 |
| **HEXIM1** | 7.7 | 97.1 | 1.82 | 2.43 | 1.11 | 10.76 | 3.10 | 7.66 |
| **LRRFIP2** | 10.6 | 95.2 | 4.45 | 2.15 | 2.32 | 14.19 | 6.62 | 7.58 |
| **CEP55** | 7.7 | 99 | 2.29 | 1.21 | 1.01 | 8.52 | 1.00 | 7.52 |
| **SCAND1** | 5.8 | 97.1 | 1.42 | 5.65 | 1.21 | 10.69 | 3.20 | 7.49 |
| **VEGFB** | 7.7 | 97.1 | 3.24 | 2.75 | 2.03 | 11.21 | 3.80 | 7.41 |
| **HEYL** | 6.7 | 100 | 1.20 | 1.18 | 0.00 | 7.41 | 0.00 | 7.41 |
| **RQCD1** | 6.7 | 99 | 2.71 | 1.81 | 1.53 | 8.53 | 1.15 | 7.38 |
| **SDCCAG10** | 11.5 | 93.3 | 3.08 | 2.16 | 1.56 | 15.50 | 8.12 | 7.38 |
| **MLANA** | 5.8 | 98.1 | 2.59 | 4.27 | 1.77 | 9.73 | 2.42 | 7.32 |
| **HNF1B** | 6.7 | 100 | 1.16 | 1.14 | 0.00 | 7.31 | 0.00 | 7.31 |
| **MAPK8_tv2** | 5.8 | 99 | 2.60 | 2.72 | 1.26 | 8.37 | 1.08 | 7.30 |
| **PSME2** | 6.7 | 98.1 | 2.01 | 2.42 | 1.21 | 9.40 | 2.13 | 7.27 |
| **NDRG2** | 7.7 | 98.1 | 2.12 | 1.63 | 1.24 | 9.41 | 2.15 | 7.26 |
| **FOXA3** | 6.7 | 100 | 1.31 | 1.11 | 0.00 | 7.26 | 0.00 | 7.26 |
| **CKB** | 3.8 | 100 | 3.68 | 5.94 | 0.00 | 7.24 | 0.00 | 7.25 |
| **ZNF449** | 7.7 | 99 | 1.17 | 1.07 | 1.02 | 8.19 | 1.01 | 7.18 |
| **PBX1** | 8.7 | 98.1 | 1.06 | 1.07 | 1.05 | 9.21 | 2.03 | 7.18 |
| **TPM1** | 8.7 | 97.1 | 9.02 | 1.86 | 2.19 | 11.06 | 3.90 | 7.17 |
| **NME5** | 8.7 | 97.1 | 1.63 | 2.17 | 3.87 | 11.65 | 4.71 | 6.94 |
| **GTF2A2** | 8.7 | 98.1 | 1.08 | 1.05 | 1.45 | 9.16 | 2.26 | 6.90 |
| **CCND1** | 4.8 | 100 | 1.66 | 2.53 | 0.00 | 6.81 | 0.00 | 6.81 |
| **PAPSS2** | 7.7 | 98.1 | 5.97 | 1.67 | 2.59 | 9.49 | 2.75 | 6.75 |
| **STAT4** | 7.7 | 98.1 | 2.59 | 1.57 | 2.25 | 9.31 | 2.62 | 6.68 |
| **CBFA2T3** | 15.4 | 89.5 | 4.14 | 2.80 | 3.01 | 22.54 | 15.89 | 6.66 |
| **HMGB2** | 8.7 | 94.3 | 1.89 | 2.93 | 1.19 | 12.88 | 6.35 | 6.53 |
| **CCDC33** | 8.7 | 97.1 | 2.57 | 1.85 | 3.46 | 11.04 | 4.54 | 6.51 |
| **AK2** | 6.7 | 98.1 | 2.29 | 2.16 | 2.25 | 9.04 | 2.62 | 6.42 |
| **SMAD2** | 7.7 | 98.1 | 3.61 | 1.44 | 2.23 | 9.03 | 2.61 | 6.42 |
| **FMR1NB** | 6.7 | 99 | 1.53 | 1.13 | 1.04 | 7.30 | 1.01 | 6.29 |
| **FAF1** | 9.6 | 95.2 | 2.89 | 2.30 | 2.68 | 13.21 | 6.95 | 6.26 |
| **CREB5** | 6.7 | 99 | 1.29 | 1.11 | 1.03 | 7.24 | 1.01 | 6.23 |
| **ZFP36L1** | 6.7 | 99 | 1.20 | 1.11 | 1.05 | 7.24 | 1.01 | 6.22 |
| **IRF4** | 5.8 | 100 | 2.59 | 1.11 | 0.00 | 6.22 | 0.00 | 6.22 |
| **PTPN20A** | 14.4 | 89.5 | 3.87 | 2.46 | 2.08 | 20.26 | 14.04 | 6.21 |
| **C1orf216** | 5.8 | 98.1 | 2.58 | 2.57 | 1.02 | 8.22 | 2.01 | 6.21 |
| **HSFY1** | 5.8 | 100 | 1.07 | 1.09 | 0.00 | 6.17 | 0.00 | 6.17 |
| **KIF9** | 4.8 | 98.1 | 1.13 | 4.89 | 1.57 | 8.49 | 2.32 | 6.17 |
| **RING1** | 5.8 | 99 | 1.32 | 1.70 | 1.03 | 7.17 | 1.01 | 6.15 |
| **PRDM4** | 6.7 | 99 | 1.13 | 1.22 | 2.31 | 7.48 | 1.32 | 6.15 |
| **MAFG** | 7.7 | 98.1 | 1.16 | 1.14 | 1.35 | 8.36 | 2.21 | 6.15 |
| **MECP2** | 7.7 | 98.1 | 1.09 | 1.11 | 1.24 | 8.29 | 2.15 | 6.14 |
| **HOXB6** | 7.7 | 98.1 | 1.09 | 1.14 | 1.41 | 8.36 | 2.24 | 6.11 |
| **MUTYH** | 6.7 | 99 | 1.09 | 1.08 | 1.59 | 7.17 | 1.17 | 6.01 |
| **CDC25A** | 4.8 | 100 | 1.72 | 1.64 | 0.00 | 5.90 | 0.00 | 5.90 |
| **CDKN2C** | 5.8 | 99 | 1.88 | 1.53 | 1.23 | 6.92 | 1.07 | 5.85 |
| **SUPT4H1** | 2.9 | 100 | 1.83 | 6.94 | 0.00 | 5.72 | 0.00 | 5.72 |
| **CHEK2** | 5.8 | 99 | 1.71 | 1.49 | 1.87 | 6.85 | 1.23 | 5.62 |
| **BIRC5** | 5.8 | 99 | 2.18 | 1.35 | 1.30 | 6.64 | 1.09 | 5.55 |
| **INPP1** | 6.7 | 98.1 | 1.53 | 2.92 | 11.15 | 10.01 | 4.47 | 5.54 |
| **TXN2** | 12.5 | 91.4 | 3.37 | 2.16 | 1.97 | 16.81 | 11.28 | 5.53 |
| **CBLC** | 4.8 | 99 | 3.14 | 2.51 | 2.04 | 6.79 | 1.27 | 5.52 |
| **ANXA11** | 8.7 | 94.3 | 1.76 | 2.58 | 1.51 | 12.35 | 6.89 | 5.46 |
| **MAX** | 8.7 | 93.3 | 2.59 | 3.71 | 1.78 | 13.94 | 8.48 | 5.46 |
| **SLCO6A1** | 7.7 | 97.1 | 1.59 | 1.29 | 1.38 | 8.71 | 3.34 | 5.37 |
| **EEF1D** | 6.7 | 98.1 | 4.94 | 1.23 | 1.23 | 7.49 | 2.14 | 5.35 |
| **TLX2** | 4.8 | 100 | 1.16 | 1.20 | 0.00 | 5.31 | 0.00 | 5.31 |
| **HORMAD1** | 4.8 | 100 | 1.28 | 1.19 | 0.00 | 5.30 | 0.00 | 5.30 |
| **CTNNA2** | 7.7 | 94.3 | 2.50 | 3.55 | 1.54 | 12.20 | 6.92 | 5.28 |
| **GTF2H1** | 7.7 | 97.1 | 0.97 | 1.26 | 1.41 | 8.65 | 3.37 | 5.28 |
| **HCFC2** | 9.6 | 95.2 | 1.05 | 1.11 | 1.08 | 10.36 | 5.12 | 5.24 |
| **TBX6** | 4.8 | 100 | 1.09 | 1.13 | 0.00 | 5.21 | 0.00 | 5.21 |
| **PATZ1** | 5.8 | 99 | 1.08 | 1.09 | 1.02 | 6.18 | 1.01 | 5.17 |
| **BTG3** | 4.8 | 100 | 1.01 | 1.10 | 0.00 | 5.17 | 0.00 | 5.17 |
| **PDPK1** | 14.4 | 86.7 | 3.29 | 4.43 | 2.69 | 24.63 | 19.47 | 5.16 |
| **WAS** | 4.8 | 100 | 1.38 | 1.09 | 0.00 | 5.14 | 0.00 | 5.14 |
| **TBK1** | 4.8 | 100 | 1.30 | 1.08 | 0.00 | 5.13 | 0.00 | 5.13 |
| **TBX5** | 4.8 | 100 | 1.03 | 1.08 | 0.00 | 5.12 | 0.00 | 5.12 |
| **NLK** | 12.5 | 92.4 | 2.36 | 1.42 | 1.67 | 14.61 | 9.50 | 5.11 |
| **MEOX2** | 4.8 | 100 | 1.16 | 1.06 | 0.00 | 5.10 | 0.00 | 5.10 |
| **STUB1** | 8.7 | 94.3 | 3.63 | 2.70 | 1.91 | 12.53 | 7.44 | 5.08 |
| **BAG3** | 6.7 | 98.1 | 7.03 | 1.26 | 1.97 | 7.56 | 2.51 | 5.05 |
| **GMEB1** | 10.6 | 92.4 | 1.66 | 2.71 | 2.14 | 15.34 | 10.31 | 5.03 |
