# Supplementary material for: A diagnostic autoantibody signature for primary cutaneous melanoma
Source: Oncotarget. 2018 Jul 17;9(55):30539–51. doi: 10.18632/oncotarget.25669 (PMC6078131; doi:10.18632/oncotarget.25669)
Supplement: Supplementary file 4 [file oncotarget-09-30539-s004.docx]

Supplementary Table 1.3 STRING protein description for the top 139 individual biomarkers

|  | | | |  |
| --- | --- | --- | --- | --- |
|  | 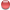 | USH1C | Usher syndrome 1C (autosomal recessive, severe); Required for normal development and maintenance of cochlear hair cell bundles. Anchoring/scaffolding protein that is a part of the functional network formed by USH1C, USH1G, CDH23 and MYO7A that mediates mechanotransduction in cochlear hair cells. Required for normal hearing (By similarity) (899 aa) |  |
|  | 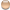 | STK10 | serine/threonine kinase 10; Serine/threonine-protein kinase involved in regulation of lymphocyte migration. Phosphorylates MSN, and possibly PLK1. Involved in regulation of lymphocyte migration by mediating phosphorylation of ERM proteins such as MSN. Acts as a negative regulator of MAP3K1/MEKK1. May also act as a cell cycle regulator by acting as a polo kinase kinase- mediates phosphorylation of PLK1 in vitro; however such data require additional evidences in vivo (968 aa) |  |
|  | 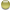 | XYLB | xylulokinase homolog (H. influenzae); Phosphorylates D-xylulose to produce D-xylulose 5- phosphate, a molecule that may play an important role in the regulation of glucose metabolism and lipogenesis (536 aa) |  |
|  | 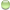 | XBP1 | X-box binding protein 1; Transcription factor essential for hepatocyte growth, the differentiation of plasma cells, the immunoglobulin secretion, and the unfolded protein response (UPR). Acts during endoplasmic reticulum stress (ER) by activating unfolded protein response (UPR) target genes via direct binding to the UPR element (UPRE). Binds DNA preferably to the CRE-like element 5’- GATGACGTG[TG]N(3)[AT]T-3’, and also to some TPA response elements (TRE). Binds to the HLA DR-alpha promoter. Binds to the Tax- responsive element (TRE) of HTLV-I (261 aa) |  |
|  | 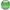 | TXN2 | thioredoxin 2; Has an anti-apoptotic function and plays an important role in the regulation of mitochondrial membrane potential. Could be involved in the resistance to anti-tumor agents. Possesses a dithiol-reducing activity (166 aa) |  |
|  | 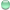 | PSME2 | proteasome (prosome, macropain) activator subunit 2 (PA28 beta); Implicated in immunoproteasome assembly and required for efficient antigen processing. The PA28 activator complex enhances the generation of class I binding peptides by altering the cleavage pattern of the proteasome (239 aa) |  |
|  | 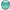 | BIRC7 | baculoviral IAP repeat containing 7; Apoptotic regulator capable of exerting proapoptotic and anti-apoptotic activities and plays crucial roles in apoptosis, cell proliferation, and cell cycle control. Its anti-apoptotic activity is mediated through the inhibition of CASP3, CASP7 and CASP9, as well as by its E3 ubiquitin-protein ligase activity. As it is a weak caspase inhibitor, its anti-apoptotic activity is thought to be due to its ability to ubiquitinate DIABLO/SMAC targeting it for degradation thereby promoting cell survival. May contribute to caspase inhibition, by blocking the a [...] (298 aa) |  |
|  | 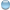 | PQBP1 | polyglutamine binding protein 1 (265 aa) |  |
|  | 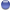 | STUB1 | STIP1 homology and U-box containing protein 1, E3 ubiquitin protein ligase; E3 ubiquitin-protein ligase which targets misfolded chaperone substrates towards proteasomal degradation. Collaborates with ATXN3 in the degradation of misfolded chaperone substrates- ATXN3 restricting the length of ubiquitin chain attached to STUB1/CHIP substrates and preventing further chain extension. Ubiquitinates NOS1 in concert with Hsp70 and Hsp40. Modulates the activity of several chaperone complexes, including Hsp70, Hsc70 and Hsp90. Mediates transfer of non-canonical short ubiquitin chains to HSPA8 th [...] (303 aa) |  |
|  | 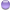 | PPP2CB | protein phosphatase 2, catalytic subunit, beta isozyme; PP2A can modulate the activity of phosphorylase B kinase casein kinase 2, mitogen-stimulated S6 kinase, and MAP-2 kinase (309 aa) |  |
|  | 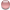 | SUPT4H1 | suppressor of Ty 4 homolog 1 (S. cerevisiae); Component of the DRB sensitivity-inducing factor complex (DSIF complex), which regulates mRNA processing and transcription elongation by RNA polymerase II. DSIF positively regulates mRNA capping by stimulating the mRNA guanylyltransferase activity of RNGTT/CAP1A. DSIF also acts cooperatively with the negative elongation factor complex (NELF complex) to enhance transcriptional pausing at sites proximal to the promoter. Transcriptional pausing may facilitate the assembly of an elongation competent RNA polymerase II complex. DSIF and NELF prom [...] (117 aa) |  |
|  | 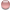 | HOXB6 | homeobox B6; Sequence-specific transcription factor which is part of a developmental regulatory system that provides cells with specific positional identities on the anterior-posterior axis (224 aa) |  |
|  | 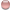 | HNF1B | HNF1 homeobox B; Transcription factor, probably binds to the inverted palindrome 5’-GTTAATNATTAAC-3’ (557 aa) |  |
|  | 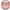 | CCND1 | cyclin D1; Regulatory component of the cyclin D1-CDK4 (DC) complex that phosphorylates and inhibits members of the retinoblastoma (RB) protein family including RB1 and regulates the cell-cycle during G(1)/S transition. Phosphorylation of RB1 allows dissociation of the transcription factor E2F from the RB/E2F complex and the subsequent transcription of E2F target genes which are responsible for the progression through the G(1) phase. Hypophosphorylates RB1 in early G(1) phase. Cyclin D-CDK4 complexes are major integrators of various mitogenenic and antimitogenic signals. Also substrate [...] (295 aa) |  |
|  | 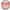 | PRDM4 | PR domain containing 4; May function as a transcription factor involved in cell differentiation (By similarity) (801 aa) |  |
|  | 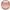 | HCFC2 | host cell factor C2 (792 aa) |  |
|  | 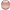 | TLX2 | T-cell leukemia homeobox 2; Transcription activator that binds DNA elements with the consensus sequence 5’-CGGTAATTGG-3’. Binds DNA via its homeobox. Required for normal cell death of enteric neurons in the gastrointestinal tract. Required for normal development of the enteric nervous system, and for proper development of normal motility of the gastrointestinal tract (By similarity) (284 aa) |  |
|  | 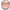 | ACVR2A | activin A receptor, type IIA; On ligand binding, forms a receptor complex consisting of two type II and two type I transmembrane serine/threonine kinases. Type II receptors phosphorylate and activate type I receptors which autophosphorylate, then bind and activate SMAD transcriptional regulators. Receptor for activin A, activin B and inhibin A (513 aa) |  |
|  | 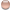 | ELK1 | ELK1, member of ETS oncogene family; Stimulates transcription. Binds to purine-rich DNA sequences. Can form a ternary complex with the serum response factor and the ETS and SRF motifs of the fos serum response element (428 aa) |  |
|  | 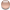 | TRAF2 | TNF receptor-associated factor 2; Regulates activation of NF-kappa-B and JNK and plays a central role in the regulation of cell survival and apoptosis. Required for normal antibody isotype switching from IgM to IgG. Has E3 ubiquitin-protein ligase activity and promotes ’Lys-63’- linked ubiquitination of target proteins, such as BIRC3, RIPK1 and TICAM1. Is an essential constituent of several E3 ubiquitin- protein ligase complexes, where it promotes the ubiquitination of target proteins by bringing them into contact with other E3 ubiquitin ligases. Regulates BIRC2 and BIRC3 protein level [...] (501 aa) |  |
|  | 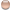 | MTERF | mitochondrial transcription termination factor; Transcription termination factor. Binds to a 28 bp region within the tRNA(Leu(uur)) gene at a position immediately adjacent to and downstream of the 16S rRNA gene; this region comprises a tridecamer sequence critical for directing accurate termination. Binds DNA along the major grove and promotes DNA bending and partial unwinding. Promotes base flipping. Probably requires one or more components for termination activity (399 aa) |  |
|  | 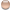 | RAC2 | ras-related C3 botulinum toxin substrate 2 (rho family, small GTP binding protein Rac2); Plasma membrane-associated small GTPase which cycles between an active GTP-bound and inactive GDP-bound state. In active state binds to a variety of effector proteins to regulate cellular responses, such as secretory processes, phagocytose of apoptotic cells and epithelial cell polarization. Augments the production of reactive oxygen species (ROS) by NADPH oxidase (192 aa) |  |
|  | 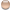 | PDGFRL | platelet-derived growth factor receptor-like (375 aa) |  |
|  | 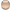 | CCNB1 | cyclin B1; Essential for the control of the cell cycle at the G2/M (mitosis) transition (433 aa) |  |
|  | 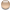 | MEOX2 | mesenchyme homeobox 2; Role in mesoderm induction and its earliest regional specification, somitogenesis, and myogenic and sclerotomal differentiation. May have a regulatory role when quiescent vascular smooth muscle cells reenter the cell cycle (By similarity) (304 aa) |  |
|  | 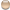 | SMAD2 | SMAD family member 2; Receptor-regulated SMAD (R-SMAD) that is an intracellular signal transducer and transcriptional modulator activated by TGF-beta (transforming growth factor) and activin type 1 receptor kinases. Binds the TRE element in the promoter region of many genes that are regulated by TGF-beta and, on formation of the SMAD2/SMAD4 complex, activates transcription. May act as a tumor suppressor in colorectal carcinoma. Positively regulates PDPK1 kinase activity by stimulating its dissociation from the 14-3-3 protein YWHAQ which acts as a negative regulator (467 aa) |  |
|  | 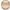 | CDKN2C | cyclin-dependent kinase inhibitor 2C (p18, inhibits CDK4); Interacts strongly with CDK6, weakly with CDK4. Inhibits cell growth and proliferation with a correlated dependence on endogenous retinoblastoma protein RB (168 aa) |  |
|  | 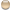 | PLD2 | phospholipase D2; May have a role in signal-induced cytoskeletal regulation and/or endocytosis (By similarity) (933 aa) |  |
|  | 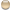 | ASB1 | ankyrin repeat and SOCS box containing 1; May play a role in testis development (By similarity). Probable substrate-recognition component of a SCF-like ECS (Elongin-Cullin-SOCS-box protein) E3 ligase complex which mediates the ubiquitination and subsequent proteasomal degradation of target proteins (335 aa) |  |
|  | 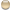 | NME5 | NME/NM23 family member 5; Does not seem to have NDK kinase activity. Confers protection from cell death by Bax and alters the cellular levels of several antioxidant enzymes including Gpx5. May play a role in spermiogenesis by increasing the ability of late-stage spermatids to eliminate reactive oxygen species (By similarity) (212 aa) |  |
|  | 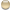 | STAP1 | signal transducing adaptor family member 1; In BCR signaling, appears to function as a docking protein acting downstream of TEC and participates in a positive feedback loop by increasing the activity of TEC (295 aa) |  |
|  | 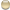 | ANXA11 | annexin A11; Binds specifically to calcyclin in a calcium-dependent manner (By similarity). Required for midbody formation and completion of the terminal phase of cytokinesis (505 aa) |  |
|  | 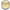 | KIF9 | kinesin family member 9 (790 aa) |  |
|  | 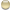 | GTF2H1 | general transcription factor IIH, polypeptide 1, 62kDa; Component of the core-TFIIH basal transcription factor involved in nucleotide excision repair (NER) of DNA and, when complexed to CAK, in RNA transcription by RNA polymerase II (548 aa) |  |
|  | 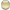 | PATZ1 | POZ (BTB) and AT hook containing zinc finger 1; Transcriptional repressor (687 aa) |  |
|  | 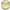 | CDK2 | cyclin-dependent kinase 2; Serine/threonine-protein kinase involved in the control of the cell cycle; essential for meiosis, but dispensable for mitosis. Phosphorylates CTNNB1, USP37, p53/TP53, NPM1, CDK7, RB1, BRCA2, MYC, NPAT, EZH2. Interacts with cyclins A, B1, B3, D, or E. Triggers duplication of centrosomes and DNA. Acts at the G1-S transition to promote the E2F transcriptional program and the initiation of DNA synthesis, and modulates G2 progression; controls the timing of entry into mitosis/meiosis by controlling the subsequent activation of cyclin B/CDK1 by phosphorylation, and [...] (298 aa) |  |
|  | 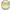 | TPM1 | tropomyosin 1 (alpha) (284 aa) |  |
|  | 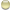 | CBFA2T3 | core-binding factor, runt domain, alpha subunit 2; translocated to, 3 (653 aa) |  |
|  | 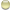 | TP53 | tumor protein p53; Acts as a tumor suppressor in many tumor types; induces growth arrest or apoptosis depending on the physiological circumstances and cell type. Involved in cell cycle regulation as a trans-activator that acts to negatively regulate cell division by controlling a set of genes required for this process. One of the activated genes is an inhibitor of cyclin-dependent kinases. Apoptosis induction seems to be mediated either by stimulation of BAX and FAS antigen expression, or by repression of Bcl-2 expression (By similarity) (393 aa) |  |
|  | 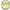 | CBLC | Cbl proto-oncogene, E3 ubiquitin protein ligase C; Regulator of EGFR mediated signal transduction (474 aa) |  |
|  | 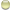 | RQCD1 | RCD1 required for cell differentiation1 homolog (S. pombe); Component of the CCR4-NOT complex which is one of the major cellular mRNA deadenylases and is linked to various cellular processes including bulk mRNA degradation, miRNA-mediated repression, translational repression during translational initiation and general transcription regulation. Additional complex functions may be a consequence of its influence on mRNA expression. Involved in down-regulation of MYB- and JUN-dependent transcription. May play a role in cell differentiation (By similarity). Can bind oligonucleotides, such a [...] (299 aa) |  |
|  | 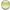 | PHIP | pleckstrin homology domain interacting protein (1821 aa) |  |
|  | 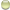 | CDK16 (PCTK1) | cyclin-dependent kinase 16; Protein kinase that plays a role in vesicle-mediated transport processes and exocytosis. Regulates GH1 release by brain neurons. Phosphorylates NSF, and thereby regulates NSF oligomerization. Required for normal spermatogenesis. Regulates neuron differentiation and dendrite development (By similarity). Plays a role in the regulation of insulin secretion in response to changes in blood glucose levels. Can phosphorylate CCNY at ’Ser- 336’ (in vitro) (570 aa) |  |
|  | 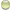 | TBX6 | T-box 6; T-box transcription factor that plays an essential role in the determination of the fate of axial stem cells- neural vs mesodermal. Acts in part by down-regulating, a specific enhancer (N1) of SOX2, to inhibit neural development. Seems to play also an essential role in left/right axis determination and acts through effects on Notch signaling around the node as well as through an effect on the morphology and motility of the nodal cilia (By similarity) (436 aa) |  |
|  | 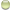 | KIT | v-kit Hardy-Zuckerman 4 feline sarcoma viral oncogene homolog; Tyrosine-protein kinase that acts as cell-surface receptor for the cytokine KITLG/SCF and plays an essential role in the regulation of cell survival and proliferation, hematopoiesis, stem cell maintenance, gametogenesis, mast cell development, migration and function, and in melanogenesis. In response to KITLG/SCF binding, KIT can activate several signaling pathways. Phosphorylates PIK3R1, PLCG1, SH2B2/APS and CBL. Activates the AKT1 signaling pathway by phosphorylation of PIK3R1, the regulatory subunit of phosphatidylinosit [...] (976 aa) |  |
|  | 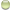 | PKNOX1 | PBX/knotted 1 homeobox 1 (436 aa) |  |
|  | 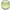 | ZBTB7B | zinc finger and BTB domain containing 7B; Transcription regulator that acts as a key regulator of lineage commitment of immature T-cell precursors. Necessary and sufficient for commitment of CD4 lineage, while its absence causes CD8 commitment. Development of immature T-cell precursors (thymocytes) to either the CD4 helper or CD8 killer T-cell lineages correlates precisely with their T-cell receptor specificity for major histocompatibility complex class II or class I molecules, respectively. Transcriptional repressor of the collagen COL1A1 and COL1A2 genes. May also function as a repre [...] (539 aa) |  |
|  | 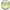 | GMEB1 | glucocorticoid modulatory element binding protein 1; Trans-acting factor that binds to glucocorticoid modulatory elements (GME) present in the TAT (tyrosine aminotransferase) promoter and increases sensitivity to low concentrations of glucocorticoids. Binds also to the transferrin receptor promoter. Essential auxiliary factor for the replication of parvoviruses (573 aa) |  |
|  | 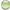 | HMGB2 | high mobility group box 2; DNA binding proteins that associates with chromatin and has the ability to bend DNA. Binds preferentially single-stranded DNA. Involved in V(D)J recombination by acting as a cofactor of the RAG complex. Acts by stimulating cleavage and RAG protein binding at the 23 bp spacer of conserved recombination signal sequences (RSS) (By similarity) (209 aa) |  |
|  | 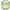 | NDRG2 | NDRG family member 2 (371 aa) |  |
|  | 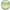 | CKB | creatine kinase, brain; Reversibly catalyzes the transfer of phosphate between ATP and various phosphogens (e.g. creatine phosphate). Creatine kinase isoenzymes play a central role in energy transduction in tissues with large, fluctuating energy demands, such as skeletal muscle, heart, brain and spermatozoa (381 aa) |  |
|  | 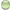 | BIRC5 | baculoviral IAP repeat containing 5 (165 aa) |  |
|  | 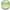 | SCAND1 | SCAN domain containing 1; May regulate transcriptional activity (179 aa) |  |
|  | 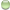 | JUNB | jun B proto-oncogene; Transcription factor involved in regulating gene activity following the primary growth factor response. Binds to the DNA sequence 5’-TGA[CG]TCA-3’ (347 aa) |  |
|  | 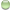 | HSFY1 | heat shock transcription factor, Y-linked 1 (401 aa) |  |
|  | 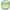 | CDC25A | cell division cycle 25 homolog A (S. pombe); Tyrosine protein phosphatase which functions as a dosage-dependent inducer of mitotic progression. Directly dephosphorylates CDK1 and stimulates its kinase activity. Also dephosphorylates CDK2 in complex with cyclin E, in vitro (524 aa) |  |
|  | 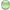 | FOXA3 | forkhead box A3; Transcription factor that is thought to act as a ’pioneer’ factor opening the compacted chromatin for other proteins through interactions with nucleosomal core histones and thereby replacing linker histones at target enhancer and/or promoter sites (By similarity). Originally described as a transcription activator for a number of liver genes such as AFP, albumin, tyrosine aminotransferase, PEPCK, etc. Interacts with the cis-acting regulatory regions of these genes. Involved in glucose homeostasis; binds to and activates transcription from the G6PC promoter. Binds to the [...] (350 aa) |  |
|  | 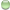 | FEN1 | flap structure-specific endonuclease 1; Structure-specific nuclease with 5’-flap endonuclease and 5’-3’ exonuclease activities involved in DNA replication and repair. During DNA replication, cleaves the 5’-overhanging flap structure that is generated by displacement synthesis when DNA polymerase encounters the 5’-end of a downstream Okazaki fragment. It enters the flap from the 5’-end and then tracks to cleave the flap base, leaving a nick for ligation. Also involved in the long patch base excision repair (LP-BER) pathway, by cleaving within the apurinic/apyrimidinic (AP) site-terminat [...] (380 aa) |  |
|  | 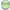 | BAD | BCL2-associated agonist of cell death; Promotes cell death. Successfully competes for the binding to Bcl-X(L), Bcl-2 and Bcl-W, thereby affecting the level of heterodimerization of these proteins with BAX. Can reverse the death repressor activity of Bcl-X(L), but not that of Bcl-2 (By similarity). Appears to act as a link between growth factor receptor signaling and the apoptotic pathways (168 aa) |  |
|  | 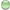 | TBX5 | T-box 5; Involved in the transcriptional regulation of genes required for mesoderm differentiation. Probably plays a role in limb pattern formation (518 aa) |  |
|  | 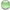 | VEGFB | vascular endothelial growth factor B; Growth factor for endothelial cells. VEGF-B167 binds heparin and neuropilin-1 whereas the binding to neuropilin-1 of VEGF-B186 is regulated by proteolysis (207 aa) |  |
|  | 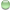 | KLK3 | kallikrein-related peptidase 3; Hydrolyzes semenogelin-1 thus leading to the liquefaction of the seminal coagulum (261 aa) |  |
|  | 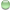 | EZH2 | enhancer of zeste homolog 2 (Drosophila) (751 aa) |  |
|  | 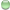 | SMARCE1 | SWI/SNF related, matrix associated, actin dependent regulator of chromatin, subfamily e, member 1; Involved in transcriptional activation and repression of select genes by chromatin remodeling (alteration of DNA-nucleosome topology). Belongs to the neural progenitors-specific chromatin remodeling complex (npBAF complex) and the neuron-specific chromatin remodeling complex (nBAF complex). During neural development a switch from a stem/progenitor to a post-mitotic chromatin remodeling mechanism occurs as neurons exit the cell cycle and become committed to their adult state. The transitio [...] (411 aa) |  |
|  | 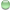 | INPP1 | inositol polyphosphate-1-phosphatase (399 aa) |  |
|  | 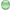 | HBG1 | hemoglobin, gamma A; Gamma chains make up the fetal hemoglobin F, in combination with alpha chains (147 aa) |  |
|  | 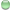 | TGIF1 | TGFB-induced factor homeobox 1; Binds to a retinoid X receptor (RXR) responsive element from the cellular retinol-binding protein II promoter (CRBPII- RXRE). Inhibits the 9-cis-retinoic acid-dependent RXR alpha transcription activation of the retinoic acid responsive element. Active transcriptional corepressor of SMAD2. Links the nodal signaling pathway to the bifurcation of the forebrain and the establishment of ventral midline structures. May participate in the transmission of nuclear signals during development and in the adult, as illustrated by the down-modulation of the RXR alpha [...] (401 aa) |  |
|  | 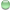 | HEXIM1 | hexamethylene bis-acetamide inducible 1; Transcriptional regulator which functions as a general RNA polymerase II transcription inhibitor. In cooperation with 7SK snRNA sequesters P-TEFb in a large inactive 7SK snRNP complex preventing RNA polymerase II phosphorylation and subsequent transcriptional elongation. May also regulate NF-kappa-B, ESR1, NR3C1 and CIITA-dependent transcriptional activity (359 aa) |  |
|  | 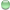 | PRKCH | protein kinase C, eta; Calcium-independent, phospholipid- and diacylglycerol (DAG)-dependent serine/threonine-protein kinase that is involved in the regulation of cell differentiation in keratinocytes and pre-B cell receptor, mediates regulation of epithelial tight junction integrity and foam cell formation, and is required for glioblastoma proliferation and apoptosis prevention in MCF-7 cells. In keratinocytes, binds and activates the tyrosine kinase FYN, which in turn blocks epidermal growth factor receptor (EGFR) signaling and leads to keratinocyte growth arrest and differentiation. [...] (683 aa) |  |
|  | 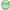 | TBK1 | TANK-binding kinase 1; Serine/threonine kinase that plays an essential role in regulating inflammatory responses to foreign agents. Following activation of toll-like receptors by viral or bacterial components, associates with TRAF3 and TANK and phosphorylates interferon regulatory factors (IRFs) IRF3 and IRF7 as well as DDX3X. This activity allows subsequent homodimerization and nuclear translocation of the IRFs leading to transcriptional activation of pro-inflammatory and antiviral genes including IFN- alpha and IFN-beta. In order to establish such an antiviral state, TBK1 form severa [...] (729 aa) |  |
|  | 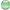 | NR1I2 | nuclear receptor subfamily 1, group I, member 2 (473 aa) |  |
|  | 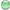 | ZFP36L1 | zinc finger protein 36, C3H type-like 1; Probable regulatory protein involved in regulating the response to growth factors (338 aa) |  |
|  | 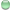 | LRRFIP2 | leucine rich repeat (in FLII) interacting protein 2; May function as activator of the canonical Wnt signaling pathway, in association with DVL3, upstream of CTNNB1/beta- catenin. Positively regulates Toll-like receptor (TLR) signaling in response to agonist probably by competing with the negative FLII regulator for MYD88-binding (721 aa) |  |
|  | 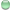 | ZNF444 | zinc finger protein 444; Transcriptional regulator. Binds to the 5’-flanking critical region of the SCARF1 promoter (327 aa) |  |
|  | 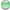 | EZR | ezrin; Probably involved in connections of major cytoskeletal structures to the plasma membrane. In epithelial cells, required for the formation of microvilli and membrane ruffles on the apical pole. Along with PLEKHG6, required for normal macropinocytosis (586 aa) |  |
|  | 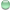 | TUBB | tubulin, beta class I (444 aa) |  |
|  | 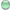 | ZNF449 | zinc finger protein 449; May be involved in transcriptional regulation (518 aa) |  |
|  | 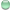 | UBE2V1 | ubiquitin-conjugating enzyme E2 variant 1 (170 aa) |  |
|  | 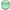 | STAT5A | signal transducer and activator of transcription 5A; Carries out a dual function- signal transduction and activation of transcription. Mediates cellular responses to the cytokine KITLG/SCF and other growth factors. Mediates cellular responses to ERBB4. May mediate cellular responses to activated FGFR1, FGFR2, FGFR3 and FGFR4. Binds to the GAS element and activates PRL-induced transcription. Regulates the expression of milk proteins during lactation (794 aa) |  |
|  | 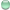 | PDPK1 | 3-phosphoinositide dependent protein kinase-1; Serine/threonine kinase which acts as a master kinase, phosphorylating and activating a subgroup of the AGC family of protein kinases. Its targets include- protein kinase B (PKB/AKT1, PKB/AKT2, PKB/AKT3), p70 ribosomal protein S6 kinase (RPS6KB1), p90 ribosomal protein S6 kinase (RPS6KA1, RPS6KA2 and RPS6KA3), cyclic AMP-dependent protein kinase (PRKACA), protein kinase C (PRKCD and PRKCZ), serum and glucocorticoid-inducible kinase (SGK1, SGK2 and SGK3), p21-activated kinase-1 (PAK1), protein kinase PKN (PKN1 and PKN2). Plays a central rol [...] (556 aa) |  |
|  | 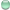 | BTG3 | BTG family, member 3; Overexpression impairs serum-induced cell cycle progression from the G0/G1 to S phase (296 aa) |  |
|  | 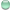 | NFYA | nuclear transcription factor Y, alpha; Stimulates the transcription of various genes by recognizing and binding to a CCAAT motif in promoters, for example in type 1 collagen, albumin and beta-actin genes (347 aa) |  |
|  | 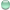 | MEF2A | myocyte enhancer factor 2A (499 aa) |  |
|  | 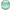 | AK2 | adenylate kinase 2 (239 aa) |  |
|  | 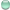 | CREB5 | cAMP responsive element binding protein 5 (508 aa) |  |
|  | 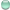 | MAFG | v-maf musculoaponeurotic fibrosarcoma oncogene homolog G (avian); Since they lack a putative transactivation domain, the small Mafs behave as transcriptional repressors when they dimerize among themselves. However, they seem to serve as transcriptional activators by dimerizing with other (usually larger) basic-zipper proteins and recruiting them to specific DNA-binding sites. Small Maf proteins heterodimerize with Fos and may act as competitive repressors of the NF-E2 transcription factor. Transcription factor, component of erythroid-specific transcription factor NF- E2. Activates glob [...] (162 aa) |  |
|  | 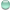 | RAD23B | RAD23 homolog B (S. cerevisiae); Multiubiquitin chain receptor involved in modulation of proteasomal degradation. Binds to polyubiquitin chains. Proposed to be capable to bind simultaneously to the 26S proteasome and to polyubiquitinated substrates and to deliver ubiquitinated proteins to the proteasome. May play a role in endoplasmic reticulum- associated degradation (ERAD) of misfolded glycoproteins by association with PNGase and delivering deglycosylated proteins to the proteasome (409 aa) |  |
|  | 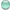 | STAT4 | signal transducer and activator of transcription 4; Carries out a dual function- signal transduction and activation of transcription. Involved in IL12 signaling (748 aa) |  |
|  | 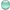 | MAX | MYC associated factor X; Transcription regulator. Forms a sequence-specific DNA- binding protein complex with MYC or MAD which recognizes the core sequence 5’-CAC[GA]TG-3’. The MYC-MAX complex is a transcriptional activator, whereas the MAD-MAX complex is a repressor. May repress transcription via the recruitment of a chromatin remodeling complex containing H3 ’Lys-9’ histone methyltransferase activity (160 aa) |  |
|  | 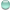 | MSN | moesin; Probably involved in connections of major cytoskeletal structures to the plasma membrane. May inhibit herpes simplex virus 1 infection at an early stage (577 aa) |  |
|  | 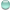 | MAPK8 | mitogen-activated protein kinase 8; Serine/threonine-protein kinase involved in various processes such as cell proliferation, differentiation, migration, transformation and programmed cell death. Extracellular stimuli such as proinflammatory cytokines or physical stress stimulate the stress-activated protein kinase/c-Jun N-terminal kinase (SAP/JNK) signaling pathway. In this cascade, two dual specificity kinases MAP2K4/MKK4 and MAP2K7/MKK7 phosphorylate and activate MAPK8/JNK1. In turn, MAPK8/JNK1 phosphorylates a number of transcription factors, primarily components of AP-1 such as JU [...] (427 aa) |  |
|  | 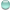 | DLX1 | distal-less homeobox 1; Likely to play a regulatory role in the development of the ventral forebrain. May play a role in craniofacial patterning and morphogenesis and may be involved in the early development of diencephalic subdivisions (By similarity) (255 aa) |  |
|  | 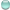 | HORMAD1 | HORMA domain containing 1 (394 aa) |  |
|  | 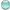 | DSTYK | dual serine/threonine and tyrosine protein kinase (929 aa) |  |
|  | 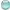 | IFI16 | interferon, gamma-inducible protein 16 (729 aa) |  |
|  | 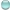 | PYGO2 | pygopus homolog 2 (Drosophila); Involved in signal transduction through the Wnt pathway (406 aa) |  |
|  | 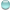 | BAG3 | BCL2-associated athanogene 3; Inhibits the chaperone activity of HSP70/HSC70 by promoting substrate release. Has anti-apoptotic activity (575 aa) |  |
|  | 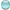 | CASP7 | caspase 7, apoptosis-related cysteine peptidase (336 aa) |  |
|  | 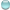 | TTF2 | transcription termination factor, RNA polymerase II; DsDNA-dependent ATPase which acts as a transcription termination factor by coupling ATP hydrolysis with removal of RNA polymerase II from the DNA template. May contribute to mitotic transcription repression. May also be involved in pre-mRNA splicing (1162 aa) |  |
|  | 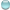 | DR1 | down-regulator of transcription 1, TBP-binding (negative cofactor 2); The association of the DR1/DRAP1 heterodimer with TBP results in a functional repression of both activated and basal transcription of class II genes. This interaction precludes the formation of a transcription-competent complex by inhibiting the association of TFIIA and/or TFIIB with TBP. Can bind to DNA on its own. Component of the ATAC complex, a complex with histone acetyltransferase activity on histones H3 and H4 (176 aa) |  |
|  |  | FMR1NB | fragile X mental retardation 1 neighbor (255 aa) |  |
|  |  | CEP55 | centrosomal protein 55kDa; Plays a role in mitotic exit and cytokinesis. Not required for microtubule nucleation. Recruits PDCD6IP and TSG101 to midbody during cytokinesis (464 aa) |  |
|  |  | FAF1 | Fas (TNFRSF6) associated factor 1; Potentiates but cannot initiate FAS-induced apoptosis (650 aa) |  |
|  |  | MUTYH | mutY homolog (E. coli); Involved in oxidative DNA damage repair. Initiates repair of A*oxoG to C*G by removing the inappropriately paired adenine base from the DNA backbone. Possesses both adenine and 2- OH-A DNA glycosylase activities (546 aa) |  |
|  |  | HEYL | hairy/enhancer-of-split related with YRPW motif-like; Downstream effector of Notch signaling which may be required for cardiovascular development (By similarity). Transcriptional repressor which binds preferentially to the canonical E box sequence 5’-CACGTG-3’ (By similarity). Represses transcription by the cardiac transcriptional activators GATA4 and GATA6 (328 aa) |  |
|  |  | PTPN20A | protein tyrosine phosphatase, non-receptor type 20A (420 aa) |  |
|  |  | RING1 | ring finger protein 1 (406 aa) |  |
|  |  | HSPA1A | heat shock 70kDa protein 1A (641 aa) |  |
|  |  | WAS | Wiskott-Aldrich syndrome; Effector protein for Rho-type GTPases. Regulates actin filament reorganization via its interaction with the Arp2/3 complex. Important for efficient actin polymerization. Possible regulator of lymphocyte and platelet function. Mediates actin filament reorganization and the formation of actin pedestals upon infection by pathogenic bacteria (502 aa) |  |
|  |  | HRH2 | histamine receptor H2; The H2 subclass of histamine receptors mediates gastric acid secretion. Also appears to regulate gastrointestinal motility and intestinal secretion. Possible role in regulating cell growth and differentiation. The activity of this receptor is mediated by G proteins which activate adenylyl cyclase and, through a separate G protein-dependent mechanism, the phosphoinositide/protein kinase (PKC) signaling pathway (By similarity) (397 aa) |  |
|  |  | KLF12 | Kruppel-like factor 12; Confers strong transcriptional repression to the AP-2- alpha gene. Binds to a regulatory element (A32) in the AP-2-alpha gene promoter (402 aa) |  |
|  |  | SLCO6A1 | solute carrier organic anion transporter family, member 6A1 (719 aa) |  |
|  |  | IRF4 | interferon regulatory factor 4; Transcriptional activator. Binds to the interferon- stimulated response element (ISRE) of the MHC class I promoter. Binds the immunoglobulin lambda light chain enhancer, together with PU.1. Probably plays a role in ISRE-targeted signal transduction mechanisms specific to lymphoid cells. Involved in CD8(+) dendritic cell differentiation by forming a complex with the BATF-JUNB heterodimer in immune cells, leading to recognition of AICE sequence (5’-TGAnTCA/GAAA-3’), an immune-specific regulatory element, followed by cooperative binding of BATF and IRF4 and [...] (451 aa) |  |
|  |  | CWC27 (SDCCAG10) | CWC27 spliceosome-associated protein homolog (S. cerevisiae); PPIases accelerate the folding of proteins (By similarity) (472 aa) |  |
|  |  | SLC25A6 | solute carrier family 25 (mitochondrial carrier; adenine nucleotide translocator), member 6; Catalyzes the exchange of cytoplasmic ADP with mitochondrial ATP across the mitochondrial inner membrane. May participate in the formation of the permeability transition pore complex (PTPC) responsible for the release of mitochondrial products that triggers apoptosis (298 aa) |  |
|  |  | MLANA | melan-A; Involved in melanosome biogenesis by ensuring the stability of GPR143. Plays a vital role in the expression, stability, trafficking, and processing of melanocyte protein PMEL, which is critical to the formation of stage II melanosomes (118 aa) |  |
|  |  | CHEK2 | checkpoint kinase 2 (586 aa) |  |
|  |  | SERPINB5 | serpin peptidase inhibitor, clade B (ovalbumin), member 5; Tumor suppressor. It blocks the growth, invasion, and metastatic properties of mammary tumors. As it does not undergo the S (stressed) to R (relaxed) conformational transition characteristic of active serpins, it exhibits no serine protease inhibitory activity (375 aa) |  |
|  |  | STK38L | serine/threonine kinase 38 like; Involved in the regulation of structural processes in differentiating and mature neuronal cells (By similarity) (464 aa) |  |
|  |  | ZNF169 | zinc finger protein 169; May be involved in transcriptional regulation (603 aa) |  |
|  |  | EXT2 | exostosin 2; Glycosyltransferase required for the biosynthesis of heparan-sulfate. The EXT1/EXT2 complex possesses substantially higher glycosyltransferase activity than EXT1 or EXT2 alone. Appears to be a tumor suppressor (751 aa) |  |
|  |  | GTF2A2 | general transcription factor IIA, 2, 12kDa; TFIIA is a component of the transcription machinery of RNA polymerase II and plays an important role in transcriptional activation. TFIIA in a complex with TBP mediates transcriptional activity (109 aa) |  |
|  |  | RPL32 | ribosomal protein L32 (135 aa) |  |
|  |  | NFE2L2 | nuclear factor (erythroid-derived 2)-like 2; Transcription activator that binds to antioxidant response (ARE) elements in the promoter regions of target genes. Important for the coordinated up-regulation of genes in response to oxidative stress. May be involved in the transcriptional activation of genes of the beta-globin cluster by mediating enhancer activity of hypersensitive site 2 of the beta-globin locus control region (605 aa) |  |
|  |  | CCDC33 | coiled-coil domain containing 33 (755 aa) |  |
|  |  | NLK | nemo-like kinase; Serine/threonine-protein kinase that regulates a number of transcription factors with key roles in cell fate determination. Positive effector of the non-canonical Wnt signaling pathway, acting downstream of WNT5A, MAP3K7/TAK1 and HIPK2. Activation of this pathway causes binding to and phosphorylation of the histone methyltransferase SETDB1. The NLK- SETDB1 complex subsequently interacts with PPARG, leading to methylation of PPARG target promoters at histone H3K9 and transcriptional silencing. The resulting loss of PPARG target gene transcription inhibits adipogenesis [...] (527 aa) |  |
|  |  | DLX3 | distal-less homeobox 3; Likely to play a regulatory role in the development of the ventral forebrain. May play a role in craniofacial patterning and morphogenesis (287 aa) |  |
|  |  | SCFD1 | sec1 family domain containing 1; Plays a role in SNARE-pin assembly and Golgi-to-ER retrograde transport via its interaction with COG4. Involved in vesicular transport between the endoplasmic reticulum and the Golgi (By similarity) (642 aa) |  |
|  |  | EEF1D | eukaryotic translation elongation factor 1 delta (guanine nucleotide exchange protein); Isoform 1- EF-1-beta and EF-1-delta stimulate the exchange of GDP bound to EF-1-alpha to GTP, regenerating EF-1- alpha for another round of transfer of aminoacyl-tRNAs to the ribosome (647 aa) |  |
|  |  | MECP2 | methyl CpG binding protein 2 (Rett syndrome); Chromosomal protein that binds to methylated DNA. It can bind specifically to a single methyl-CpG pair. It is not influenced by sequences flanking the methyl-CpGs. Mediates transcriptional repression through interaction with histone deacetylase and the corepressor SIN3A (498 aa) |  |
|  |  | PBX1 | pre-B-cell leukemia homeobox 1; Binds the sequence 5’-ATCAATCAA-3’. Acts as a transcriptional activator of PF4 in complex with MEIS1. Converted into a potent transcriptional activator by the (1;19) translocation. May have a role in steroidogenesis and, subsequently, sexual development and differentiation. Isoform PBX1b as part of a PDX1-PBX1b-MEIS2b complex in pancreatic acinar cells is involved in the transcriptional activation of the ELA1 enhancer; the complex binds to the enhancer B element and cooperates with the transcription factor 1 complex (PTF1) bound to the enhancer A element [...] (430 aa) |  |
|  |  | PAPSS2 | 3’-phosphoadenosine 5’-phosphosulfate synthase 2; Bifunctional enzyme with both ATP sulfurylase and APS kinase activity, which mediates two steps in the sulfate activation pathway. The first step is the transfer of a sulfate group to ATP to yield adenosine 5’-phosphosulfate (APS), and the second step is the transfer of a phosphate group from ATP to APS yielding 3’-phosphoadenylylsulfate (PAPS- activated sulfate donor used by sulfotransferase). In mammals, PAPS is the sole source of sulfate; APS appears to be only an intermediate in the sulfate- activation pathway. May have a important [...] (619 aa) |  |
|  |  | IMPA1 | inositol(myo)-1(or 4)-monophosphatase 1; Responsible for the provision of inositol required for synthesis of phosphatidylinositol and polyphosphoinositides and has been implicated as the pharmacological target for lithium action in brain. Can use myo-inositol monophosphates, myo-inositol 1,3-diphosphate, myo-inositol 1,4-diphosphate, scyllo-inositol- phosphate, glucose-1-phosphate, glucose-6-phosphate, fructose-1- phosphate, beta-glycerophosphate, and 2’-AMP as substrates (336 aa) |  |
|  |  | STMN1 | stathmin 1; Involved in the regulation of the microtubule (MT) filament system by destabilizing microtubules. Prevents assembly and promotes disassembly of microtubules. Phosphorylation at Ser- 16 may be required for axon formation during neurogenesis. Involved in the control of the learned and innate fear (By similarity) (174 aa) |  |
|  |  | CTNNA2 | catenin (cadherin-associated protein), alpha 2 (905 aa) |  |
|  |  | CDK18 | cyclin-dependent kinase 18; May play a role in signal transduction cascades in terminally differentiated cells (504 aa) |  |
|  |  | C1orf216 | chromosome 1 open reading frame 216 (229 aa) |  |
|  |  | FOXR2 | forkhead box R2 (311 aa) |  |
|  |  | DPF2 | D4, zinc and double PHD fingers family 2; May be a transcription factor required for the apoptosis response following survival factor withdrawal from myeloid cells. Might also have a role in the development and maturation of lymphoid cells (391 aa) |  |
