# Supplementary material for: A diagnostic autoantibody signature for primary cutaneous melanoma
Source: Oncotarget. 2018 Jul 17;9(55):30539–51. doi: 10.18632/oncotarget.25669 (PMC6078131; doi:10.18632/oncotarget.25669)
Supplement: Supplementary file 5 [file oncotarget-09-30539-s005.docx]

| **#pathway ID** | **pathway description** | **observed gene count** | **false discovery rate** | **matching proteins in your network (labels)** |
| --- | --- | --- | --- | --- |
| GO.0009987 | cellular process | 92 | 0.0327 | ACVR2A,AK2,ANXA11,ASB1,BIRC5,BIRC7,CASP7,CBFA2T3,CBLC,CCNB1,CDC25A,CDK16,CDK18,CDK2,CDKN2C,CEP55,CHEK2,CKB,CREB5,CTNNA2,CWC27,DLX1,DLX3,DPF2,DR1,DSTYK,EEF1D,EXT2,EZR,FAF1,FEN1,FOXR2,GMEB1,GTF2A2,GTF2H1,HCFC2,HEXIM1,HEYL,HMGB2,HNF1B,HORMAD1,HOXB6,HSFY1,IFI16,INPP1,KIF9,KLF12,KLK3,LRRFIP2,MAFG,MAX,MECP2,MEF2A,MEOX2,MSN,MTERF,MUTYH,NDRG2,NFE2L2,NFYA,NR1I2,PAPSS2,PATZ1,PDGFRL,PDPK1,PHIP,PSME2,PTPN20A,PYGO2,SCAND1,SLC25A6,SMAD2,SMARCE1,STAP1,STAT5A,STK10,STK38L,STUB1,SUPT4H1,TBX5,TBX6,TLX2,TP53,TPM1,TRAF2,TTF2,XYLB,ZBTB7B,ZFP36L1,ZNF169,ZNF444,ZNF449 |
| GO.0044237 | cellular metabolic process | 88 | 1.37E-08 | AK2,ASB1,BAD,BAG3,BIRC5,BIRC7,CBFA2T3,CBLC,CCND1,CDC25A,CDK16,CDK18,CDK2,CHEK2,CKB,CREB5,CWC27,DLX3,DPF2,DR1,DSTYK,EEF1D,ELK1,EXT2,EZR,FAF1,FEN1,FOXA3,FOXR2,GMEB1,GTF2A2,GTF2H1,HEXIM1,HEYL,HMGB2,HNF1B,HORMAD1,HOXB6,HSFY1,IFI16,INPP1,KLF12,KLK3,MAFG,MAPK8,MAX,MECP2,MEF2A,MEOX2,MTERF,MUTYH,NFYA,NR1I2,PAPSS2,PATZ1,PBX1,PDGFRL,PDPK1,PQBP1,PRDM4,PSME2,PTPN20A,RQCD1,SCAND1,SLC25A6,SMAD2,STAT4,STAT5A,STK10,STK38L,STUB1,SUPT4H1,TBK1,TBX6,TGIF1,TLX2,TP53,TRAF2,TTF2,TXN2,VEGFB,XBP1,XYLB,ZBTB7B,ZFP36L1,ZNF169,ZNF444,ZNF449 |
| GO.0044238 | primary metabolic process | 87 | 9.00E-08 | AK2,ASB1,BAD,BAG3,BIRC5,BIRC7,CASP7,CBFA2T3,CBLC,CCND1,CDC25A,CDK16,CDK18,CDK2,CHEK2,CKB,CREB5,CWC27,DLX3,DPF2,DR1,DSTYK,EEF1D,ELK1,EXT2,FAF1,FEN1,FOXA3,FOXR2,GMEB1,GTF2A2,GTF2H1,HEXIM1,HEYL,HMGB2,HNF1B,HORMAD1,HOXB6,HSFY1,IFI16,INPP1,KLF12,MAFG,MAPK8,MAX,MECP2,MEF2A,MEOX2,MTERF,MUTYH,NFYA,NR1I2,PAPSS2,PATZ1,PBX1,PDGFRL,PDPK1,PLD2,PQBP1,PRDM4,PSME2,PTPN20A,RQCD1,SCAND1,SLC25A6,SMAD2,STAT4,STAT5A,STK10,STK38L,STUB1,SUPT4H1,TBK1,TBX6,TGIF1,TLX2,TP53,TTF2,TXN2,VEGFB,XBP1,XYLB,ZBTB7B,ZFP36L1,ZNF169,ZNF444,ZNF449 |
| GO.0008152 | metabolic process | 87 | 3.17E-05 | AK2,ASB1,BAD,BAG3,BIRC5,BIRC7,CASP7,CBFA2T3,CBLC,CCND1,CDC25A,CDK16,CDK18,CDK2,CHEK2,CKB,CREB5,CWC27,DLX3,DPF2,DR1,DSTYK,EEF1D,ELK1,EXT2,EZR,FAF1,FEN1,FOXA3,FOXR2,GMEB1,GTF2A2,GTF2H1,HEXIM1,HEYL,HMGB2,HNF1B,HORMAD1,HOXB6,HSFY1,IFI16,INPP1,KIF9,KLF12,MAFG,MAPK8,MAX,MECP2,MEF2A,MEOX2,MTERF,MUTYH,NFYA,NR1I2,PAPSS2,PATZ1,PBX1,PDGFRL,PDPK1,PQBP1,PRDM4,PSME2,PTPN20A,RAC2,RQCD1,SCAND1,SLC25A6,SMAD2,SMARCE1,STAT4,STAT5A,STK10,STK38L,STUB1,SUPT4H1,TBK1,TBX6,TGIF1,TLX2,TP53,VEGFB,XBP1,XYLB,ZBTB7B,ZNF169,ZNF444,ZNF449 |
| GO.0065007 | biological regulation | 86 | 0.000155 | ACVR2A,BAD,BIRC5,BIRC7,BTG3,CASP7,CBLC,CCNB1,CCND1,CDC25A,CDK16,CDK18,CDK2,CDKN2C,CHEK2,CKB,CREB5,CTNNA2,DLX1,DPF2,DR1,DSTYK,EEF1D,EXT2,FAF1,FEN1,FOXA3,FOXR2,GTF2A2,GTF2H1,HBG1,HCFC2,HEXIM1,HMGB2,HORMAD1,HOXB6,HSFY1,IFI16,IMPA1,INPP1,KIF9,KLK3,LRRFIP2,MAPK8,MEF2A,MEOX2,MSN,MTERF,NFE2L2,NLK,NME5,NR1I2,PAPSS2,PDGFRL,PDPK1,PSME2,PYGO2,RAC2,RAD23B,SCAND1,SCFD1,SLC25A6,SMAD2,SMARCE1,STAP1,STAT5A,STK10,STK38L,STUB1,TBK1,TBX5,TBX6,TLX2,TP53,TPM1,TRAF2,TTF2,TXN2,UBE2V1,USH1C,VEGFB,WAS,ZBTB7B,ZNF169,ZNF444,ZNF449 |
| GO.0071704 | organic substance metabolic process | 85 | 2.52E-06 | AK2,ASB1,BAD,BAG3,BIRC5,BIRC7,CASP7,CBFA2T3,CBLC,CCND1,CDC25A,CDK16,CDK18,CDK2,CHEK2,CKB,CREB5,CWC27,DLX3,DPF2,DR1,DSTYK,EEF1D,ELK1,EXT2,EZR,FAF1,FEN1,FOXA3,FOXR2,GMEB1,GTF2A2,GTF2H1,HEXIM1,HEYL,HMGB2,HNF1B,HORMAD1,HOXB6,HSFY1,IFI16,KLF12,MAFG,MAPK8,MAX,MECP2,MEF2A,MEOX2,MTERF,MUTYH,NFYA,NR1I2,PAPSS2,PATZ1,PBX1,PDGFRL,PDPK1,PQBP1,PRDM4,PSME2,PTPN20A,RQCD1,SCAND1,SLC25A6,SMAD2,STAT4,STAT5A,STK10,STK38L,STUB1,SUPT4H1,TBK1,TBX6,TGIF1,TLX2,TP53,TTF2,TXN2,VEGFB,XBP1,XYLB,ZBTB7B,ZNF169,ZNF444,ZNF449 |
| GO.0043170 | macromolecule metabolic process | 83 | 2.33E-09 | ASB1,BAD,BAG3,BIRC5,BIRC7,CASP7,CBFA2T3,CBLC,CCND1,CDC25A,CDK16,CDK18,CDK2,CHEK2,CREB5,CWC27,DLX3,DPF2,DR1,DSTYK,EEF1D,ELK1,EXT2,EZR,FAF1,FEN1,FOXA3,FOXR2,GMEB1,GTF2A2,GTF2H1,HEXIM1,HEYL,HMGB2,HNF1B,HORMAD1,HOXB6,HSFY1,IFI16,KLF12,MAFG,MAPK8,MAX,MECP2,MEF2A,MEOX2,MTERF,MUTYH,NFYA,NR1I2,PAPSS2,PATZ1,PBX1,PDGFRL,PDPK1,PLD2,PQBP1,PRDM4,PSME2,PTPN20A,RQCD1,SCAND1,SLC25A6,SMAD2,STAT4,STAT5A,STK10,STK38L,STUB1,SUPT4H1,TBK1,TBX6,TGIF1,TLX2,TP53,TTF2,TXN2,VEGFB,XBP1,ZBTB7B,ZNF169,ZNF444,ZNF449 |
| GO.0044260 | cellular macromolecule metabolic process | 82 | 1.36E-10 | ASB1,BAG3,BIRC5,BIRC7,CBFA2T3,CBLC,CCND1,CDC25A,CDK16,CDK18,CDK2,CHEK2,CREB5,CWC27,DLX3,DPF2,DR1,DSTYK,EEF1D,ELK1,EXT2,EZR,FAF1,FEN1,FOXA3,FOXR2,GMEB1,GTF2A2,GTF2H1,HEXIM1,HEYL,HMGB2,HNF1B,HOXB6,HSFY1,IFI16,KLF12,KLK3,MAFG,MAPK8,MAX,MECP2,MEF2A,MEOX2,MTERF,MUTYH,NFYA,NR1I2,PATZ1,PBX1,PDGFRL,PDPK1,PLD2,PQBP1,PRDM4,PSME2,PTPN20A,RQCD1,SCAND1,SLC25A6,SMAD2,STAT4,STAT5A,STK10,STK38L,STUB1,SUPT4H1,TBK1,TBX6,TGIF1,TLX2,TP53,TRAF2,TTF2,TXN2,VEGFB,XBP1,ZBTB7B,ZFP36L1,ZNF169,ZNF444,ZNF449 |
| GO.0050789 | regulation of biological process | 81 | 0.00124 | ACVR2A,BAD,BIRC5,BIRC7,BTG3,CASP7,CBLC,CCNB1,CCND1,CDC25A,CDK16,CDK18,CDK2,CDKN2C,CHEK2,CREB5,CTNNA2,DLX1,DPF2,DR1,DSTYK,EEF1D,EXT2,FAF1,FOXA3,FOXR2,GTF2A2,GTF2H1,HCFC2,HEXIM1,HMGB2,HORMAD1,HOXB6,HSFY1,IFI16,IMPA1,INPP1,KIF9,KLK3,LRRFIP2,MAPK8,MEF2A,MEOX2,MSN,MTERF,NFE2L2,NLK,NME5,NR1I2,PDGFRL,PDPK1,PSME2,PYGO2,RAC2,RAD23B,SCAND1,SCFD1,SLC25A6,SMAD2,SMARCE1,STAP1,STAT5A,STK10,STK38L,STUB1,TBK1,TBX5,TBX6,TLX2,TP53,TPM1,TRAF2,TTF2,TXN2,UBE2V1,VEGFB,WAS,ZBTB7B,ZNF169,ZNF444,ZNF449 |
| GO.0050794 | regulation of cellular process | 80 | 0.000592 | ACVR2A,BAD,BIRC5,BIRC7,BTG3,CASP7,CBLC,CCNB1,CCND1,CDC25A,CDK16,CDK18,CDK2,CDKN2C,CHEK2,CREB5,CTNNA2,DLX1,DPF2,DR1,DSTYK,EEF1D,EXT2,FAF1,FOXA3,FOXR2,GTF2A2,GTF2H1,HCFC2,HEXIM1,HMGB2,HORMAD1,HOXB6,HSFY1,IFI16,IMPA1,INPP1,KIF9,KLK3,LRRFIP2,MAPK8,MEF2A,MEOX2,MSN,MTERF,NFE2L2,NLK,NME5,NR1I2,PDGFRL,PDPK1,PSME2,PYGO2,RAC2,RAD23B,SCAND1,SCFD1,SLC25A6,SMAD2,SMARCE1,STAP1,STK10,STK38L,STUB1,TBK1,TBX5,TBX6,TLX2,TP53,TPM1,TRAF2,TTF2,TXN2,UBE2V1,VEGFB,WAS,ZBTB7B,ZNF169,ZNF444,ZNF449 |
| GO.0019222 | regulation of metabolic process | 75 | 9.21E-09 | ACVR2A,ASB1,BAD,BIRC5,BIRC7,CASP7,CBLC,CCND1,CDC25A,CDKN2C,CHEK2,CREB5,DLX1,DPF2,DR1,DSTYK,EEF1D,ELK1,EZR,FAF1,FOXA3,FOXR2,GTF2A2,GTF2H1,HCFC2,HEXIM1,HMGB2,HOXB6,HRH2,HSFY1,IFI16,IRF4,KIT,MAPK8,MEF2A,MEOX2,MSN,MTERF,NDRG2,NFE2L2,NLK,NR1I2,PDPK1,PPP2CB,PQBP1,PRDM4,PRKCH,PSME2,PYGO2,RAC2,RAD23B,RQCD1,SCAND1,SCFD1,SERPINB5,SMAD2,SMARCE1,STAP1,STK10,TBK1,TBX5,TBX6,TGIF1,TLX2,TP53,TPM1,TRAF2,TTF2,UBE2V1,VEGFB,WAS,ZBTB7B,ZNF169,ZNF444,ZNF449 |
| GO.0031323 | regulation of cellular metabolic process | 73 | 3.22E-10 | ACVR2A,ASB1,BAD,BIRC5,BIRC7,CASP7,CBLC,CCND1,CDC25A,CDKN2C,CHEK2,CREB5,DLX1,DPF2,DR1,DSTYK,EEF1D,ELK1,EZR,FAF1,FOXA3,FOXR2,GTF2A2,GTF2H1,HCFC2,HEXIM1,HMGB2,HOXB6,HRH2,HSFY1,IFI16,IRF4,KIT,MAFG,MAPK8,MEF2A,MEOX2,MSN,MTERF,NDRG2,NFE2L2,NLK,NR1I2,PDPK1,PQBP1,PRDM4,PRKCH,PSME2,PYGO2,RAC2,RAD23B,RQCD1,SCAND1,SCFD1,SERPINB5,SMAD2,SMARCE1,STAP1,STK10,TBK1,TBX5,TBX6,TGIF1,TLX2,TP53,TRAF2,TTF2,UBE2V1,VEGFB,ZBTB7B,ZNF169,ZNF444,ZNF449 |
| GO.0080090 | regulation of primary metabolic process | 72 | 1.96E-10 | ACVR2A,ASB1,BAD,BIRC5,BIRC7,CASP7,CBLC,CCND1,CDC25A,CDKN2C,CHEK2,CREB5,DLX1,DPF2,DR1,DSTYK,EEF1D,ELK1,EZR,FAF1,FOXA3,FOXR2,GTF2A2,GTF2H1,HCFC2,HEXIM1,HMGB2,HOXB6,HRH2,HSFY1,IFI16,IRF4,KIT,MAFG,MAPK8,MEF2A,MEOX2,MSN,MTERF,NDRG2,NFE2L2,NLK,NR1I2,PDPK1,PQBP1,PRDM4,PRKCH,PSME2,PYGO2,RAC2,RAD23B,RQCD1,SCAND1,SERPINB5,SMAD2,SMARCE1,STAP1,STK10,TBK1,TBX5,TBX6,TGIF1,TLX2,TP53,TRAF2,TTF2,UBE2V1,VEGFB,ZBTB7B,ZNF169,ZNF444,ZNF449 |
| GO.0060255 | regulation of macromolecule metabolic process | 71 | 3.36E-10 | ACVR2A,ASB1,BAD,BIRC5,BIRC7,CASP7,CBLC,CCND1,CDC25A,CDKN2C,CHEK2,CREB5,DLX1,DPF2,DR1,DSTYK,EEF1D,ELK1,EZR,FAF1,FOXA3,FOXR2,GTF2A2,GTF2H1,HCFC2,HEXIM1,HMGB2,HOXB6,HSFY1,IFI16,IRF4,KIT,MAPK8,MEF2A,MEOX2,MSN,MTERF,NDRG2,NFE2L2,NLK,NR1I2,PDPK1,PPP2CB,PQBP1,PRDM4,PRKCH,PSME2,PYGO2,RAC2,RAD23B,RQCD1,SCAND1,SERPINB5,SMAD2,SMARCE1,STAP1,STK10,TBK1,TBX5,TBX6,TGIF1,TLX2,TP53,TRAF2,TTF2,UBE2V1,VEGFB,ZBTB7B,ZNF169,ZNF444,ZNF449 |
| GO.0006807 | nitrogen compound metabolic process | 71 | 2.10E-09 | AK2,BAD,BIRC5,CBFA2T3,CCND1,CDC25A,CDK2,CHEK2,CKB,CREB5,CWC27,DLX3,DPF2,DR1,EEF1D,ELK1,EXT2,EZH2,FEN1,FOXA3,FOXR2,GMEB1,GTF2A2,GTF2H1,HEXIM1,HEYL,HMGB2,HNF1B,HORMAD1,HOXB6,HSFY1,IFI16,IRF4,KIT,KLF12,MAFG,MAX,MECP2,MEF2A,MEOX2,MTERF,MUTYH,NFYA,NLK,NR1I2,PAPSS2,PATZ1,PBX1,PDPK1,PQBP1,PRDM4,PSME2,RAD23B,RQCD1,SCAND1,SMAD2,STAT4,STAT5A,STUB1,SUPT4H1,TBX6,TGIF1,TLX2,TP53,TTF2,XBP1,ZBTB7B,ZFP36L1,ZNF169,ZNF444,ZNF449 |
| GO.0034641 | cellular nitrogen compound metabolic process | 69 | 1.20E-09 | AK2,BAD,BIRC5,CBFA2T3,CCND1,CDC25A,CDK2,CHEK2,CKB,CREB5,CWC27,DLX3,DPF2,DR1,EEF1D,ELK1,EZH2,FEN1,FOXA3,FOXR2,GMEB1,GTF2A2,GTF2H1,HEXIM1,HEYL,HMGB2,HNF1B,HORMAD1,HOXB6,HSFY1,IFI16,IRF4,KLF12,MAFG,MAX,MECP2,MEF2A,MEOX2,MTERF,MUTYH,NFYA,NLK,NR1I2,PAPSS2,PATZ1,PBX1,PDPK1,PQBP1,PRDM4,PSME2,RAD23B,RQCD1,SCAND1,SMAD2,STAT4,STAT5A,STUB1,SUPT4H1,TBX6,TGIF1,TLX2,TP53,TTF2,XBP1,ZBTB7B,ZFP36L1,ZNF169,ZNF444,ZNF449 |
| GO.0006139 | nucleobase-containing compound metabolic process | 68 | 1.34E-11 | AK2,BAD,BIRC5,CBFA2T3,CCND1,CDC25A,CDK2,CHEK2,CREB5,CWC27,DLX3,DPF2,DR1,EEF1D,ELK1,EZH2,FEN1,FOXA3,FOXR2,GMEB1,GTF2A2,GTF2H1,HEXIM1,HEYL,HMGB2,HNF1B,HORMAD1,HOXB6,HSFY1,IFI16,IRF4,KLF12,MAFG,MAX,MECP2,MEF2A,MEOX2,MTERF,MUTYH,NFYA,NLK,NR1I2,PAPSS2,PATZ1,PBX1,PDPK1,PQBP1,PRDM4,RAD23B,RPL32,RQCD1,SCAND1,SMAD2,STAT4,STAT5A,STUB1,SUPT4H1,TBX6,TGIF1,TLX2,TP53,TTF2,XBP1,ZBTB7B,ZFP36L1,ZNF169,ZNF444,ZNF449 |
| GO.0090304 | nucleic acid metabolic process | 65 | 4.51E-12 | BIRC5,CBFA2T3,CCND1,CDC25A,CDK2,CHEK2,CREB5,CWC27,DLX3,DPF2,DR1,EEF1D,ELK1,EZH2,FEN1,FOXA3,FOXR2,GMEB1,GTF2A2,GTF2H1,HEXIM1,HEYL,HMGB2,HNF1B,HORMAD1,HOXB6,HSFY1,IFI16,IRF4,KLF12,MAFG,MAX,MECP2,MEF2A,MEOX2,MTERF,MUTYH,NFYA,NLK,NR1I2,PATZ1,PBX1,PDPK1,PQBP1,PRDM4,RAD23B,RPL32,RQCD1,SCAND1,SMAD2,STAT4,STAT5A,STUB1,SUPT4H1,TBX6,TGIF1,TLX2,TP53,TTF2,XBP1,ZBTB7B,ZFP36L1,ZNF169,ZNF444,ZNF449 |
| GO.0044249 | cellular biosynthetic process | 64 | 2.56E-09 | AK2,BIRC5,CBFA2T3,CCND1,CDC25A,CDK2,CHEK2,CREB5,DLX3,DPF2,DR1,EEF1D,ELK1,EXT2,EZH2,FEN1,FOXA3,FOXR2,GMEB1,GTF2A2,GTF2H1,HEXIM1,HEYL,HNF1B,HOXB6,HSFY1,IFI16,IMPA1,IRF4,KLF12,MAFG,MAX,MECP2,MEF2A,MEOX2,MTERF,NFYA,NLK,NME5,NR1I2,PAPSS2,PATZ1,PBX1,PDPK1,PLD2,PQBP1,PRDM4,RQCD1,SCAND1,SMAD2,STAT4,STAT5A,SUPT4H1,TBX6,TGIF1,TLX2,TP53,TTF2,VEGFB,XBP1,ZBTB7B,ZNF169,ZNF444,ZNF449 |
| GO.1901576 | organic substance biosynthetic process | 64 | 5.01E-09 | AK2,BIRC5,CBFA2T3,CCND1,CDC25A,CDK2,CHEK2,CREB5,DLX3,DPF2,DR1,EEF1D,ELK1,EXT2,EZH2,FEN1,FOXA3,FOXR2,GMEB1,GTF2A2,GTF2H1,HEXIM1,HEYL,HNF1B,HOXB6,HSFY1,IFI16,IMPA1,IRF4,KLF12,MAFG,MAX,MECP2,MEF2A,MEOX2,MTERF,NFYA,NLK,NME5,NR1I2,PAPSS2,PATZ1,PBX1,PDPK1,PLD2,PQBP1,PRDM4,RQCD1,SCAND1,SMAD2,STAT4,STAT5A,SUPT4H1,TBX6,TGIF1,TLX2,TP53,TTF2,VEGFB,XBP1,ZBTB7B,ZNF169,ZNF444,ZNF449 |
| GO.0010467 | gene expression | 61 | 3.24E-10 | BAD,BIRC5,CASP7,CBFA2T3,CCND1,CHEK2,CREB5,CWC27,DLX3,DPF2,DR1,EEF1D,ELK1,EZH2,FOXA3,FOXR2,GMEB1,GTF2A2,GTF2H1,HEXIM1,HEYL,HNF1B,HOXB6,HSFY1,IFI16,IRF4,KLF12,MAFG,MAX,MECP2,MEF2A,MEOX2,MTERF,NFYA,NLK,NR1I2,PATZ1,PBX1,PDPK1,PQBP1,PRDM4,PSME2,RQCD1,SCAND1,SMAD2,STAT4,STAT5A,STUB1,SUPT4H1,TBX6,TGIF1,TLX2,TP53,TRAF2,TTF2,XBP1,ZBTB7B,ZFP36L1,ZNF169,ZNF444,ZNF449 |
| GO.0010468 | regulation of gene expression | 60 | 3.67E-10 | ACVR2A,BIRC5,BIRC7,CCNB1,CCND1,CDK2,CHEK2,CREB5,DLX1,DPF2,DR1,EEF1D,ELK1,EZR,FOXA3,FOXR2,GMEB1,GTF2A2,GTF2H1,HCFC2,HEXIM1,HMGB2,HOXB6,HSFY1,IFI16,IRF4,KIT,MAPK8,MEF2A,MEOX2,MSN,MTERF,NFE2L2,NLK,NR1I2,PDPK1,PHIP,PPP2CB,PQBP1,PRDM4,PRKCH,RQCD1,SCAND1,SMAD2,SMARCE1,STAP1,TBK1,TBX5,TBX6,TGIF1,TLX2,TP53,TRAF2,TTF2,UBE2V1,VEGFB,ZBTB7B,ZNF169,ZNF444,ZNF449 |
| GO.0048518 | positive regulation of biological process | 60 | 3.06E-06 | ACVR2A,BAD,BIRC5,BIRC7,CASP7,CCNB1,CCND1,CHEK2,CREB5,CTNNA2,DLX3,DSTYK,EEF1D,FAF1,FOXA3,GMEB1,GTF2A2,GTF2H1,HEXIM1,HMGB2,HNF1B,HRH2,IFI16,IRF4,KLF12,MAFG,MAPK8,MECP2,MEF2A,MEOX2,MSN,NFE2L2,NR1I2,PATZ1,PBX1,PDPK1,PKNOX1,PLD2,PRKCH,PSME2,RQCD1,SLC25A6,SMAD2,STAP1,STAT4,STAT5A,STK10,STMN1,STUB1,SUPT4H1,TBK1,TBX5,TBX6,TGIF1,TP53,TPM1,TRAF2,UBE2V1,VEGFB,ZBTB7B |
| GO.0034645 | cellular macromolecule biosynthetic process | 59 | 2.04E-10 | BIRC5,CBFA2T3,CCND1,CDC25A,CDK2,CHEK2,CREB5,DLX3,DPF2,DR1,EEF1D,ELK1,EXT2,EZH2,FEN1,FOXA3,FOXR2,GMEB1,GTF2A2,GTF2H1,HEXIM1,HEYL,HNF1B,HOXB6,HSFY1,IFI16,IRF4,KLF12,MAFG,MAX,MECP2,MEF2A,MEOX2,MTERF,NFYA,NLK,NR1I2,PATZ1,PBX1,PDPK1,PQBP1,PRDM4,RQCD1,SCAND1,SMAD2,STAT4,STAT5A,SUPT4H1,TBX6,TGIF1,TLX2,TP53,TTF2,VEGFB,XBP1,ZBTB7B,ZNF169,ZNF444,ZNF449 |
| GO.0034654 | nucleobase-containing compound biosynthetic process | 58 | 2.17E-12 | AK2,BIRC5,CBFA2T3,CCND1,CHEK2,CREB5,DLX3,DPF2,DR1,EEF1D,ELK1,EZH2,FOXA3,FOXR2,GMEB1,GTF2A2,GTF2H1,HEXIM1,HEYL,HNF1B,HOXB6,HSFY1,IFI16,IRF4,KLF12,MAFG,MAX,MECP2,MEF2A,MEOX2,MTERF,NFYA,NLK,NME5,NR1I2,PAPSS2,PATZ1,PBX1,PDPK1,PQBP1,PRDM4,RPL32,RQCD1,SCAND1,SMAD2,STAT4,STAT5A,SUPT4H1,TBX6,TGIF1,TLX2,TP53,TTF2,XBP1,ZBTB7B,ZNF169,ZNF444,ZNF449 |
| GO.0016070 | RNA metabolic process | 58 | 1.13E-10 | BIRC5,CBFA2T3,CCND1,CHEK2,CREB5,CWC27,DLX3,DPF2,DR1,EEF1D,ELK1,EZH2,FEN1,FOXA3,FOXR2,GMEB1,GTF2A2,GTF2H1,HEXIM1,HEYL,HNF1B,HOXB6,HSFY1,IFI16,IRF4,KLF12,MAFG,MAX,MECP2,MEF2A,MEOX2,MTERF,NFYA,NLK,NR1I2,PATZ1,PBX1,PDPK1,PQBP1,PRDM4,RPL32,RQCD1,SCAND1,SMAD2,STAT4,STAT5A,SUPT4H1,TBX6,TGIF1,TLX2,TP53,TTF2,XBP1,ZBTB7B,ZFP36L1,ZNF169,ZNF444,ZNF449 |
| GO.0044271 | cellular nitrogen compound biosynthetic process | 57 | 2.81E-10 | AK2,BIRC5,CBFA2T3,CCND1,CHEK2,CREB5,DLX3,DPF2,DR1,EEF1D,ELK1,EZH2,FOXA3,FOXR2,GMEB1,GTF2A2,GTF2H1,HEXIM1,HEYL,HNF1B,HOXB6,HSFY1,IFI16,IRF4,KLF12,MAFG,MAX,MECP2,MEF2A,MEOX2,MTERF,NFYA,NLK,NME5,NR1I2,PAPSS2,PATZ1,PBX1,PDPK1,PQBP1,PRDM4,RQCD1,SCAND1,SMAD2,STAT4,STAT5A,SUPT4H1,TBX6,TGIF1,TLX2,TP53,TTF2,XBP1,ZBTB7B,ZNF169,ZNF444,ZNF449 |
| GO.0019219 | regulation of nucleobase-containing compound metabolic process | 56 | 4.75E-09 | ACVR2A,BIRC5,CCNB1,CCND1,CDK2,CHEK2,CREB5,DLX1,DPF2,DR1,EEF1D,ELK1,FOXA3,FOXR2,GTF2A2,GTF2H1,HCFC2,HEXIM1,HMGB2,HOXB6,HRH2,HSFY1,IFI16,IRF4,KIT,MAFG,MAPK8,MEF2A,MEOX2,MTERF,NFE2L2,NLK,NR1I2,PDPK1,PHIP,PQBP1,PRDM4,PRKCH,RQCD1,SCAND1,SMAD2,SMARCE1,TBK1,TBX5,TBX6,TGIF1,TLX2,TP53,TRAF2,TTF2,UBE2V1,ZBTB7B,ZFP36L1,ZNF169,ZNF444,ZNF449 |
| GO.0051171 | regulation of nitrogen compound metabolic process | 56 | 7.17E-08 | ACVR2A,BIRC5,CCNB1,CCND1,CDK2,CHEK2,CREB5,DLX1,DPF2,DR1,EEF1D,ELK1,FOXA3,FOXR2,GTF2A2,GTF2H1,HCFC2,HEXIM1,HMGB2,HOXB6,HRH2,HSFY1,IFI16,IRF4,KIT,MAFG,MAPK8,MEF2A,MEOX2,MTERF,NFE2L2,NLK,NR1I2,PDPK1,PHIP,PQBP1,PRDM4,PRKCH,PSME2,RQCD1,SCAND1,SMAD2,SMARCE1,TBK1,TBX5,TBX6,TGIF1,TLX2,TP53,TRAF2,TTF2,UBE2V1,ZBTB7B,ZNF169,ZNF444,ZNF449 |
| GO.0048522 | positive regulation of cellular process | 56 | 1.15E-06 | ACVR2A,BAD,BIRC5,BIRC7,CASP7,CCNB1,CCND1,CHEK2,CREB5,CTNNA2,DLX3,DSTYK,EEF1D,FAF1,FOXA3,GMEB1,GTF2A2,GTF2H1,HEXIM1,HMGB2,HNF1B,HRH2,IFI16,IRF4,KLF12,MAPK8,MECP2,MEF2A,MEOX2,MSN,NFE2L2,NR1I2,PATZ1,PBX1,PDPK1,PKNOX1,PRKCH,PSME2,SMAD2,STAP1,STAT4,STAT5A,STK10,STMN1,STUB1,SUPT4H1,TBK1,TBX5,TBX6,TGIF1,TP53,TPM1,TRAF2,UBE2V1,VEGFB,WAS |
| GO.0032502 | developmental process | 56 | 1.23E-05 | ACVR2A,ASB1,BAG3,CBFA2T3,CCNB1,CCND1,CDK16,CDK2,CDKN2C,CHEK2,CKB,CTNNA2,DLX1,DLX3,ELK1,EXT2,EZH2,EZR,FOXA3,FOXR2,HEXIM1,HEYL,HMGB2,HORMAD1,HOXB6,HRH2,IFI16,JUNB,MAFG,MAPK8,MECP2,MEF2A,MSN,NDRG2,PATZ1,PDPK1,PKNOX1,PRKCH,PYGO2,RAC2,RQCD1,SCFD1,SERPINB5,SMAD2,SMARCE1,STAT5A,TBX5,TLX2,TP53,TPM1,UBE2V1,USH1C,VEGFB,WAS,ZBTB7B,ZFP36L1 |
| GO.0032774 | RNA biosynthetic process | 55 | 2.17E-12 | BIRC5,CBFA2T3,CCND1,CHEK2,CREB5,DLX3,DPF2,DR1,EEF1D,ELK1,EZH2,FOXA3,FOXR2,GMEB1,GTF2A2,GTF2H1,HEXIM1,HEYL,HNF1B,HOXB6,HSFY1,IFI16,IRF4,KLF12,MAFG,MAX,MECP2,MEF2A,MEOX2,MTERF,NFYA,NLK,NR1I2,PATZ1,PBX1,PDPK1,PQBP1,PRDM4,RPL32,RQCD1,SCAND1,SMAD2,STAT4,STAT5A,SUPT4H1,TBX6,TGIF1,TLX2,TP53,TTF2,XBP1,ZBTB7B,ZNF169,ZNF444,ZNF449 |
| GO.0006355 | regulation of transcription, DNA-templated | 55 | 3.28E-10 | ACVR2A,BIRC5,CCND1,CDK2,CHEK2,CREB5,DLX1,DPF2,DR1,EEF1D,ELK1,FOXA3,FOXR2,GMEB1,GTF2A2,GTF2H1,HCFC2,HEXIM1,HMGB2,HOXB6,HSFY1,IFI16,IRF4,KIT,MAFG,MAPK8,MEF2A,MEOX2,MTERF,NFE2L2,NLK,NR1I2,PDPK1,PHIP,PQBP1,PRDM4,PRKCH,RQCD1,SCAND1,SMAD2,SMARCE1,TBK1,TBX5,TBX6,TGIF1,TLX2,TP53,TRAF2,TTF2,UBE2V1,ZBTB7B,ZFP36L1,ZNF169,ZNF444,ZNF449 |
| GO.0051252 | regulation of RNA metabolic process | 55 | 9.04E-10 | ACVR2A,BIRC5,CCNB1,CCND1,CDK2,CHEK2,CREB5,DLX1,DPF2,DR1,EEF1D,ELK1,FOXA3,FOXR2,GTF2A2,GTF2H1,HCFC2,HEXIM1,HMGB2,HOXB6,HSFY1,IFI16,IRF4,KIT,MAFG,MAPK8,MEF2A,MEOX2,MTERF,NFE2L2,NLK,NR1I2,PDPK1,PHIP,PQBP1,PRDM4,PRKCH,RQCD1,SCAND1,SMAD2,SMARCE1,TBK1,TBX5,TBX6,TGIF1,TLX2,TP53,TRAF2,TTF2,UBE2V1,ZBTB7B,ZFP36L1,ZNF169,ZNF444,ZNF449 |
| GO.0031326 | regulation of cellular biosynthetic process | 55 | 8.56E-08 | ACVR2A,ASB1,BIRC5,CCND1,CDK2,CHEK2,CREB5,DLX1,DPF2,DR1,EEF1D,ELK1,FOXA3,FOXR2,GTF2A2,GTF2H1,HCFC2,HEXIM1,HMGB2,HOXB6,HRH2,HSFY1,IFI16,IRF4,KIT,MAFG,MAPK8,MEF2A,MEOX2,MTERF,NFE2L2,NLK,NR1I2,PDPK1,PHIP,PQBP1,PRDM4,PRKCH,RQCD1,SCAND1,SMAD2,SMARCE1,TBK1,TBX5,TBX6,TGIF1,TLX2,TP53,TRAF2,TTF2,UBE2V1,ZBTB7B,ZNF169,ZNF444,ZNF449 |
| GO.0044767 | single-organism developmental process | 55 | 2.33E-05 | ACVR2A,ASB1,BAG3,CBFA2T3,CCNB1,CCND1,CDK16,CDK2,CDKN2C,CHEK2,CKB,CTNNA2,DLX1,DLX3,ELK1,EXT2,EZH2,EZR,FOXA3,FOXR2,HEXIM1,HEYL,HMGB2,HORMAD1,HOXB6,HRH2,IFI16,JUNB,MAFG,MAPK8,MECP2,MEF2A,MSN,NDRG2,PATZ1,PDPK1,PKNOX1,PRKCH,PYGO2,RAC2,SCFD1,SERPINB5,SMAD2,SMARCE1,STAT5A,TBX5,TBX6,TLX2,TP53,TPM1,UBE2V1,USH1C,VEGFB,ZBTB7B,ZFP36L1 |
| GO.0006351 | transcription, DNA-templated | 54 | 2.17E-12 | BIRC5,CBFA2T3,CCND1,CHEK2,CREB5,DLX3,DPF2,DR1,EEF1D,ELK1,EZH2,FOXA3,FOXR2,GMEB1,GTF2A2,GTF2H1,HEXIM1,HEYL,HNF1B,HOXB6,HSFY1,IFI16,IRF4,KLF12,MAFG,MAX,MECP2,MEF2A,MEOX2,MTERF,NFYA,NLK,NR1I2,PATZ1,PBX1,PDPK1,PQBP1,PRDM4,RQCD1,SCAND1,SMAD2,STAT4,STAT5A,SUPT4H1,TBX6,TGIF1,TLX2,TP53,TTF2,XBP1,ZBTB7B,ZNF169,ZNF444,ZNF449 |
| GO.1903506 | regulation of nucleic acid-templated transcription | 54 | 9.53E-10 | ACVR2A,BIRC5,CCND1,CDK2,CHEK2,CREB5,DLX1,DPF2,DR1,EEF1D,ELK1,FOXA3,FOXR2,GTF2A2,GTF2H1,HCFC2,HEXIM1,HMGB2,HOXB6,HSFY1,IFI16,IRF4,KIT,MAFG,MAPK8,MEF2A,MEOX2,MTERF,NFE2L2,NLK,NR1I2,PDPK1,PHIP,PQBP1,PRDM4,PRKCH,RQCD1,SCAND1,SMAD2,SMARCE1,TBK1,TBX5,TBX6,TGIF1,TLX2,TP53,TRAF2,TTF2,UBE2V1,ZBTB7B,ZFP36L1,ZNF169,ZNF444,ZNF449 |
| GO.2000112 | regulation of cellular macromolecule biosynthetic process | 54 | 2.41E-08 | ACVR2A,BIRC5,CCND1,CDK2,CHEK2,CREB5,DLX1,DPF2,DR1,EEF1D,ELK1,FOXA3,FOXR2,GMEB1,GTF2A2,GTF2H1,HCFC2,HEXIM1,HMGB2,HOXB6,HSFY1,IFI16,IRF4,KIT,MAFG,MAPK8,MEF2A,MEOX2,MTERF,NFE2L2,NLK,NR1I2,PDPK1,PHIP,PQBP1,PRDM4,PRKCH,RQCD1,SCAND1,SMAD2,SMARCE1,TBK1,TBX5,TBX6,TGIF1,TLX2,TP53,TRAF2,TTF2,UBE2V1,ZBTB7B,ZNF169,ZNF444,ZNF449 |
| GO.0010556 | regulation of macromolecule biosynthetic process | 54 | 4.74E-08 | ACVR2A,ASB1,BIRC5,CCND1,CDK2,CHEK2,CREB5,DLX1,DPF2,DR1,EEF1D,ELK1,FOXA3,FOXR2,GTF2A2,GTF2H1,HCFC2,HEXIM1,HMGB2,HOXB6,HSFY1,IFI16,IRF4,KIT,MAFG,MAPK8,MEF2A,MEOX2,MTERF,NFE2L2,NLK,NR1I2,PDPK1,PHIP,PQBP1,PRDM4,PRKCH,RQCD1,SCAND1,SMAD2,SMARCE1,TBK1,TBX5,TBX6,TGIF1,TLX2,TP53,TRAF2,TTF2,UBE2V1,ZBTB7B,ZNF169,ZNF444,ZNF449 |
| GO.0048856 | anatomical structure development | 54 | 1.45E-06 | ACVR2A,ASB1,BAG3,CBFA2T3,CCNB1,CCND1,CDK16,CDK2,CDKN2C,CKB,CTNNA2,DLX1,DLX3,EXT2,EZH2,EZR,FOXA3,FOXR2,HEXIM1,HEYL,HMGB2,HORMAD1,HOXB6,HRH2,IFI16,JUNB,MAFG,MAPK8,MECP2,MEF2A,MEOX2,MSN,NDRG2,PATZ1,PDPK1,PKNOX1,PRKCH,PYGO2,RAC2,SCFD1,SERPINB5,SMAD2,SMARCE1,STAT5A,TBX5,TGIF1,TLX2,TP53,TPM1,USH1C,VEGFB,WAS,ZBTB7B,ZFP36L1 |
| GO.0048519 | negative regulation of biological process | 54 | 3.59E-06 | ASB1,BAD,BIRC5,BIRC7,BTG3,CBLC,CCNB1,CDK2,CDKN2C,CHEK2,CTNNA2,DLX1,DR1,FAF1,GTF2H1,HCFC2,HEXIM1,HMGB2,HNF1B,HORMAD1,IFI16,IRF4,JUNB,KLK3,MAPK8,MEF2A,NDRG2,NFE2L2,NLK,NME5,NR1I2,PATZ1,PDPK1,PHIP,PLD2,PRKCH,PSME2,RQCD1,SCFD1,SERPINB5,SMAD2,SMARCE1,STAP1,STAT5A,STMN1,STUB1,TBX5,TBX6,TLX2,TP53,TPM1,VEGFB,WAS,XBP1 |
| GO.0009893 | positive regulation of metabolic process | 53 | 1.28E-08 | ACVR2A,BAD,BIRC7,CASP7,CCND1,CDK2,CHEK2,CREB5,DLX3,DSTYK,ELK1,EZH2,EZR,FOXA3,GMEB1,GTF2A2,GTF2H1,HMGB2,HNF1B,HRH2,IFI16,IRF4,KIT,KLF12,MAFG,MAPK8,MECP2,MEF2A,MEOX2,MSN,NFE2L2,NR1I2,PATZ1,PBX1,PDPK1,PKNOX1,PRKCH,PSME2,SMAD2,STAP1,STAT4,STAT5A,STK10,SUPT4H1,TBK1,TBX5,TBX6,TP53,TPM1,TRAF2,UBE2V1,VEGFB,ZBTB7B |
| GO.0051716 | cellular response to stimulus | 53 | 0.0374 | ACVR2A,ASB1,BIRC5,BIRC7,CASP7,CBLC,CCNB1,CDC25A,CDK2,CHEK2,DPF2,DSTYK,EEF1D,EXT2,EZH2,EZR,FEN1,FOXA3,GTF2H1,HMGB2,HRH2,IFI16,IMPA1,INPP1,IRF4,KLK3,LRRFIP2,MEF2A,MUTYH,NDRG2,NFE2L2,NLK,NR1I2,PAPSS2,PDGFRL,PDPK1,PHIP,PSME2,PYGO2,RAD23B,RQCD1,SMAD2,STAP1,STK10,STK38L,STMN1,STUB1,TBK1,TBX6,TP53,TPM1,VEGFB,WAS |
| GO.0044700 | single organism signaling | 52 | 0.00137 | ACVR2A,ASB1,BIRC5,BIRC7,CASP7,CBLC,CDK16,CDK2,CHEK2,DPF2,EEF1D,ELK1,EXT2,EZR,HEYL,HNF1B,HRH2,IFI16,IMPA1,INPP1,IRF4,JUNB,KLK3,LRRFIP2,MAPK8,MECP2,MEF2A,NDRG2,NFE2L2,NLK,NR1I2,PDGFRL,PDPK1,PHIP,PPP2CB,PRDM4,PSME2,PYGO2,RQCD1,SMAD2,STAP1,STK10,STK38L,STMN1,TBK1,TBX5,TBX6,TP53,TRAF2,UBE2V1,VEGFB,WAS |
| GO.0007154 | cell communication | 52 | 0.00238 | ACVR2A,ASB1,BIRC5,BIRC7,CASP7,CBLC,CDK16,CDK2,CHEK2,DPF2,EEF1D,ELK1,EXT2,EZR,FOXA3,HEYL,HNF1B,HRH2,IFI16,IMPA1,INPP1,IRF4,JUNB,KLK3,LRRFIP2,MAPK8,MECP2,MEF2A,NDRG2,NLK,NR1I2,PDGFRL,PDPK1,PHIP,PPP2CB,PSME2,PYGO2,RQCD1,SMAD2,STAP1,STK10,STK38L,STMN1,TBK1,TBX5,TBX6,TP53,TRAF2,TXN2,UBE2V1,VEGFB,WAS |
| GO.0010604 | positive regulation of macromolecule metabolic process | 51 | 2.05E-11 | ACVR2A,BAD,BIRC7,CASP7,CCND1,CDK2,CHEK2,CREB5,DLX3,DSTYK,ELK1,EZH2,EZR,FOXA3,GMEB1,GTF2A2,GTF2H1,HMGB2,HNF1B,IFI16,IRF4,KIT,KLF12,MAFG,MAPK8,MECP2,MEF2A,MEOX2,MSN,NFE2L2,NR1I2,PATZ1,PBX1,PDPK1,PKNOX1,PRKCH,PSME2,SMAD2,STAP1,STAT4,STAT5A,STK10,SUPT4H1,TBK1,TBX5,TBX6,TP53,TRAF2,UBE2V1,VEGFB,ZBTB7B |
| GO.0048523 | negative regulation of cellular process | 51 | 5.97E-06 | ASB1,BAD,BIRC5,BIRC7,BTG3,CBLC,CCNB1,CDK2,CDKN2C,CHEK2,DLX1,DR1,FAF1,HCFC2,HEXIM1,HMGB2,HNF1B,HORMAD1,IFI16,IRF4,JUNB,MAPK8,MEF2A,NDRG2,NFE2L2,NLK,NME5,NR1I2,PATZ1,PDPK1,PHIP,PLD2,PRKCH,PSME2,RQCD1,SCFD1,SERPINB5,SMAD2,SMARCE1,STAP1,STAT5A,STMN1,STUB1,TBX5,TBX6,TLX2,TP53,TPM1,VEGFB,WAS,XBP1 |
| GO.0007275 | multicellular organismal development | 51 | 2.10E-05 | ACVR2A,ASB1,BAG3,CBFA2T3,CCNB1,CCND1,CDK16,CDK2,CDKN2C,CKB,CTNNA2,DLX1,DLX3,EXT2,EZH2,EZR,FOXA3,HEXIM1,HEYL,HMGB2,HORMAD1,HOXB6,HRH2,IFI16,JUNB,MAFG,MAPK8,MECP2,MEF2A,NDRG2,NME5,PATZ1,PBX1,PDPK1,PKNOX1,PRKCH,PYGO2,RAC2,SERPINB5,SMAD2,SMARCE1,STAT5A,TBX5,TBX6,TLX2,TP53,TPM1,USH1C,VEGFB,ZBTB7B,ZFP36L1 |
| GO.0031325 | positive regulation of cellular metabolic process | 49 | 1.20E-09 | ACVR2A,BAD,BIRC7,CASP7,CCND1,CDK2,CHEK2,CREB5,DLX3,DSTYK,ELK1,EZH2,EZR,FOXA3,GMEB1,GTF2A2,GTF2H1,HMGB2,HNF1B,HRH2,IFI16,IRF4,KIT,KLF12,MECP2,MEF2A,MEOX2,MSN,NFE2L2,NR1I2,PATZ1,PBX1,PDPK1,PKNOX1,PRKCH,PSME2,SMAD2,STAP1,STAT4,STAT5A,STK10,SUPT4H1,TBK1,TBX5,TBX6,TP53,TRAF2,UBE2V1,VEGFB |
| GO.0048731 | system development | 48 | 6.08E-06 | ACVR2A,ASB1,BAG3,CBFA2T3,CCNB1,CCND1,CDK16,CDK2,CDKN2C,CKB,CTNNA2,DLX1,DLX3,EZH2,EZR,FOXA3,HEXIM1,HEYL,HMGB2,HOXB6,HRH2,IFI16,JUNB,MAPK8,MECP2,MEF2A,MEOX2,NDRG2,NME5,PATZ1,PBX1,PDPK1,PKNOX1,PRKCH,PYGO2,RAC2,SERPINB5,SMARCE1,STAT5A,TBX5,TBX6,TGIF1,TLX2,TP53,TPM1,USH1C,VEGFB,ZFP36L1 |
| GO.0007165 | signal transduction | 48 | 0.0045 | ACVR2A,ASB1,BIRC5,BIRC7,CASP7,CBLC,CDK2,CHEK2,DPF2,EEF1D,ELK1,EXT2,EZR,HEYL,HRH2,IFI16,IMPA1,INPP1,IRF4,JUNB,KLK3,LRRFIP2,MAPK8,MEF2A,NDRG2,NFE2L2,NLK,NR1I2,PDGFRL,PDPK1,PHIP,PPP2CB,PRDM4,PSME2,PYGO2,RQCD1,SMAD2,STAP1,STK10,STK38L,STMN1,TBK1,TBX6,TP53,TRAF2,UBE2V1,VEGFB,WAS |
| GO.0010628 | positive regulation of gene expression | 44 | 6.36E-13 | ACVR2A,CCNB1,CDK2,CHEK2,CREB5,DLX3,ELK1,EZR,FOXA3,GMEB1,GTF2A2,GTF2H1,HMGB2,HNF1B,IFI16,IRF4,KIT,KLF12,MAFG,MAPK8,MECP2,MEF2A,MEOX2,MSN,NFE2L2,NR1I2,PATZ1,PBX1,PHIP,PKNOX1,PRKCH,SMAD2,STAP1,STAT4,STAT5A,SUPT4H1,TBK1,TBX5,TBX6,TP53,TRAF2,UBE2V1,XBP1,ZBTB7B |
| GO.0030154 | cell differentiation | 44 | 7.06E-06 | CBFA2T3,CCNB1,CCND1,CDK16,CDKN2C,CTNNA2,DLX1,DLX3,ELK1,EXT2,EZH2,EZR,FOXA3,FOXR2,HEYL,HMGB2,HNF1B,HORMAD1,HRH2,IFI16,JUNB,MAPK8,MECP2,MEF2A,MSN,NDRG2,NME5,PATZ1,PDPK1,PKNOX1,PRKCH,PYGO2,RAC2,SMAD2,STAT5A,TBX5,TBX6,TGIF1,TLX2,TPM1,UBE2V1,USH1C,ZBTB7B,ZFP36L1 |
| GO.0048869 | cellular developmental process | 44 | 2.49E-05 | CBFA2T3,CCNB1,CCND1,CDK16,CDKN2C,CTNNA2,DLX1,DLX3,ELK1,EXT2,EZH2,EZR,FOXA3,FOXR2,HEYL,HMGB2,HNF1B,HORMAD1,HRH2,IFI16,JUNB,MAPK8,MECP2,MEF2A,MSN,NDRG2,PATZ1,PDPK1,PKNOX1,PRKCH,PYGO2,RAC2,SCFD1,SMAD2,STAT5A,TBX5,TBX6,TGIF1,TLX2,TPM1,UBE2V1,USH1C,ZBTB7B,ZFP36L1 |
| GO.0019538 | protein metabolic process | 44 | 0.00257 | ASB1,BAD,BAG3,BIRC5,BIRC7,CASP7,CBLC,CCND1,CDC25A,CDK16,CDK18,CDK2,CHEK2,CWC27,DR1,DSTYK,EEF1D,ELK1,EXT2,EZH2,FAF1,GTF2H1,IFI16,IRF4,MAPK8,MECP2,MEF2A,NFE2L2,PDGFRL,PDPK1,PSME2,PTPN20A,RAD23B,SLC25A6,SMAD2,STAT4,STAT5A,STK10,STK38L,STUB1,TBK1,TXN2,VEGFB,XBP1 |
| GO.0044267 | cellular protein metabolic process | 43 | 0.000167 | ASB1,BAG3,BIRC5,BIRC7,CBLC,CCND1,CDC25A,CDK16,CDK18,CDK2,CHEK2,CWC27,DR1,DSTYK,EEF1D,ELK1,EXT2,EZH2,FAF1,GTF2H1,IRF4,KLK3,MAPK8,MECP2,MEF2A,NFE2L2,PDGFRL,PDPK1,PSME2,PTPN20A,RAD23B,SLC25A6,SMAD2,STAT4,STAT5A,STK10,STK38L,STUB1,TBK1,TRAF2,TXN2,VEGFB,XBP1 |
| GO.0048513 | organ development | 41 | 3.27E-06 | ACVR2A,ASB1,BAG3,CBFA2T3,CCNB1,CCND1,CDK2,CKB,CTNNA2,DLX1,DLX3,EZH2,FOXA3,HEXIM1,HEYL,HMGB2,HOXB6,HRH2,IFI16,JUNB,MECP2,MEF2A,MEOX2,NDRG2,NME5,PAPSS2,PATZ1,PBX1,PDPK1,PKNOX1,PYGO2,SERPINB5,STAT5A,STMN1,TBX5,TGIF1,TP53,TPM1,USH1C,VEGFB,ZFP36L1 |
| GO.0065009 | regulation of molecular function | 40 | 1.06E-05 | ACVR2A,BAD,BIRC5,BIRC7,CASP7,CBLC,CCNB1,CCND1,CDC25A,CDK2,CDKN2C,DSTYK,EZH2,FAF1,GTF2A2,GTF2H1,HEXIM1,HEYL,HMGB2,HRH2,IFI16,IRF4,KIT,MAPK8,PBX1,PDPK1,PRKCH,PSME2,PYGO2,RAC2,RQCD1,SERPINB5,SMAD2,STAP1,STK10,STUB1,TPM1,TRAF2,UBE2V1,WAS |
| GO.0048583 | regulation of response to stimulus | 40 | 0.00127 | ACVR2A,BAG3,BIRC5,BIRC7,CBLC,CCND1,CDK16,CTNNA2,DLX1,DSTYK,EEF1D,ELK1,EZH2,HEXIM1,HEYL,HMGB2,HNF1B,IFI16,MAPK8,MEF2A,NDRG2,NFE2L2,NLK,NME5,PHIP,PRKCH,PSME2,RQCD1,SCFD1,SLC25A6,SMAD2,STAP1,STAT5A,STUB1,TBK1,TGIF1,UBE2V1,VEGFB,WAS,XBP1 |
| GO.0006357 | regulation of transcription from RNA polymerase II promoter | 39 | 1.36E-10 | ACVR2A,CBFA2T3,CCND1,DLX1,DLX3,DR1,ELK1,FOXA3,FOXR2,GMEB1,GTF2A2,GTF2H1,HCFC2,HEXIM1,HMGB2,IFI16,IRF4,MAX,MECP2,MEF2A,MEOX2,NFE2L2,NFYA,NR1I2,PATZ1,PBX1,PHIP,PKNOX1,SCAND1,SMAD2,SMARCE1,STAT4,TBK1,TBX5,TBX6,TGIF1,TP53,ZBTB7B,ZNF444 |
| GO.0045935 | positive regulation of nucleobase-containing compound metabolic process | 39 | 1.36E-10 | ACVR2A,CCNB1,CDK2,CHEK2,CREB5,DLX3,ELK1,FOXA3,GMEB1,GTF2A2,GTF2H1,HMGB2,HNF1B,HRH2,IFI16,IRF4,KIT,KLF12,MECP2,MEF2A,MEOX2,NFE2L2,NR1I2,PATZ1,PBX1,PHIP,PKNOX1,PRKCH,SMAD2,STAT4,STAT5A,SUPT4H1,TBK1,TBX5,TBX6,TP53,TRAF2,UBE2V1,XBP1 |
| GO.0051246 | regulation of protein metabolic process | 39 | 2.66E-06 | ACVR2A,ASB1,BAD,BIRC5,BIRC7,CASP7,CBLC,CCND1,CDC25A,CDK2,CDKN2C,CHEK2,DSTYK,EZH2,EZR,FAF1,GTF2H1,HEXIM1,IFI16,IRF4,KIT,MAPK8,MECP2,MSN,NDRG2,PDPK1,PSME2,PYGO2,RAC2,RAD23B,RQCD1,SERPINB5,STAP1,STAT5A,STK10,TBK1,TP53,VEGFB,ZFP36L1 |
| GO.0051254 | positive regulation of RNA metabolic process | 38 | 2.83E-11 | ACVR2A,CCNB1,CDK2,CHEK2,CREB5,DLX3,ELK1,FOXA3,GMEB1,GTF2A2,GTF2H1,HMGB2,HNF1B,IFI16,IRF4,KIT,KLF12,MECP2,MEF2A,MEOX2,NFE2L2,NR1I2,PATZ1,PBX1,PHIP,PKNOX1,PRKCH,SMAD2,STAT4,STAT5A,SUPT4H1,TBK1,TBX5,TBX6,TP53,TRAF2,UBE2V1,XBP1 |
| GO.0031328 | positive regulation of cellular biosynthetic process | 38 | 9.66E-10 | ACVR2A,CDK2,CHEK2,CREB5,DLX3,ELK1,FOXA3,GMEB1,GTF2A2,GTF2H1,HMGB2,HNF1B,HRH2,IFI16,IRF4,KIT,KLF12,MECP2,MEF2A,MEOX2,NFE2L2,NR1I2,PATZ1,PBX1,PHIP,PKNOX1,PRKCH,SMAD2,STAT4,STAT5A,SUPT4H1,TBK1,TBX5,TBX6,TP53,TRAF2,UBE2V1,XBP1 |
| GO.0051239 | regulation of multicellular organismal process | 38 | 3.59E-06 | ACVR2A,ASB1,CCNB1,CCND1,DLX1,EZR,HEYL,HMGB2,HNF1B,HRH2,IFI16,IRF4,KIT,KLK3,MAFG,MAPK8,MECP2,MEF2A,NDRG2,NFE2L2,NFYA,PBX1,PDPK1,PQBP1,PRKCH,PYGO2,SMAD2,STAT5A,TBK1,TBX5,TBX6,TGIF1,TLX2,TP53,TPM1,TRAF2,VEGFB,XBP1 |
| GO.0010646 | regulation of cell communication | 38 | 0.000285 | ACVR2A,BIRC5,BIRC7,CBLC,CCND1,CDK16,DLX1,DSTYK,EEF1D,EZH2,HEXIM1,HEYL,HMGB2,HNF1B,HRH2,IRF4,MAPK8,MECP2,NDRG2,NFE2L2,NLK,NME5,PHIP,PLD2,PRKCH,PSME2,RAC2,RQCD1,SCFD1,SLC25A6,SMAD2,STAP1,STUB1,TBK1,TGIF1,UBE2V1,VEGFB,XBP1 |
| GO.0045893 | positive regulation of transcription, DNA-templated | 37 | 3.52E-11 | ACVR2A,CDK2,CHEK2,CREB5,DLX3,ELK1,FOXA3,GMEB1,GTF2A2,GTF2H1,HMGB2,HNF1B,IFI16,IRF4,KIT,KLF12,MECP2,MEF2A,MEOX2,NFE2L2,NR1I2,PATZ1,PBX1,PHIP,PKNOX1,PRKCH,SMAD2,STAT4,STAT5A,SUPT4H1,TBK1,TBX5,TBX6,TP53,TRAF2,UBE2V1,XBP1 |
| GO.0023051 | regulation of signaling | 37 | 0.00023 | ACVR2A,BIRC5,BIRC7,CBLC,CCND1,CDK16,DLX1,DSTYK,EEF1D,EZH2,HEXIM1,HEYL,HMGB2,HNF1B,HRH2,IRF4,MAPK8,MECP2,NDRG2,NFE2L2,NLK,NME5,PHIP,PLD2,PRKCH,PSME2,RAC2,RQCD1,SLC25A6,SMAD2,STAP1,STUB1,TBK1,TGIF1,UBE2V1,VEGFB,XBP1 |
| GO.0009892 | negative regulation of metabolic process | 36 | 0.000108 | ASB1,BIRC5,BIRC7,CBLC,CCNB1,CCND1,CDKN2C,CHEK2,DLX1,DR1,GTF2H1,HCFC2,HEXIM1,HMGB2,HNF1B,IFI16,JUNB,MEF2A,NDRG2,NR1I2,PATZ1,PBX1,PDPK1,PSME2,RQCD1,SCFD1,SERPINB5,SMAD2,SMARCE1,STAP1,TBK1,TBX6,TGIF1,TRAF2,VEGFB,XBP1 |
| GO.0006464 | cellular protein modification process | 36 | 0.000372 | ASB1,BIRC5,BIRC7,CBLC,CCNB1,CCND1,CDC25A,CDK16,CDK18,CDK2,CHEK2,CWC27,DR1,DSTYK,ELK1,EXT2,EZH2,GTF2H1,IRF4,MAPK8,MECP2,MEF2A,NFE2L2,PDGFRL,PDPK1,PSME2,PTPN20A,SMAD2,STAT4,STAT5A,STK10,STK38L,STUB1,TBK1,TRAF2,VEGFB |
| GO.0043412 | macromolecule modification | 36 | 0.00107 | ASB1,BIRC5,BIRC7,CBLC,CCNB1,CCND1,CDC25A,CDK16,CDK18,CDK2,CHEK2,CWC27,DR1,DSTYK,ELK1,EXT2,GTF2H1,IRF4,MAPK8,MECP2,MEF2A,MUTYH,NFE2L2,PDGFRL,PDPK1,PSME2,PTPN20A,SMAD2,STAT4,STAT5A,STK10,STK38L,STUB1,TBK1,TRAF2,VEGFB |
| GO.0032268 | regulation of cellular protein metabolic process | 35 | 3.83E-05 | ACVR2A,BAD,BIRC5,BIRC7,CASP7,CBLC,CCND1,CDC25A,CDK2,CDKN2C,DSTYK,EZH2,EZR,FAF1,GTF2H1,HEXIM1,IFI16,KIT,MAPK8,MECP2,MSN,NDRG2,PDPK1,PSME2,PYGO2,RAC2,RAD23B,RQCD1,SERPINB5,STAP1,STK10,TBK1,TP53,VEGFB,ZFP36L1 |
| GO.0006950 | response to stress | 35 | 0.0483 | BAG3,BIRC7,CCNB1,CDK2,CHEK2,ELK1,EZH2,FEN1,FOXA3,GTF2H1,HBG1,HMGB2,IFI16,IRF4,KIF9,KIT,MAFG,MECP2,MEF2A,MUTYH,NFE2L2,PAPSS2,PLD2,PPP2CB,PRKCH,PSME2,RAC2,RAD23B,SCFD1,STK10,STUB1,TP53,TPM1,VEGFB,WAS |
| GO.0009653 | anatomical structure morphogenesis | 34 | 1.93E-05 | ACVR2A,CTNNA2,DLX1,DLX3,EXT2,EZR,FOXA3,FOXR2,HEYL,HOXB6,HRH2,JUNB,MAPK8,MEF2A,MEOX2,NME5,PBX1,PKNOX1,PQBP1,RAC2,SCFD1,SERPINB5,SMAD2,STAT5A,STMN1,TBX5,TGIF1,TLX2,TP53,TPM1,USH1C,VEGFB,XBP1,ZFP36L1 |
| GO.0010605 | negative regulation of macromolecule metabolic process | 34 | 7.12E-05 | ASB1,BIRC5,BIRC7,CBLC,CCNB1,CCND1,CDKN2C,CHEK2,DLX1,DR1,GTF2H1,HCFC2,HEXIM1,HMGB2,HNF1B,IFI16,JUNB,MEF2A,NDRG2,NR1I2,PATZ1,PBX1,PDPK1,PSME2,RQCD1,SERPINB5,SMAD2,SMARCE1,TBK1,TBX6,TGIF1,TRAF2,VEGFB,XBP1 |
| GO.0009966 | regulation of signal transduction | 34 | 0.000314 | ACVR2A,BAD,BIRC5,BIRC7,CBLC,CCND1,DLX1,DSTYK,EEF1D,EZH2,HEXIM1,HEYL,HMGB2,HNF1B,IRF4,MAPK8,NDRG2,NFE2L2,NLK,NME5,PHIP,PLD2,PRKCH,PSME2,RAC2,RQCD1,SMAD2,STAP1,STUB1,TBK1,TGIF1,UBE2V1,VEGFB,XBP1 |
| GO.0006996 | organelle organization | 34 | 0.00689 | BIRC5,CCNB1,CCND1,CDC25A,CDK2,CEP55,CHEK2,DR1,EZH2,FEN1,FOXA3,HMGB2,HORMAD1,IRF4,KIF9,KIT,MECP2,MEF2A,MTERF,NME5,PDPK1,PHIP,PLD2,PPP2CB,PYGO2,RAC2,RAD23B,SLC25A6,SMARCE1,STMN1,SUPT4H1,TPM1,USH1C,XBP1 |
| GO.0006796 | phosphate-containing compound metabolic process | 32 | 6.99E-06 | AK2,BAD,BIRC5,BIRC7,CCNB1,CCND1,CDC25A,CDK16,CDK18,CDK2,CHEK2,CKB,DSTYK,ELK1,GTF2H1,INPP1,MAPK8,MECP2,MEF2A,MUTYH,PAPSS2,PDGFRL,PDPK1,PLD2,PTPN20A,SMAD2,STAT4,STAT5A,STK10,STK38L,TBK1,XYLB |
| GO.0050790 | regulation of catalytic activity | 32 | 0.000398 | ACVR2A,BAD,BIRC5,BIRC7,CASP7,CBLC,CCNB1,CCND1,CDC25A,CDK2,CDKN2C,DSTYK,EZH2,FAF1,GTF2H1,HEXIM1,HMGB2,HRH2,IFI16,KIT,MAPK8,PDPK1,PSME2,PYGO2,RAC2,SERPINB5,STAP1,STK10,STUB1,TPM1,TRAF2,WAS |
| GO.0031324 | negative regulation of cellular metabolic process | 32 | 0.00051 | ASB1,BIRC5,BIRC7,CBLC,CCNB1,CCND1,CDKN2C,CHEK2,DLX1,DR1,HCFC2,HEXIM1,HMGB2,HNF1B,IFI16,JUNB,MEF2A,NDRG2,NR1I2,PATZ1,PBX1,PDPK1,PSME2,SCFD1,SERPINB5,SMAD2,SMARCE1,STAP1,TBX6,TGIF1,TRAF2,XBP1 |
| GO.0050793 | regulation of developmental process | 31 | 0.000376 | ACVR2A,CCNB1,CCND1,CTNNA2,DLX1,EZR,HEYL,HMGB2,HNF1B,IRF4,JUNB,KLK3,MAFG,MAPK8,MECP2,MEF2A,NFE2L2,NFYA,PHIP,PQBP1,PRKCH,PYGO2,SMAD2,STAT5A,TBX5,TBX6,TGIF1,TLX2,TP53,VEGFB,XBP1 |
| GO.0051128 | regulation of cellular component organization | 31 | 0.000608 | BAD,BIRC5,CCNB1,CDKN2C,CTNNA2,FAF1,HNF1B,HORMAD1,KIF9,MAPK8,MECP2,MSN,PDPK1,PHIP,PLD2,PQBP1,PRDM4,PRKCH,PYGO2,RAC2,SCFD1,SMAD2,STAP1,STK38L,STUB1,TBX5,TBX6,TLX2,TPM1,WAS,XBP1 |
| GO.0010033 | response to organic substance | 31 | 0.00579 | ACVR2A,CCNB1,CCND1,CDK2,DSTYK,EZH2,HEYL,HMGB2,HNF1B,IRF4,JUNB,MAPK8,MEF2A,NFE2L2,NLK,NR1I2,PDGFRL,PDPK1,PHIP,PPP2CB,PRDM4,RQCD1,SMAD2,STAP1,STAT4,STUB1,TGIF1,TP53,TRAF2,TXN2,XBP1 |
| GO.0045944 | positive regulation of transcription from RNA polymerase II promoter | 30 | 3.28E-10 | ACVR2A,DLX3,ELK1,FOXA3,GMEB1,GTF2A2,GTF2H1,HMGB2,HNF1B,IFI16,IRF4,KLF12,MEF2A,MEOX2,NFE2L2,NFYA,NR1I2,PATZ1,PBX1,PHIP,PKNOX1,SMAD2,STAT4,STAT5A,SUPT4H1,TBK1,TBX5,TBX6,TP53,XBP1 |
| GO.0035556 | intracellular signal transduction | 30 | 9.68E-05 | ACVR2A,ASB1,BIRC5,BIRC7,CASP7,CDK2,CHEK2,ELK1,EZR,IFI16,KIT,KLK3,MAPK8,MEF2A,NLK,PDPK1,PRKCH,PSME2,RAC2,SMAD2,STAT4,STAT5A,STK10,STK38L,STMN1,TBK1,TP53,TRAF2,UBE2V1,WAS |
| GO.0007166 | cell surface receptor signaling pathway | 30 | 0.000721 | ACVR2A,CBLC,ELK1,HEYL,IRF4,JUNB,LRRFIP2,MAPK8,MEF2A,NDRG2,NLK,PDGFRL,PDPK1,PHIP,PLD2,PPP2CB,PRDM4,PSME2,PYGO2,RQCD1,SMAD2,STAP1,STAT4,STAT5A,STUB1,TRAF2,UBE2V1,VEGFB,WAS,XBP1 |
| GO.0016310 | phosphorylation | 29 | 6.02E-09 | AK2,BIRC5,BIRC7,CCNB1,CCND1,CDK16,CDK18,CDK2,CHEK2,CKB,DSTYK,ELK1,GTF2H1,IMPA1,INPP1,MAPK8,MECP2,MEF2A,NME5,PAPSS2,PDGFRL,PDPK1,SMAD2,STAT4,STAT5A,STK10,STK38L,TBK1,XYLB |
| GO.0007399 | nervous system development | 29 | 0.00101 | BAG3,CDK16,CDKN2C,CKB,CTNNA2,DLX1,EZH2,EZR,HEYL,HMGB2,HNF1B,KIT,MAPK8,MECP2,MEF2A,NDRG2,NME5,PBX1,PRKCH,PYGO2,RAC2,SMARCE1,TBX6,TGIF1,TLX2,TP53,USH1C,XBP1,ZFP36L1 |
| GO.0071310 | cellular response to organic substance | 28 | 0.0008 | ACVR2A,CCNB1,CCND1,CDK2,DSTYK,HEYL,HMGB2,IRF4,JUNB,MAPK8,MEF2A,NFE2L2,NLK,NR1I2,PDGFRL,PDPK1,PHIP,PPP2CB,PRDM4,RQCD1,SMAD2,STAP1,STAT4,STUB1,TGIF1,TP53,TRAF2,XBP1 |
| GO.0048584 | positive regulation of response to stimulus | 28 | 0.00127 | ACVR2A,BIRC7,DSTYK,EEF1D,ELK1,EZH2,FAF1,HEXIM1,IFI16,MAPK8,MEF2A,NFE2L2,PDPK1,PHIP,PLD2,PRKCH,PSME2,RAC2,SLC25A6,SMAD2,STAP1,STAT5A,TBK1,TP53,UBE2V1,VEGFB,WAS,XBP1 |
| GO.2000026 | regulation of multicellular organismal development | 27 | 0.000104 | ACVR2A,CCNB1,CCND1,DLX1,HEYL,HMGB2,HNF1B,IRF4,KIT,KLK3,MAFG,MAPK8,MECP2,NFE2L2,PBX1,PQBP1,PRKCH,PYGO2,SMAD2,STAT5A,TBX5,TBX6,TGIF1,TLX2,TP53,VEGFB,XBP1 |
| GO.0051704 | multi-organism process | 27 | 0.0222 | ACVR2A,BAD,CCNB1,CDK16,FOXA3,GTF2A2,GTF2H1,HCFC2,HMGB2,HORMAD1,IFI16,IRF4,MAPK8,MECP2,NME5,PATZ1,PSME2,PYGO2,RAD23B,RPL32,SLC25A6,STAP1,STAT5A,STMN1,SUPT4H1,TP53,XBP1 |
| GO.0010629 | negative regulation of gene expression | 26 | 9.68E-05 | BIRC5,BIRC7,CCNB1,CCND1,DLX1,DR1,GTF2H1,HCFC2,HEXIM1,HMGB2,HNF1B,IFI16,JUNB,MEF2A,NR1I2,PATZ1,PBX1,RQCD1,SMAD2,SMARCE1,TBK1,TBX6,TGIF1,TP53,VEGFB,XBP1 |
| GO.0042127 | regulation of cell proliferation | 26 | 0.000156 | BAD,BIRC5,BIRC7,BTG3,CCNB1,CCND1,CDK2,CDKN2C,EZH2,HMGB2,JUNB,MAFG,MECP2,NDRG2,NFYA,PBX1,PHIP,PRKCH,PYGO2,RAC2,SERPINB5,STAT5A,TGIF1,TP53,VEGFB,XBP1 |
| GO.0010941 | regulation of cell death | 26 | 0.000162 | BAD,BIRC5,BIRC7,CASP7,DLX1,EEF1D,ELK1,FAF1,HMGB2,HNF1B,JUNB,KIT,MAPK8,MECP2,NFE2L2,NME5,PDPK1,PHIP,PRKCH,PSME2,STAT5A,STK10,TBX5,TP53,VEGFB,XBP1 |
| GO.0009888 | tissue development | 26 | 0.000366 | ACVR2A,CCNB1,CCND1,DLX3,EXT2,EZH2,EZR,HEYL,HRH2,JUNB,KIT,MEF2A,MEOX2,MSN,PBX1,PDPK1,SERPINB5,STAT5A,TGIF1,TLX2,TP53,TPM1,USH1C,WAS,ZBTB7B,ZFP36L1 |
| GO.0031399 | regulation of protein modification process | 26 | 0.000488 | ACVR2A,BIRC7,CBLC,CCND1,CDC25A,CDK2,CDKN2C,DSTYK,EZH2,FAF1,GTF2H1,HEXIM1,KIT,MAPK8,MECP2,NDRG2,PDPK1,PSME2,PYGO2,RAC2,STAP1,STK10,TBK1,TP53,VEGFB,XBP1 |
| GO.1902589 | single-organism organelle organization | 26 | 0.0103 | BAD,BIRC5,CCNB1,CDC25A,CDK2,CEP55,CHEK2,DR1,EZH2,FEN1,HMGB2,HORMAD1,IRF4,KIF9,KIT,MECP2,MEF2A,MTERF,NME5,PDPK1,PYGO2,RAC2,SMARCE1,STMN1,TPM1,USH1C |
| GO.0048585 | negative regulation of response to stimulus | 25 | 7.66E-05 | CBLC,CCND1,CTNNA2,DLX1,EZH2,FAF1,HEYL,HMGB2,IFI16,IRF4,NDRG2,NFE2L2,NLK,NME5,PHIP,PLD2,PSME2,RQCD1,SCFD1,SMAD2,STAP1,STUB1,TGIF1,TP53,XBP1 |
| GO.0044093 | positive regulation of molecular function | 25 | 0.00313 | BAD,BIRC7,CASP7,CCNB1,CCND1,DSTYK,EZH2,GTF2A2,HMGB2,HRH2,IFI16,IRF4,KIT,MAPK8,PDPK1,PRKCH,PSME2,PYGO2,RQCD1,STAP1,STK10,STUB1,TPM1,TRAF2,UBE2V1 |
| GO.0006366 | transcription from RNA polymerase II promoter | 24 | 3.80E-08 | CREB5,DLX3,ELK1,GMEB1,GTF2A2,GTF2H1,HEYL,HNF1B,IRF4,MAX,MEF2A,MEOX2,NFYA,NR1I2,PATZ1,PBX1,PRDM4,SMAD2,SUPT4H1,TGIF1,TP53,TTF2,XBP1,ZBTB7B |
| GO.0019220 | regulation of phosphate metabolic process | 24 | 0.00166 | ACVR2A,BAD,BIRC7,CBLC,CCNB1,CCND1,CDC25A,CDKN2C,DSTYK,EZH2,FAF1,GTF2H1,HEXIM1,HRH2,KIT,NDRG2,PDPK1,RAC2,STAP1,STK10,TBK1,TP53,VEGFB,XBP1 |
| GO.0009056 | catabolic process | 24 | 0.0118 | BAD,CBLC,CCNB1,CDK2,CHEK2,FAF1,FEN1,HMGB2,IFI16,IMPA1,MUTYH,NFE2L2,NR1I2,PLD2,PPP2CB,PSME2,RAD23B,RPL32,RQCD1,STUB1,TRAF2,XBP1,XYLB,ZFP36L1 |
| GO.0051726 | regulation of cell cycle | 23 | 5.14E-06 | BAD,BIRC5,BTG3,CCNB1,CCND1,CDC25A,CDK16,CDK18,CDK2,CDKN2C,CHEK2,EZH2,GTF2H1,HEXIM1,HORMAD1,JUNB,PBX1,PHIP,PRDM4,PSME2,STAT5A,STK10,TP53 |
| GO.0010648 | negative regulation of cell communication | 23 | 0.000145 | CBLC,CCND1,DLX1,EZH2,FAF1,HEYL,HMGB2,IRF4,NDRG2,NFE2L2,NLK,NME5,PHIP,PLD2,PSME2,RQCD1,SCFD1,SMAD2,STAP1,STUB1,TGIF1,TP53,XBP1 |
| GO.0042325 | regulation of phosphorylation | 23 | 0.000408 | ACVR2A,BAD,BIRC7,CBLC,CCNB1,CCND1,CDC25A,CDKN2C,DSTYK,EZH2,FAF1,GTF2H1,HEXIM1,KIT,NDRG2,PDPK1,RAC2,STAP1,STK10,TBK1,TP53,VEGFB,XBP1 |
| GO.0045595 | regulation of cell differentiation | 23 | 0.00297 | ACVR2A,CCND1,CTNNA2,DLX1,HEYL,HMGB2,HNF1B,IRF4,JUNB,MAFG,MEF2A,NFE2L2,NFYA,PQBP1,PRKCH,SMAD2,STAT5A,TBX5,TBX6,TGIF1,TLX2,TP53,XBP1 |
| GO.0033554 | cellular response to stress | 23 | 0.0106 | BAG3,BIRC7,CCNB1,CDK2,CHEK2,ELK1,EZH2,FEN1,FOXA3,GTF2H1,HMGB2,IFI16,MEF2A,MUTYH,NFE2L2,PPP2CB,PSME2,RAD23B,STK10,STUB1,TP53,TPM1,TXN2 |
| GO.0009605 | response to external stimulus | 23 | 0.0463 | BAG3,CCNB1,CCND1,CTNNA2,EZR,FOXA3,HMGB2,IFI16,IRF4,JUNB,KIT,MAPK8,MECP2,NFE2L2,PRDM4,RAC2,SLC25A6,STAP1,STMN1,TP53,TXN2,VEGFB,XBP1 |
| GO.0009790 | embryo development | 22 | 2.23E-05 | ACVR2A,CCNB1,DLX1,EXT2,HNF1B,HORMAD1,HOXB6,JUNB,KIT,MAFG,MEOX2,PBX1,PYGO2,SMAD2,TBX5,TBX6,TGIF1,TLX2,TP53,TPM1,USH1C,ZFP36L1 |
| GO.0009968 | negative regulation of signal transduction | 22 | 9.59E-05 | CBLC,CCND1,DLX1,EZH2,FAF1,HEYL,HMGB2,IRF4,NDRG2,NFE2L2,NLK,NME5,PHIP,PLD2,PSME2,RQCD1,SMAD2,STAP1,STUB1,TGIF1,TP53,XBP1 |
| GO.0001932 | regulation of protein phosphorylation | 22 | 0.000448 | ACVR2A,BIRC7,CBLC,CCNB1,CCND1,CDC25A,CDKN2C,DSTYK,EZH2,FAF1,GTF2H1,HEXIM1,KIT,NDRG2,PDPK1,RAC2,STAP1,STK10,TBK1,TP53,VEGFB,XBP1 |
| GO.0051240 | positive regulation of multicellular organismal process | 22 | 0.00252 | ACVR2A,CCNB1,CCND1,EZR,HEYL,HMGB2,HRH2,IFI16,IRF4,KIT,MAPK8,MECP2,NFE2L2,PRKCH,SMAD2,STAT5A,TBK1,TGIF1,TPM1,TRAF2,VEGFB,XBP1 |
| GO.0051247 | positive regulation of protein metabolic process | 22 | 0.00385 | ACVR2A,BAD,BIRC7,CASP7,CCND1,DSTYK,EZH2,EZR,IFI16,IRF4,KIT,MAPK8,MSN,PDPK1,PHIP,PSME2,STAP1,STAT5A,STK10,TBK1,TP53,VEGFB |
| GO.0009719 | response to endogenous stimulus | 22 | 0.00428 | ACVR2A,CCND1,CDK2,DSTYK,ELK1,EZH2,HEYL,HMGB2,JUNB,NLK,NR1I2,PDGFRL,PDPK1,PHIP,PPP2CB,SMAD2,STAT5A,STUB1,TGIF1,TP53,TXN2,XBP1 |
| GO.1901575 | organic substance catabolic process | 22 | 0.0103 | BAD,CBLC,CCNB1,CDK2,CHEK2,FAF1,FEN1,HMGB2,IMPA1,MUTYH,NFE2L2,PLD2,PPP2CB,PSME2,RAD23B,RPL32,RQCD1,STUB1,TRAF2,XBP1,XYLB,ZFP36L1 |
| GO.0006468 | protein phosphorylation | 21 | 6.08E-06 | BIRC5,BIRC7,CCNB1,CCND1,CDK16,CDK18,CDK2,CHEK2,DSTYK,ELK1,GTF2H1,MAPK8,MEF2A,PDGFRL,PDPK1,SMAD2,STAT4,STAT5A,STK10,STK38L,TBK1 |
| GO.0051338 | regulation of transferase activity | 21 | 2.69E-05 | BAD,BIRC7,CBLC,CCNB1,CCND1,CDC25A,CDK2,CDKN2C,DSTYK,EZH2,FAF1,GTF2H1,HEXIM1,KIT,PDPK1,PSME2,RAC2,STAP1,STK10,STUB1,TRAF2 |
| GO.0010558 | negative regulation of macromolecule biosynthetic process | 21 | 0.00579 | ASB1,BIRC5,CCND1,CHEK2,DLX1,DR1,HCFC2,HEXIM1,HMGB2,HNF1B,IFI16,JUNB,MEF2A,NR1I2,PATZ1,PBX1,SMAD2,SMARCE1,TBX6,TGIF1,XBP1 |
| GO.0002682 | regulation of immune system process | 21 | 0.00681 | ACVR2A,ELK1,HMGB2,IFI16,KIT,MAPK8,MEF2A,MSN,NFE2L2,PLD2,PRKCH,PSME2,SLC25A6,STAP1,STAT5A,STK10,TBK1,UBE2V1,VEGFB,WAS,XBP1 |
| GO.0043067 | regulation of programmed cell death | 21 | 0.0088 | BIRC5,BIRC7,CASP7,DLX1,HMGB2,HNF1B,KIT,MAPK8,MECP2,NFE2L2,NME5,PDPK1,PHIP,PRKCH,PSME2,STAT5A,STK10,TBX5,TP53,VEGFB,XBP1 |
| GO.0031327 | negative regulation of cellular biosynthetic process | 21 | 0.00951 | ASB1,BIRC5,CCND1,CHEK2,DLX1,DR1,HCFC2,HEXIM1,HMGB2,HNF1B,IFI16,JUNB,MEF2A,NR1I2,PATZ1,PBX1,SMAD2,SMARCE1,TBX6,TGIF1,XBP1 |
| GO.0022008 | neurogenesis | 21 | 0.00994 | CDK16,CDKN2C,CTNNA2,DLX1,EZH2,EZR,HEYL,HMGB2,KIT,MAPK8,MECP2,MEF2A,PBX1,PRKCH,RAC2,TBX6,TGIF1,TLX2,TP53,USH1C,XBP1 |
| GO.0048468 | cell development | 21 | 0.0396 | CCNB1,CDK16,CTNNA2,EZR,HEYL,HMGB2,HORMAD1,HRH2,MAPK8,MECP2,MEF2A,MSN,NME5,PBX1,PDPK1,PQBP1,PYGO2,RAC2,SMAD2,TPM1,USH1C |
| GO.0060429 | epithelium development | 20 | 0.000376 | CCND1,DLX3,EZR,HEYL,HRH2,KIT,MEOX2,MSN,PBX1,PDPK1,SERPINB5,SMAD2,STAT5A,TBX6,TGIF1,TP53,USH1C,WAS,XBP1,ZFP36L1 |
| GO.0045892 | negative regulation of transcription, DNA-templated | 20 | 0.00212 | BIRC5,CCND1,DLX1,DR1,HCFC2,HEXIM1,HMGB2,HNF1B,IFI16,JUNB,MEF2A,NR1I2,PATZ1,PBX1,SMAD2,SMARCE1,TBX6,TGIF1,TP53,XBP1 |
| GO.0022414 | reproductive process | 20 | 0.00355 | ACVR2A,ASB1,CCNB1,CCND1,CDK16,DLX3,FOXA3,HMGB2,HNF1B,HORMAD1,JUNB,NME5,PATZ1,PBX1,PYGO2,RAD23B,RQCD1,SERPINB5,STAT5A,ZFP36L1 |
| GO.2000113 | negative regulation of cellular macromolecule biosynthetic process | 20 | 0.00857 | BIRC5,CCND1,CHEK2,DLX1,DR1,HCFC2,HEXIM1,HMGB2,HNF1B,IFI16,JUNB,MEF2A,NR1I2,PATZ1,PBX1,SMAD2,SMARCE1,TBX6,TGIF1,XBP1 |
| GO.0045934 | negative regulation of nucleobase-containing compound metabolic process | 20 | 0.0118 | BIRC5,CCND1,CHEK2,DLX1,DR1,HCFC2,HEXIM1,HMGB2,HNF1B,IFI16,JUNB,MEF2A,NR1I2,PATZ1,PBX1,SMAD2,SMARCE1,TBX6,TGIF1,XBP1 |
| GO.0048699 | generation of neurons | 20 | 0.013 | CDK16,CTNNA2,DLX1,EZH2,EZR,HEYL,HMGB2,KIT,MAPK8,MECP2,MEF2A,PBX1,PRKCH,RAC2,TBX6,TGIF1,TLX2,TP53,USH1C,XBP1 |
| GO.0042981 | regulation of apoptotic process | 20 | 0.0189 | BIRC5,BIRC7,CASP7,DLX1,HMGB2,HNF1B,MAPK8,MECP2,NFE2L2,NME5,PDPK1,PHIP,PRKCH,PSME2,STAT5A,STK10,TBX5,TP53,VEGFB,XBP1 |
| GO.0071363 | cellular response to growth factor stimulus | 19 | 4.59E-06 | ACVR2A,BAD,DSTYK,ELK1,HEYL,JUNB,KIT,MAPK8,MEF2A,NLK,PDGFRL,PDPK1,PPP2CB,PRDM4,SMAD2,STUB1,TGIF1,TP53,XBP1 |
| GO.0043069 | negative regulation of programmed cell death | 19 | 0.000212 | BAD,BIRC5,BIRC7,DLX1,HMGB2,HNF1B,KIT,MAPK8,MECP2,NFE2L2,NME5,PDPK1,PHIP,PRKCH,PSME2,STAT5A,TP53,VEGFB,XBP1 |
| GO.0007167 | enzyme linked receptor protein signaling pathway | 19 | 0.000588 | ACVR2A,BAD,ELK1,JUNB,MAPK8,MEF2A,NLK,PDGFRL,PDPK1,PHIP,PPP2CB,PRDM4,SMAD2,STAP1,STAT5A,STUB1,TP53,VEGFB,XBP1 |
| GO.0071495 | cellular response to endogenous stimulus | 19 | 0.00182 | ACVR2A,BAD,CDK2,DSTYK,ELK1,HEYL,JUNB,NLK,NR1I2,PDGFRL,PDPK1,PHIP,PPP2CB,SMAD2,STAT5A,STUB1,TGIF1,TP53,XBP1 |
| GO.0051130 | positive regulation of cellular component organization | 19 | 0.00696 | BAD,BIRC5,CCNB1,EZR,FAF1,HNF1B,KIT,MAPK8,MECP2,PDPK1,PHIP,RAC2,SMAD2,STAP1,STUB1,TP53,TPM1,WAS,XBP1 |
| GO.0032270 | positive regulation of cellular protein metabolic process | 19 | 0.0228 | ACVR2A,BAD,BIRC7,CASP7,CCND1,DSTYK,EZH2,EZR,IFI16,KIT,MSN,PDPK1,PHIP,PSME2,STAP1,STK10,TBK1,TP53,VEGFB |
| GO.0009967 | positive regulation of signal transduction | 19 | 0.0235 | ACVR2A,BAD,BIRC7,DSTYK,EEF1D,EZH2,FAF1,HEXIM1,MAPK8,PHIP,PRKCH,PSME2,SMAD2,STAP1,TBK1,TP53,UBE2V1,VEGFB,XBP1 |
| GO.1902531 | regulation of intracellular signal transduction | 19 | 0.0397 | BAD,BIRC7,CBLC,DSTYK,EEF1D,EZH2,FAF1,HEXIM1,KIT,NDRG2,NFE2L2,NLK,NME5,PDPK1,RAC2,TBK1,UBE2V1,VEGFB,XBP1 |
| GO.0080134 | regulation of response to stress | 19 | 0.0482 | BAG3,BIRC7,ELK1,IFI16,MAPK8,MEF2A,NFE2L2,NME5,PDPK1,PSME2,SCFD1,SLC25A6,STAT5A,TBK1,TP53,TRAF2,UBE2V1,VEGFB,XBP1 |
| GO.0043085 | positive regulation of catalytic activity | 19 | 0.05 | BAD,BIRC7,CASP7,CCNB1,CCND1,DSTYK,EZH2,HMGB2,HRH2,IFI16,KIT,MAPK8,PDPK1,PSME2,STAP1,STK10,STUB1,TPM1,TRAF2 |
| GO.0008284 | positive regulation of cell proliferation | 18 | 0.000366 | BAD,BIRC5,CCNB1,CCND1,CDK2,HMGB2,KIT,MECP2,NFYA,PBX1,PHIP,PRKCH,RAC2,STAT5A,TBX5,TGIF1,VEGFB,XBP1 |
| GO.0002684 | positive regulation of immune system process | 18 | 0.000454 | ACVR2A,BAD,ELK1,HMGB2,IFI16,MAPK8,MEF2A,PLD2,PRKCH,PSME2,STAP1,STAT5A,TBK1,TRAF2,UBE2V1,VEGFB,WAS,XBP1 |
| GO.0043066 | negative regulation of apoptotic process | 18 | 0.000621 | BAD,BIRC5,BIRC7,DLX1,HMGB2,HNF1B,MAPK8,MECP2,NFE2L2,NME5,PDPK1,PHIP,PRKCH,PSME2,STAT5A,TP53,VEGFB,XBP1 |
| GO.0044702 | single organism reproductive process | 18 | 0.00714 | ACVR2A,ASB1,CCNB1,CCND1,CDK16,DLX3,FOXA3,HMGB2,HNF1B,HORMAD1,JUNB,NME5,PATZ1,PYGO2,RAD23B,SERPINB5,STAT5A,ZFP36L1 |
| GO.0051094 | positive regulation of developmental process | 18 | 0.0117 | ACVR2A,CCNB1,CCND1,CTNNA2,EZR,HEYL,HMGB2,JUNB,KIT,MAPK8,MECP2,MEF2A,PRKCH,SMAD2,STAT5A,TGIF1,VEGFB,XBP1 |
| GO.0009628 | response to abiotic stimulus | 18 | 0.0126 | BAD,CCNB1,CCND1,CDC25A,CDK2,CHEK2,EEF1D,ELK1,FEN1,HRH2,IFI16,JUNB,MAPK8,PDPK1,SCFD1,TP53,TXN2,XBP1 |
| GO.0008219 | cell death | 18 | 0.0135 | BIRC5,BIRC7,CASP7,CHEK2,DPF2,FAF1,HMGB2,IFI16,KIT,MEF2A,MEOX2,PDPK1,PPP2CB,PSME2,SLC25A6,TRAF2,XBP1,ZFP36L1 |
| GO.0003006 | developmental process involved in reproduction | 17 | 4.79E-05 | ACVR2A,ASB1,CCNB1,CCND1,DLX3,HMGB2,HNF1B,HORMAD1,JUNB,NME5,PATZ1,PBX1,PYGO2,RQCD1,SERPINB5,STAT5A,ZFP36L1 |
| GO.0043009 | chordate embryonic development | 17 | 7.42E-05 | ACVR2A,CCNB1,DLX1,HNF1B,HORMAD1,HOXB6,JUNB,MAFG,MEOX2,PBX1,PYGO2,SMAD2,TBX6,TGIF1,TP53,TPM1,ZFP36L1 |
| GO.0043549 | regulation of kinase activity | 17 | 0.00045 | BAD,BIRC7,CBLC,CCND1,CDC25A,CDKN2C,DSTYK,EZH2,FAF1,GTF2H1,HEXIM1,KIT,PDPK1,RAC2,STAP1,STK10,TRAF2 |
| GO.0050776 | regulation of immune response | 17 | 0.00124 | BAD,ELK1,IFI16,IRF4,KIT,MAPK8,MEF2A,PLD2,PSME2,RAC2,SLC25A6,STAT5A,TBK1,TRAF2,UBE2V1,WAS,XBP1 |
| GO.0009057 | macromolecule catabolic process | 17 | 0.00463 | CBLC,CCNB1,CDK2,CHEK2,FAF1,FEN1,HMGB2,NFE2L2,PPP2CB,PSME2,RAD23B,RPL32,RQCD1,STUB1,TRAF2,XBP1,ZFP36L1 |
| GO.0045937 | positive regulation of phosphate metabolic process | 17 | 0.00752 | ACVR2A,BAD,BIRC7,CCNB1,CCND1,DSTYK,EZH2,HRH2,KIT,PDPK1,PHIP,STAP1,STK10,TBK1,TP53,VEGFB,XBP1 |
| GO.0006915 | apoptotic process | 17 | 0.0209 | BIRC5,BIRC7,CASP7,CHEK2,DPF2,FAF1,HMGB2,IFI16,MAPK8,MEF2A,PDPK1,PPP2CB,PSME2,SLC25A6,TRAF2,XBP1,ZFP36L1 |
| GO.0012501 | programmed cell death | 17 | 0.0228 | BIRC5,BIRC7,CASP7,CHEK2,DPF2,FAF1,HMGB2,IFI16,KIT,MEF2A,PDPK1,PPP2CB,PSME2,SLC25A6,TRAF2,XBP1,ZFP36L1 |
| GO.0033043 | regulation of organelle organization | 17 | 0.0249 | BAD,BIRC5,CCNB1,EZR,HORMAD1,KIF9,MAPK8,MECP2,MSN,PHIP,PYGO2,RAC2,SCFD1,STAP1,TPM1,WAS,XBP1 |
| GO.0060284 | regulation of cell development | 16 | 0.00214 | BAD,DLX1,HEYL,HMGB2,HNF1B,KIT,NFYA,PBX1,PQBP1,PRKCH,SMAD2,TBX5,TBX6,TGIF1,TLX2,TP53 |
| GO.0000122 | negative regulation of transcription from RNA polymerase II promoter | 16 | 0.00223 | CBFA2T3,CCND1,DLX1,DR1,HCFC2,HEXIM1,HNF1B,IFI16,JUNB,MECP2,MEF2A,SMAD2,TBX6,TGIF1,TP53,XBP1 |
| GO.0044265 | cellular macromolecule catabolic process | 16 | 0.0027 | CBLC,CCNB1,CDK2,CHEK2,FAF1,FEN1,HMGB2,NFE2L2,PPP2CB,PSME2,RAD23B,RPL32,RQCD1,STUB1,XBP1,ZFP36L1 |
| GO.0042327 | positive regulation of phosphorylation | 16 | 0.00557 | ACVR2A,BAD,BIRC7,CCNB1,CCND1,DSTYK,EZH2,KIT,PDPK1,PHIP,STAP1,STK10,TBK1,TP53,VEGFB,XBP1 |
| GO.0051276 | chromosome organization | 16 | 0.00851 | CCNB1,CCND1,CDK2,DR1,EZH2,FEN1,FOXA3,HMGB2,HORMAD1,IRF4,MECP2,MTERF,RAD23B,SMARCE1,SUPT4H1,TP53 |
| GO.0048646 | anatomical structure formation involved in morphogenesis | 16 | 0.0243 | EXT2,HEYL,HNF1B,JUNB,MEF2A,MEOX2,NME5,PKNOX1,TBX5,TBX6,TGIF1,TLX2,TP53,TPM1,VEGFB,XBP1 |
| GO.0044087 | regulation of cellular component biogenesis | 15 | 0.00141 | EZR,FAF1,HNF1B,KIF9,KIT,MECP2,MSN,PRKCH,RAC2,SCFD1,STAP1,STMN1,STUB1,TP53,TPM1 |
| GO.0045859 | regulation of protein kinase activity | 15 | 0.00252 | BIRC7,CBLC,CCND1,CDC25A,CDKN2C,EZH2,FAF1,GTF2H1,HEXIM1,KIT,PDPK1,RAC2,STAP1,STK10,TRAF2 |
| GO.0009611 | response to wounding | 15 | 0.00667 | CCNB1,CDK2,EZH2,HBG1,KIF9,MAFG,PAPSS2,PDPK1,PRKCH,RAC2,TP53,TPM1,TXN2,VEGFB,WAS |
| GO.0001934 | positive regulation of protein phosphorylation | 15 | 0.0103 | ACVR2A,BIRC7,CCNB1,CCND1,DSTYK,EZH2,KIT,PDPK1,PHIP,STAP1,STK10,TBK1,TP53,VEGFB,XBP1 |
| GO.0042060 | wound healing | 14 | 0.00799 | CCNB1,CDK2,EZH2,HBG1,KIF9,MAFG,PAPSS2,PDPK1,PRKCH,RAC2,TP53,TPM1,VEGFB,WAS |
| GO.0072358 | cardiovascular system development | 14 | 0.0246 | CCNB1,DLX3,HEXIM1,HEYL,JUNB,MEF2A,MEOX2,PKNOX1,SMAD2,TBX5,TPM1,VEGFB,XBP1,ZFP36L1 |
| GO.0072359 | circulatory system development | 14 | 0.0246 | CCNB1,DLX3,HEXIM1,HEYL,JUNB,MEF2A,MEOX2,PKNOX1,SMAD2,TBX5,TPM1,VEGFB,XBP1,ZFP36L1 |
| GO.0009725 | response to hormone | 14 | 0.0458 | BAD,CCND1,CDK2,ELK1,EZH2,HMGB2,JUNB,KIT,NR1I2,PDPK1,PHIP,STAT5A,TXN2,XBP1 |
| GO.0042493 | response to drug | 13 | 0.000377 | BAD,CCNB1,CCND1,CDK2,HMGB2,HNF1B,JUNB,NR1I2,TGIF1,TP53,TXN2,VEGFB,XBP1 |
| GO.0016337 | single organismal cell-cell adhesion | 13 | 0.000488 | CTNNA2,EZR,IRF4,KIT,MSN,PATZ1,PKNOX1,RAC2,STAT5A,STK10,TP53,WAS,ZFP36L1 |
| GO.0007346 | regulation of mitotic cell cycle | 13 | 0.000893 | BIRC5,BTG3,CCNB1,CCND1,CDK2,CHEK2,EZH2,PBX1,PHIP,PSME2,STAT5A,STK10,TP53 |
| GO.0050778 | positive regulation of immune response | 13 | 0.00423 | ELK1,IFI16,MAPK8,MEF2A,PDPK1,PLD2,PSME2,STAT5A,TBK1,TRAF2,UBE2V1,WAS,XBP1 |
| GO.0048598 | embryonic morphogenesis | 13 | 0.00499 | ACVR2A,EXT2,HNF1B,HOXB6,PBX1,SMAD2,TBX5,TBX6,TGIF1,TLX2,TP53,USH1C,ZFP36L1 |
| GO.0051347 | positive regulation of transferase activity | 13 | 0.0053 | BAD,BIRC7,CCNB1,CCND1,DSTYK,EZH2,KIT,PDPK1,PSME2,STAP1,STK10,STUB1,TRAF2 |
| GO.0008283 | cell proliferation | 13 | 0.0197 | ACVR2A,BAD,CBFA2T3,CCND1,CDC25A,HNF1B,IFI16,JUNB,PRDM4,STAT4,TBK1,XBP1,ZFP36L1 |
| GO.0051960 | regulation of nervous system development | 13 | 0.02 | DLX1,EZH2,HEYL,HMGB2,KIT,MECP2,PBX1,PQBP1,PRKCH,TBX6,TGIF1,TLX2,TP53 |
| GO.0050878 | regulation of body fluid levels | 13 | 0.0204 | CCND1,CDK2,HBG1,KIF9,MAFG,PAPSS2,PDPK1,PRKCH,RAC2,STAT5A,TP53,VEGFB,WAS |
| GO.0006974 | cellular response to DNA damage stimulus | 13 | 0.0228 | BAD,CCND1,CDK2,CHEK2,FEN1,GTF2H1,HMGB2,IFI16,MUTYH,PSME2,RAD23B,STUB1,TP53 |
| GO.0007169 | transmembrane receptor protein tyrosine kinase signaling pathway | 13 | 0.0243 | BAD,ELK1,MAPK8,MEF2A,PDGFRL,PDPK1,PHIP,PPP2CB,PRDM4,STAP1,STAT5A,VEGFB,XBP1 |
| GO.0007159 | leukocyte cell-cell adhesion | 12 | 2.25E-05 | EZR,IRF4,KIT,MSN,PATZ1,PKNOX1,RAC2,STAT5A,STK10,TP53,WAS,ZFP36L1 |
| GO.0048608 | reproductive structure development | 12 | 0.00142 | ACVR2A,ASB1,CCND1,DLX3,HMGB2,HNF1B,JUNB,KIT,PATZ1,SERPINB5,STAT5A,ZFP36L1 |
| GO.0061458 | reproductive system development | 12 | 0.00156 | ACVR2A,ASB1,CCND1,DLX3,HMGB2,HNF1B,JUNB,KIT,PATZ1,SERPINB5,STAT5A,ZFP36L1 |
| GO.0009314 | response to radiation | 12 | 0.00333 | CCND1,CDC25A,CHEK2,EEF1D,ELK1,FEN1,HRH2,IFI16,JUNB,KIT,MAPK8,TP53 |
| GO.0030163 | protein catabolic process | 12 | 0.0127 | CBLC,CCNB1,CDK2,CHEK2,FAF1,NFE2L2,PPP2CB,PSME2,RAD23B,STUB1,TRAF2,XBP1 |
| GO.0048729 | tissue morphogenesis | 12 | 0.0184 | EXT2,HEYL,HNF1B,PBX1,SERPINB5,SMAD2,STAT5A,TBX5,TBX6,TGIF1,TLX2,TPM1 |
| GO.0050767 | regulation of neurogenesis | 12 | 0.0194 | DLX1,EZH2,HEYL,HMGB2,KIT,PBX1,PQBP1,PRKCH,TBX6,TGIF1,TLX2,TP53 |
| GO.0001775 | cell activation | 12 | 0.0308 | KIT,PATZ1,PDPK1,PKNOX1,PRKCH,RAC2,STAT5A,TBK1,TP53,VEGFB,WAS,ZFP36L1 |
| GO.0006325 | chromatin organization | 12 | 0.0345 | CCNB1,CCND1,CDK2,DR1,EZH2,FOXA3,HMGB2,IRF4,MECP2,SMARCE1,SUPT4H1,TP53 |
| GO.0007420 | brain development | 12 | 0.0396 | BAG3,CKB,CTNNA2,DLX1,EZH2,HNF1B,MECP2,NDRG2,NME5,PYGO2,STMN1,TP53 |
| GO.0045596 | negative regulation of cell differentiation | 12 | 0.0419 | CCND1,DLX1,EZH2,HNF1B,NFE2L2,SMAD2,STAT5A,TBX5,TBX6,TLX2,TP53,XBP1 |
| GO.0006979 | response to oxidative stress | 11 | 0.00138 | BAD,CDK2,EZH2,MAPK8,NFE2L2,PPP2CB,TP53,TPM1,TRAF2,TXN2,XBP1 |
| GO.0007389 | pattern specification process | 11 | 0.00645 | ACVR2A,DLX1,HNF1B,HOXB6,MEOX2,PBX1,SMAD2,TBX5,TBX6,TGIF1,TP53 |
| GO.0010035 | response to inorganic substance | 11 | 0.00667 | ANXA11,BAD,CCNB1,CCND1,EZH2,JUNB,MAPK8,MEF2A,NFE2L2,PPP2CB,TRAF2 |
| GO.0045786 | negative regulation of cell cycle | 11 | 0.0101 | BTG3,CCND1,CDK2,CDKN2C,CHEK2,EZH2,HEXIM1,HORMAD1,PRDM4,PSME2,TP53 |
| GO.0002764 | immune response-regulating signaling pathway | 11 | 0.0145 | BAD,ELK1,KIT,MAPK8,MEF2A,PDPK1,PLD2,PSME2,TBK1,UBE2V1,WAS |
| GO.0044257 | cellular protein catabolic process | 11 | 0.021 | CBLC,CCNB1,CDK2,CHEK2,FAF1,NFE2L2,PPP2CB,PSME2,RAD23B,STUB1,XBP1 |
| GO.0010564 | regulation of cell cycle process | 11 | 0.028 | BIRC5,CCNB1,CCND1,CDK2,CHEK2,EZH2,HORMAD1,PBX1,PHIP,PSME2,TP53 |
| GO.0007596 | blood coagulation | 11 | 0.0312 | CDK2,HBG1,KIF9,MAFG,PAPSS2,PDPK1,PRKCH,RAC2,TP53,VEGFB,WAS |
| GO.0071593 | lymphocyte aggregation | 10 | 0.000264 | IRF4,KIT,PATZ1,PKNOX1,RAC2,STAT5A,STK10,TP53,WAS,ZFP36L1 |
| GO.0007548 | sex differentiation | 10 | 0.000659 | ACVR2A,ASB1,CCND1,HMGB2,HNF1B,KIT,PATZ1,PBX1,RQCD1,STAT5A |
| GO.0071496 | cellular response to external stimulus | 10 | 0.00194 | BAD,BAG3,FOXA3,IFI16,MAPK8,NFE2L2,PRDM4,TP53,TXN2,XBP1 |
| GO.0003002 | regionalization | 10 | 0.00423 | ACVR2A,DLX1,HNF1B,HOXB6,MEOX2,PBX1,SMAD2,TBX6,TGIF1,TP53 |
| GO.0001701 | in utero embryonic development | 10 | 0.0093 | CCNB1,HNF1B,HORMAD1,JUNB,MAFG,PYGO2,SMAD2,TP53,TPM1,ZFP36L1 |
| GO.0031349 | positive regulation of defense response | 10 | 0.00965 | ELK1,IFI16,MAPK8,MEF2A,PDPK1,PSME2,SLC25A6,STAT5A,TBK1,UBE2V1 |
| GO.0002253 | activation of immune response | 10 | 0.0225 | ELK1,IFI16,MAPK8,MEF2A,PDPK1,PLD2,PSME2,TBK1,UBE2V1,WAS |
| GO.0071900 | regulation of protein serine/threonine kinase activity | 10 | 0.0243 | BIRC7,CBLC,CCND1,CDC25A,CDKN2C,EZH2,GTF2H1,HEXIM1,KIT,TRAF2 |
| GO.0006511 | ubiquitin-dependent protein catabolic process | 10 | 0.0254 | CBLC,CCNB1,CDK2,FAF1,NFE2L2,PPP2CB,PSME2,RAD23B,STUB1,XBP1 |
| GO.0033674 | positive regulation of kinase activity | 10 | 0.0341 | BAD,BIRC7,CCND1,DSTYK,EZH2,KIT,PDPK1,STAP1,STK10,TRAF2 |
| GO.0034599 | cellular response to oxidative stress | 9 | 0.000212 | CDK2,EZH2,MAPK8,NFE2L2,TP53,TPM1,TRAF2,TXN2,XBP1 |
| GO.0000302 | response to reactive oxygen species | 9 | 0.00033 | BAD,CDK2,EZH2,MAPK8,NFE2L2,PPP2CB,TPM1,TRAF2,TXN2 |
| GO.1901987 | regulation of cell cycle phase transition | 9 | 0.00521 | BIRC5,CCNB1,CCND1,CDK2,CHEK2,EZH2,PBX1,PSME2,TP53 |
| GO.0002521 | leukocyte differentiation | 9 | 0.00714 | CBFA2T3,IFI16,JUNB,KIT,PATZ1,PKNOX1,STAT5A,TP53,ZFP36L1 |
| GO.0045089 | positive regulation of innate immune response | 9 | 0.00714 | ELK1,IFI16,MAPK8,MEF2A,PDPK1,PSME2,STAT5A,TBK1,UBE2V1 |
| GO.0045787 | positive regulation of cell cycle | 9 | 0.012 | BIRC5,CCNB1,CCND1,CDK2,PBX1,PHIP,PSME2,STAT5A,TP53 |
| GO.0044772 | mitotic cell cycle phase transition | 9 | 0.0135 | BIRC5,CCNB1,CCND1,CDC25A,CDK2,CDKN2C,CHEK2,PSME2,USH1C |
| GO.0046649 | lymphocyte activation | 9 | 0.0148 | IRF4,KIT,PATZ1,PKNOX1,STAT5A,TBK1,TP53,WAS,ZFP36L1 |
| GO.0002757 | immune response-activating signal transduction | 9 | 0.0343 | ELK1,MAPK8,MEF2A,PDPK1,PLD2,PSME2,TBK1,UBE2V1,WAS |
| GO.2001233 | regulation of apoptotic signaling pathway | 9 | 0.0386 | BAD,FAF1,HMGB2,MAPK8,NFE2L2,NME5,PHIP,TRAF2,XBP1 |
| GO.0009952 | anterior/posterior pattern specification | 8 | 0.00458 | ACVR2A,HNF1B,HOXB6,MEOX2,PBX1,SMAD2,TBX6,TP53 |
| GO.0042110 | T cell activation | 8 | 0.00696 | IRF4,KIT,PATZ1,PKNOX1,STAT5A,TP53,WAS,ZFP36L1 |
| GO.0002218 | activation of innate immune response | 8 | 0.0129 | ELK1,IFI16,MAPK8,MEF2A,PDPK1,PSME2,TBK1,UBE2V1 |
| GO.0090068 | positive regulation of cell cycle process | 8 | 0.0132 | BIRC5,CCNB1,CCND1,CDK2,PBX1,PHIP,PSME2,TP53 |
| GO.0023014 | signal transduction by protein phosphorylation | 8 | 0.0138 | ACVR2A,BIRC7,ELK1,KIT,MAPK8,MEF2A,NLK,STK10 |
| GO.1901990 | regulation of mitotic cell cycle phase transition | 8 | 0.0144 | BIRC5,CCNB1,CCND1,CDK2,EZH2,PBX1,PSME2,TP53 |
| GO.0006367 | transcription initiation from RNA polymerase II promoter | 8 | 0.0153 | GTF2A2,GTF2H1,JUNB,NR1I2,SMAD2,TBX5,TGIF1,TP53 |
| GO.0071214 | cellular response to abiotic stimulus | 8 | 0.0177 | BAD,BAG3,CDC25A,EEF1D,ELK1,IFI16,MAPK8,TP53 |
| GO.0051098 | regulation of binding | 8 | 0.0184 | HMGB2,IFI16,IRF4,MAPK8,PYGO2,SMAD2,STUB1,TRAF2 |
| GO.0010038 | response to metal ion | 8 | 0.0294 | ANXA11,BAD,CCNB1,CCND1,CDK2,JUNB,MAPK8,MEF2A |
| GO.0043161 | proteasome-mediated ubiquitin-dependent protein catabolic process | 8 | 0.0383 | CCNB1,CDK2,FAF1,NFE2L2,PPP2CB,PSME2,RAD23B,STUB1 |
| GO.1903829 | positive regulation of cellular protein localization | 8 | 0.0445 | BAD,CCNB1,MAPK8,MSN,PDPK1,RAC2,TP53,XBP1 |
| GO.0030217 | T cell differentiation | 7 | 0.00166 | IRF4,KIT,PATZ1,PKNOX1,STAT5A,TP53,ZFP36L1 |
| GO.0071241 | cellular response to inorganic substance | 7 | 0.00276 | BAD,CCNB1,CDK2,JUNB,MAPK8,MEF2A,TRAF2 |
| GO.0046661 | male sex differentiation | 7 | 0.00373 | ACVR2A,ASB1,CCND1,HMGB2,KIT,PATZ1,STAT5A |
| GO.0007369 | gastrulation | 7 | 0.00579 | ACVR2A,EXT2,HNF1B,SMAD2,TBX6,TLX2,TP53 |
| GO.0031669 | cellular response to nutrient levels | 7 | 0.0149 | FOXA3,IFI16,NFE2L2,PRDM4,TP53,TXN2,XBP1 |
| GO.0000075 | cell cycle checkpoint | 7 | 0.0312 | BIRC5,CCND1,CDK2,CHEK2,HORMAD1,PSME2,TP53 |
| GO.0045930 | negative regulation of mitotic cell cycle | 7 | 0.0319 | BTG3,CCND1,CDK2,CHEK2,EZH2,PSME2,TP53 |
| GO.0038093 | Fc receptor signaling pathway | 7 | 0.0343 | BAD,KIT,MAPK8,PDPK1,PLD2,UBE2V1,WAS |
| GO.0090287 | regulation of cellular response to growth factor stimulus | 7 | 0.0386 | ACVR2A,DSTYK,PDPK1,SMAD2,STUB1,TP53,VEGFB |
| GO.0010948 | negative regulation of cell cycle process | 7 | 0.0396 | CCND1,CDK2,CHEK2,EZH2,HORMAD1,PSME2,TP53 |
| GO.1901214 | regulation of neuron death | 7 | 0.0399 | BAD,DLX1,ELK1,MECP2,TP53,TRAF2,VEGFB |
| GO.0002758 | innate immune response-activating signal transduction | 7 | 0.0417 | ELK1,MAPK8,MEF2A,PDPK1,PSME2,TBK1,UBE2V1 |
| GO.0050863 | regulation of T cell activation | 7 | 0.0458 | BAD,IRF4,PDPK1,RAC2,STAT5A,TRAF2,XBP1 |
| GO.0050673 | epithelial cell proliferation | 6 | 0.00194 | ACVR2A,BAD,CCND1,HNF1B,KIT,XBP1 |
| GO.0034614 | cellular response to reactive oxygen species | 6 | 0.0027 | CDK2,EZH2,MAPK8,NFE2L2,TPM1,TRAF2 |
| GO.0045931 | positive regulation of mitotic cell cycle | 6 | 0.0072 | BIRC5,CCNB1,CCND1,PBX1,PHIP,STAT5A |
| GO.2000736 | regulation of stem cell differentiation | 6 | 0.0072 | EZH2,HNF1B,NFE2L2,NFYA,SMAD2,TBX5 |
| GO.0030856 | regulation of epithelial cell differentiation | 6 | 0.00752 | BAD,CCND1,EZH2,MAFG,PRKCH,STAT5A |
| GO.0010212 | response to ionizing radiation | 6 | 0.0151 | CCND1,CHEK2,EEF1D,ELK1,IFI16,TP53 |
| GO.0007179 | transforming growth factor beta receptor signaling pathway | 6 | 0.0168 | JUNB,NLK,SMAD2,STUB1,TGIF1,TP53 |
| GO.0002703 | regulation of leukocyte mediated immunity | 6 | 0.018 | PDPK1,RAC2,STAP1,STAT5A,TRAF2,WAS |
| GO.0048017 | inositol lipid-mediated signaling | 6 | 0.0191 | BAD,EZR,KIT,PDPK1,PLD2,XBP1 |
| GO.0000086 | G2/M transition of mitotic cell cycle | 6 | 0.0208 | BIRC5,CCNB1,CDC25A,CDK2,CHEK2,USH1C |
| GO.0002699 | positive regulation of immune effector process | 6 | 0.0208 | PDPK1,RAC2,STAP1,STAT5A,TRAF2,XBP1 |
| GO.0055123 | digestive system development | 6 | 0.0239 | CCNB1,HNF1B,HRH2,KIT,SMAD2,XBP1 |
| GO.0009636 | response to toxic substance | 6 | 0.0332 | CCNB1,CDK2,PPP2CB,SCFD1,TP53,XBP1 |
| GO.0097193 | intrinsic apoptotic signaling pathway | 6 | 0.0341 | CASP7,CHEK2,IFI16,MAPK8,TRAF2,XBP1 |
| GO.1901988 | negative regulation of cell cycle phase transition | 6 | 0.04 | CCND1,CDK2,CHEK2,EZH2,PSME2,TP53 |
| GO.0000082 | G1/S transition of mitotic cell cycle | 6 | 0.0432 | CCNB1,CCND1,CDC25A,CDK2,CDKN2C,PSME2 |
| GO.0071560 | cellular response to transforming growth factor beta stimulus | 6 | 0.0486 | JUNB,NLK,SMAD2,STUB1,TGIF1,TP53 |
| GO.0031016 | pancreas development | 5 | 0.0124 | FOXA3,HNF1B,PDPK1,SMAD2,XBP1 |
| GO.0000079 | regulation of cyclin-dependent protein serine/threonine kinase activity | 5 | 0.0178 | CCND1,CDC25A,CDKN2C,GTF2H1,HEXIM1 |
| GO.0002573 | myeloid leukocyte differentiation | 5 | 0.0231 | CBFA2T3,IFI16,IRF4,JUNB,KIT |
| GO.0034142 | toll-like receptor 4 signaling pathway | 5 | 0.0246 | ELK1,MAPK8,MEF2A,TBK1,UBE2V1 |
| GO.0042542 | response to hydrogen peroxide | 5 | 0.0254 | BAD,EZH2,MAPK8,NFE2L2,PPP2CB |
| GO.0044773 | mitotic DNA damage checkpoint | 5 | 0.0254 | CCND1,CDK2,CHEK2,PSME2,TP53 |
| GO.2000134 | negative regulation of G1/S transition of mitotic cell cycle | 5 | 0.0297 | CCND1,CDK2,EZH2,PSME2,TP53 |
| GO.0051592 | response to calcium ion | 5 | 0.0386 | ANXA11,BAD,CCND1,JUNB,MEF2A |
| GO.0031056 | regulation of histone modification | 5 | 0.0445 | CCNB1,MAPK8,MECP2,PYGO2,XBP1 |
| GO.0008584 | male gonad development | 5 | 0.0468 | ACVR2A,CCND1,HMGB2,KIT,PATZ1 |
| GO.0046546 | development of primary male sexual characteristics | 5 | 0.0468 | ACVR2A,CCND1,HMGB2,KIT,PATZ1 |
| GO.0042149 | cellular response to glucose starvation | 4 | 0.00268 | IFI16,NFE2L2,TP53,XBP1 |
| GO.0071479 | cellular response to ionizing radiation | 4 | 0.0164 | EEF1D,ELK1,IFI16,TP53 |
| GO.0001707 | mesoderm formation | 4 | 0.0332 | EXT2,SMAD2,TBX6,TLX2 |
| GO.0034146 | toll-like receptor 5 signaling pathway | 4 | 0.0396 | ELK1,MAPK8,MEF2A,UBE2V1 |
| GO.0034166 | toll-like receptor 10 signaling pathway | 4 | 0.0396 | ELK1,MAPK8,MEF2A,UBE2V1 |
| GO.0048332 | mesoderm morphogenesis | 4 | 0.0396 | EXT2,SMAD2,TBX6,TLX2 |
| GO.1902402 | signal transduction involved in mitotic DNA damage checkpoint | 4 | 0.0453 | CDK2,CHEK2,PSME2,TP53 |
| GO.0071732 | cellular response to nitric oxide | 3 | 0.00609 | CDK2,MAPK8,TRAF2 |
| GO.0045579 | positive regulation of B cell differentiation | 3 | 0.0072 | BAD,STAT5A,XBP1 |
| GO.0010662 | regulation of striated muscle cell apoptotic process | 3 | 0.02 | BAG3,PDPK1,TP53 |
| GO.0032924 | activin receptor signaling pathway | 3 | 0.0243 | ACVR2A,SMAD2,TGIF1 |
| GO.1900740 | positive regulation of protein insertion into mitochondrial membrane involved in apoptotic signaling pathway | 3 | 0.0323 | BAD,MAPK8,TP53 |
| GO.0006984 | ER-nucleus signaling pathway | 3 | 0.0473 | NFE2L2,TP53,XBP1 |
| GO.0097300 | programmed necrotic cell death | 3 | 0.0473 | MAPK8,TP53,TRAF2 |
| GO.0022614 | membrane to membrane docking | 2 | 0.023 | EZR,MSN |
| GO.1902966 | positive regulation of protein localization to early endosome | 2 | 0.023 | EZR,MSN |
| GO.0050861 | positive regulation of B cell receptor signaling pathway | 2 | 0.0312 | PRKCH,STAP1 |
| GO.0034351 | negative regulation of glial cell apoptotic process | 2 | 0.0396 | PRKCH,TRAF2 |
| GO.0060753 | regulation of mast cell chemotaxis | 2 | 0.0396 | RAC2,VEGFB |
| GO.2000643 | positive regulation of early endosome to late endosome transport | 2 | 0.0396 | EZR,MSN |
| GO.0000103 | sulfate assimilation | 2 | 0.0483 | PAPSS2,TXN2 |
| GO.0032927 | positive regulation of activin receptor signaling pathway | 2 | 0.0483 | ACVR2A,SMAD2 |
| GO.0071499 | cellular response to laminar fluid shear stress | 2 | 0.0483 | NFE2L2,XBP1 |
