# Supplementary material for: A diagnostic autoantibody signature for primary cutaneous melanoma
Source: Oncotarget. 2018 Jul 17;9(55):30539–51. doi: 10.18632/oncotarget.25669 (PMC6078131; doi:10.18632/oncotarget.25669)
Supplement: Supplementary file 6 [file oncotarget-09-30539-s006.docx]

| **#pathway ID** | **pathway description** | **observed gene count** | **false discovery rate** | **matching proteins in your network (labels)** |
| --- | --- | --- | --- | --- |
| GO.0005488 | binding | 92 | 0.000253 | ACVR2A,AK2,BAD,BIRC5,CBFA2T3,CBLC,CCNB1,CCND1,CDC25A,CDK16,CDK18,CDK2,CDKN2C,CHEK2,CKB,CREB5,DLX1,DLX3,DR1,DSTYK,EEF1D,EXT2,EZR,FAF1,FOXR2,GMEB1,GTF2A2,HBG1,HEXIM1,HMGB2,HSFY1,IFI16,IMPA1,INPP1,IRF4,JUNB,KIF9,KIT,LRRFIP2,MAFG,MAPK8,MAX,MEF2A,MEOX2,MSN,MTERF,MUTYH,NFE2L2,NFYA,NLK,NME5,PAPSS2,PDPK1,PHIP,PKNOX1,PLD2,PPP2CB,PQBP1,PRDM4,PRKCH,PYGO2,RAC2,RPL32,RQCD1,SCAND1,SCFD1,SMAD2,SMARCE1,STAP1,STAT4,STAT5A,STK10,STK38L,STMN1,STUB1,TBX5,TBX6,TLX2,TP53,TPM1,TRAF2,TTF2,UBE2V1,USH1C,VEGFB,WAS,XBP1,XYLB,ZBTB7B,ZNF169,ZNF444,ZNF449 |
| GO.1901363 | heterocyclic compound binding | 64 | 1.27E-05 | ACVR2A,AK2,ANXA11,CDK16,CDK18,CDK2,CHEK2,CKB,CREB5,DLX1,DLX3,DPF2,DSTYK,EEF1D,EZR,FEN1,FOXR2,GMEB1,HBG1,HEXIM1,HMGB2,HSFY1,IFI16,IRF4,JUNB,KIF9,KIT,MAFG,MAPK8,MAX,MEF2A,MEOX2,MSN,MTERF,NFYA,NLK,NME5,NR1I2,PAPSS2,PDPK1,PKNOX1,PQBP1,PRDM4,PRKCH,RAC2,RPL32,SMAD2,SMARCE1,STAT4,STAT5A,STK10,STK38L,TBK1,TBX6,TGIF1,TLX2,TP53,TTF2,XBP1,XYLB,ZBTB7B,ZNF169,ZNF444,ZNF449 |
| GO.0097159 | organic cyclic compound binding | 64 | 1.92E-05 | ACVR2A,AK2,ANXA11,CDK16,CDK18,CDK2,CHEK2,CKB,CREB5,DLX1,DLX3,DPF2,DSTYK,EEF1D,EZR,FEN1,FOXR2,GMEB1,HBG1,HEXIM1,HMGB2,HSFY1,IFI16,IRF4,JUNB,KIF9,KIT,MAFG,MAPK8,MAX,MEF2A,MEOX2,MSN,MTERF,NFYA,NLK,NME5,NR1I2,PAPSS2,PDPK1,PKNOX1,PQBP1,PRDM4,PRKCH,RAC2,RPL32,SMAD2,SMARCE1,STAT4,STAT5A,STK10,STK38L,TBK1,TBX6,TGIF1,TLX2,TP53,TTF2,XBP1,XYLB,ZBTB7B,ZNF169,ZNF444,ZNF449 |
| GO.0005515 | protein binding | 62 | 8.71E-08 | ANXA11,BAD,BIRC5,BIRC7,CBLC,CCNB1,CCND1,CDC25A,CDK2,CDKN2C,CHEK2,CKB,DR1,EEF1D,EXT2,EZR,FAF1,GTF2A2,HEYL,HMGB2,HNF1B,IFI16,IMPA1,IRF4,JUNB,KIF9,KIT,LRRFIP2,MAPK8,MECP2,MEF2A,MSN,MUTYH,NFE2L2,NLK,PBX1,PDPK1,PHIP,PPP2CB,PRKCH,RAD23B,RQCD1,SCAND1,SCFD1,SMAD2,SMARCE1,STAP1,STK10,STMN1,STUB1,SUPT4H1,TBK1,TBX5,TBX6,TP53,TPM1,TRAF2,UBE2V1,USH1C,VEGFB,WAS,XBP1 |
| GO.0003676 | nucleic acid binding | 43 | 0.0044 | ANXA11,CREB5,DLX1,DLX3,DPF2,EEF1D,EZR,FEN1,FOXR2,GMEB1,HEXIM1,HMGB2,HSFY1,IFI16,IRF4,JUNB,MAFG,MAX,MEF2A,MEOX2,MSN,MTERF,NFYA,NR1I2,PKNOX1,PQBP1,PRDM4,RPL32,SMAD2,SMARCE1,STAT4,STAT5A,TBK1,TBX6,TGIF1,TLX2,TP53,TTF2,XBP1,ZBTB7B,ZNF169,ZNF444,ZNF449 |
| GO.0003677 | DNA binding | 40 | 7.92E-07 | CREB5,DLX1,DLX3,DPF2,EEF1D,FEN1,FOXR2,GMEB1,HMGB2,HOXB6,HSFY1,IFI16,IRF4,JUNB,MAFG,MAX,MECP2,MEF2A,MEOX2,MTERF,NFYA,NR1I2,PKNOX1,PQBP1,PRDM4,SMAD2,SMARCE1,STAT4,STAT5A,TBX6,TGIF1,TLX2,TP53,TTF2,XBP1,ZBTB7B,ZFP36L1,ZNF169,ZNF444,ZNF449 |
| GO.0043565 | sequence-specific DNA binding | 35 | 2.57E-15 | CREB5,DLX1,DLX3,DPF2,DR1,ELK1,EZH2,FOXA3,FOXR2,GMEB1,HMGB2,HNF1B,HOXB6,HSFY1,IFI16,IRF4,JUNB,KLF12,MAFG,MAX,MECP2,MEF2A,MEOX2,NFYA,NR1I2,PATZ1,PBX1,PKNOX1,SMAD2,STAT4,STAT5A,TGIF1,TLX2,XBP1,ZBTB7B |
| GO.0003700 | transcription factor activity, sequence-specific DNA binding | 29 | 5.43E-09 | CBFA2T3,CREB5,ELK1,FOXA3,FOXR2,GMEB1,HMGB2,HOXB6,HSFY1,IFI16,IRF4,MAFG,MECP2,MEF2A,MEOX2,NFYA,NR1I2,PATZ1,SCAND1,SMAD2,STAT4,STAT5A,SUPT4H1,TBX6,TGIF1,TP53,ZFP36L1,ZNF444,ZNF449 |
| GO.0016740 | transferase activity | 29 | 0.00231 | AK2,BIRC5,BIRC7,CBLC,CCNB1,CCND1,CDK16,CDK18,CDK2,CHEK2,CKB,DSTYK,EXT2,EZH2,IRF4,MAPK8,NLK,NME5,PAPSS2,PDGFRL,PDPK1,PRDM4,SMARCE1,STK10,STK38L,STUB1,TBK1,TRAF2,XYLB |
| GO.0044212 | transcription regulatory region DNA binding | 27 | 5.57E-12 | DLX1,DPF2,EZH2,FOXA3,GMEB1,HEYL,HMGB2,HNF1B,IFI16,IRF4,JUNB,KLF12,MAX,MEF2A,MEOX2,NFYA,NR1I2,PATZ1,PBX1,SMAD2,STAT4,STAT5A,TBX5,TGIF1,TP53,XBP1,ZBTB7B |
| GO.0000977 | RNA polymerase II regulatory region sequence-specific DNA binding | 24 | 5.57E-12 | DLX1,DPF2,ELK1,GMEB1,HEYL,IFI16,IRF4,JUNB,KLF12,MAX,MEF2A,MEOX2,NFE2L2,NR1I2,PATZ1,PBX1,SMAD2,STAT4,STAT5A,TBX5,TGIF1,TP53,XBP1,ZBTB7B |
| GO.0000976 | transcription regulatory region sequence-specific DNA binding | 24 | 3.01E-11 | DLX1,DPF2,ELK1,GMEB1,HEYL,HMGB2,IFI16,IRF4,JUNB,KLF12,MAX,MEF2A,MEOX2,NFYA,NR1I2,PATZ1,PBX1,SMAD2,STAT4,STAT5A,TBX5,TGIF1,XBP1,ZBTB7B |
| GO.0019899 | enzyme binding | 24 | 0.000445 | BAD,BIRC5,BIRC7,CCNB1,CCND1,CDC25A,CDKN2C,CHEK2,CKB,FAF1,MAPK8,MEF2A,MSN,NLK,PDPK1,PRKCH,SMAD2,STAP1,STUB1,TP53,TRAF2,UBE2V1,WAS,XBP1 |
| GO.0000981 | RNA polymerase II transcription factor activity, sequence-specific DNA binding | 23 | 9.11E-10 | DLX3,ELK1,FOXA3,FOXR2,GMEB1,HNF1B,IFI16,IRF4,KLF12,MAX,MEOX2,NFE2L2,NR1I2,PATZ1,PBX1,PKNOX1,SCAND1,SMAD2,TBX5,TGIF1,TP53,XBP1,ZNF444 |
| GO.0005524 | ATP binding | 22 | 0.0185 | ACVR2A,AK2,CDK16,CDK18,CDK2,CHEK2,CKB,DSTYK,KIF9,KIT,MAPK8,NLK,NME5,PAPSS2,PDPK1,PRKCH,STK10,STK38L,TBK1,TP53,TTF2,XYLB |
| GO.0044877 | macromolecular complex binding | 21 | 0.000602 | ANXA11,BIRC5,CBFA2T3,DLX1,DLX3,ELK1,EZH2,EZR,HMGB2,MECP2,MEF2A,MUTYH,PATZ1,PHIP,PQBP1,PYGO2,SMAD2,SMARCE1,TGIF1,TP53,XBP1 |
| GO.0016301 | kinase activity | 19 | 5.15E-05 | AK2,CCNB1,CCND1,CDK16,CDK18,CDK2,CHEK2,CKB,DSTYK,MAPK8,NLK,NME5,PAPSS2,PDGFRL,PDPK1,STK10,STK38L,TBK1,XYLB |
| GO.0008134 | transcription factor binding | 18 | 5.59E-08 | CCND1,DR1,EEF1D,FAF1,GTF2A2,HEYL,HMGB2,IFI16,IRF4,JUNB,MEF2A,NFE2L2,NLK,PBX1,SMAD2,TBX5,TBX6,TP53 |
| GO.0000989 | transcription factor activity, transcription factor binding | 17 | 3.49E-05 | CBFA2T3,CCND1,DR1,GMEB1,GTF2A2,HCFC2,JUNB,KLF12,MAX,MECP2,MEF2A,NR1I2,PQBP1,SCAND1,SMARCE1,TBX6,TGIF1 |
| GO.0046983 | protein dimerization activity | 17 | 0.000362 | BIRC5,CHEK2,EXT2,GTF2A2,HEYL,HNF1B,IMPA1,KIF9,KIT,MEF2A,PPP2CB,RQCD1,STK10,STUB1,SUPT4H1,TP53,XBP1 |
| GO.0001228 | transcriptional activator activity, RNA polymerase II transcription regulatory region sequence-specific binding | 16 | 7.87E-08 | DLX3,ELK1,GMEB1,HEYL,IRF4,JUNB,MEF2A,MEOX2,NFE2L2,NR1I2,PATZ1,PBX1,PKNOX1,SMAD2,TBX5,TP53 |
| GO.0003712 | transcription cofactor activity | 16 | 6.62E-05 | CBFA2T3,CCND1,DR1,GMEB1,GTF2A2,HCFC2,JUNB,KLF12,MAX,MECP2,MEF2A,NR1I2,PQBP1,SCAND1,SMARCE1,TGIF1 |
| GO.0016773 | phosphotransferase activity, alcohol group as acceptor | 16 | 0.000809 | CCNB1,CCND1,CDK16,CDK18,CDK2,CHEK2,DSTYK,MAPK8,NLK,PAPSS2,PDGFRL,PDPK1,STK10,STK38L,TBK1,XYLB |
| GO.0003682 | chromatin binding | 15 | 8.73E-05 | CBFA2T3,DLX1,DLX3,ELK1,EZH2,HMGB2,MECP2,MEF2A,PATZ1,PYGO2,SMAD2,SMARCE1,TGIF1,TP53,XBP1 |
| GO.0001159 | core promoter proximal region DNA binding | 14 | 2.92E-06 | DPF2,ELK1,GMEB1,HNF1B,IFI16,IRF4,JUNB,MAX,MEF2A,MEOX2,PATZ1,PBX1,SMAD2,TGIF1 |
| GO.0000982 | transcription factor activity, RNA polymerase II core promoter proximal region sequence-specific binding | 14 | 6.92E-06 | DLX3,ELK1,GMEB1,HEYL,IFI16,IRF4,JUNB,MEF2A,MEOX2,PATZ1,PBX1,SMAD2,TGIF1,TP53 |
| GO.0019900 | kinase binding | 14 | 0.000106 | BAD,CCNB1,CCND1,CDC25A,CDKN2C,CHEK2,FAF1,MSN,STAP1,STUB1,TP53,TRAF2,WAS,XBP1 |
| GO.0004672 | protein kinase activity | 14 | 0.00215 | CCNB1,CCND1,CDK16,CDK18,CDK2,CHEK2,DSTYK,MAPK8,NLK,PDGFRL,PDPK1,STK10,STK38L,TBK1 |
| GO.0000978 | RNA polymerase II core promoter proximal region sequence-specific DNA binding | 13 | 1.07E-05 | DPF2,ELK1,GMEB1,IFI16,IRF4,JUNB,MAX,MEF2A,MEOX2,PATZ1,PBX1,SMAD2,TGIF1 |
| GO.0019901 | protein kinase binding | 13 | 0.000157 | BAD,CCNB1,CCND1,CDC25A,CDKN2C,CHEK2,FAF1,MSN,STAP1,TP53,TRAF2,WAS,XBP1 |
| GO.0001077 | transcriptional activator activity, RNA polymerase II core promoter proximal region sequence-specific binding | 12 | 8.19E-06 | DLX3,ELK1,GMEB1,HEYL,IRF4,JUNB,MEF2A,MEOX2,PATZ1,PBX1,SMAD2,TP53 |
| GO.0043566 | structure-specific DNA binding | 11 | 4.22E-05 | ELK1,EZH2,FEN1,HMGB2,IFI16,MECP2,MTERF,RAD23B,SMAD2,TP53,XBP1 |
| GO.0004674 | protein serine/threonine kinase activity | 11 | 0.00887 | CDK16,CDK18,CDK2,CHEK2,DSTYK,MAPK8,NLK,PDPK1,STK10,STK38L,TBK1 |
| GO.0031625 | ubiquitin protein ligase binding | 10 | 0.000197 | CHEK2,CKB,FAF1,NLK,SMAD2,STUB1,TP53,TRAF2,UBE2V1,XBP1 |
| GO.0003713 | transcription coactivator activity | 10 | 0.00297 | GMEB1,GTF2A2,HCFC2,JUNB,MAX,MEF2A,NR1I2,PQBP1,SCAND1,SMARCE1 |
| GO.0046982 | protein heterodimerization activity | 9 | 0.00182 | BIRC5,EXT2,GTF2A2,HEYL,MEF2A,PPP2CB,SUPT4H1,TP53,XBP1 |
| GO.0019904 | protein domain specific binding | 9 | 0.047 | CBLC,HEYL,LRRFIP2,MECP2,NFE2L2,NLK,RQCD1,STUB1,WAS |
| GO.0003690 | double-stranded DNA binding | 8 | 6.71E-05 | ELK1,FEN1,HMGB2,IFI16,MECP2,MTERF,SMAD2,TP53 |
| GO.0001047 | core promoter binding | 7 | 0.00237 | ELK1,EZH2,HEYL,IFI16,NFYA,STAT5A,XBP1 |
| GO.0003714 | transcription corepressor activity | 7 | 0.0468 | CBFA2T3,CCND1,DR1,JUNB,KLF12,MECP2,TGIF1 |
| GO.0005057 | receptor signaling protein activity | 6 | 0.0168 | ACVR2A,KIT,MAPK8,NLK,SMAD2,STK10 |
| GO.0033613 | activating transcription factor binding | 5 | 0.00224 | EEF1D,MEF2A,NFE2L2,SMAD2,TBX6 |
| GO.0035326 | enhancer binding | 5 | 0.0122 | HMGB2,MEOX2,NFE2L2,SMAD2,XBP1 |
| GO.0003705 | transcription factor activity, RNA polymerase II distal enhancer sequence-specific binding | 5 | 0.032 | HNF1B,MEF2A,MEOX2,NFE2L2,PKNOX1 |
| GO.0003684 | damaged DNA binding | 4 | 0.047 | FEN1,HMGB2,RAD23B,TP53 |
| GO.0016538 | cyclin-dependent protein serine/threonine kinase regulator activity | 3 | 0.0295 | CCND1,CDKN2C,HEXIM1 |
| GO.0035033 | histone deacetylase regulator activity | 2 | 0.0345 | MAPK8,TP53 |
