# Supplementary material for: A diagnostic autoantibody signature for primary cutaneous melanoma
Source: Oncotarget. 2018 Jul 17;9(55):30539–51. doi: 10.18632/oncotarget.25669 (PMC6078131; doi:10.18632/oncotarget.25669)
Supplement: Supplementary file 7 [file oncotarget-09-30539-s007.docx]

| **#pathway ID** | **pathway description** | **observed gene count** | **false discovery rate** | **matching proteins in your network (labels)** |
| --- | --- | --- | --- | --- |
| GO.0044464 | cell part | 105 | 0.0433 | ACVR2A,ANXA11,ASB1,BAD,BIRC5,BIRC7,BTG3,CASP7,CBFA2T3,CBLC,CCNB1,CCND1,CDC25A,CDKN2C,CEP55,CHEK2,CREB5,CWC27,DLX1,DLX3,DR1,DSTYK,EEF1D,EXT2,EZR,FAF1,FEN1,FMR1NB,FOXA3,FOXR2,GMEB1,GTF2A2,GTF2H1,HCFC2,HEXIM1,HEYL,HMGB2,HOXB6,HRH2,HSFY1,IFI16,IMPA1,INPP1,JUNB,KIF9,KLF12,KLK3,MAFG,MAPK8,MAX,MEF2A,MEOX2,MLANA,MSN,MTERF,NDRG2,NFE2L2,NFYA,NLK,NR1I2,PAPSS2,PATZ1,PDPK1,PHIP,PKNOX1,PRDM4,PRKCH,PTPN20A,PYGO2,RAD23B,RPL32,RQCD1,SCAND1,SERPINB5,SLC25A6,SLCO6A1,SMAD2,SMARCE1,STAP1,STAT4,STAT5A,STK10,STK38L,STUB1,SUPT4H1,TBK1,TBX5,TBX6,TGIF1,TLX2,TP53,TPM1,TRAF2,TTF2,TXN2,UBE2V1,VEGFB,WAS,XBP1,XYLB,ZBTB7B,ZFP36L1,ZNF169,ZNF444,ZNF449 |
| GO.0005623 | cell | 105 | 0.0466 | ACVR2A,ANXA11,ASB1,BAD,BIRC5,BIRC7,BTG3,CASP7,CBFA2T3,CBLC,CCNB1,CCND1,CDC25A,CDKN2C,CEP55,CHEK2,CREB5,CWC27,DLX1,DLX3,DR1,DSTYK,EEF1D,EXT2,EZR,FAF1,FEN1,FMR1NB,FOXA3,FOXR2,GMEB1,GTF2A2,GTF2H1,HCFC2,HEXIM1,HEYL,HMGB2,HOXB6,HRH2,HSFY1,IFI16,IMPA1,INPP1,JUNB,KIF9,KLF12,KLK3,MAFG,MAPK8,MAX,MEF2A,MEOX2,MLANA,MSN,MTERF,NDRG2,NFE2L2,NFYA,NLK,NR1I2,PAPSS2,PATZ1,PDPK1,PHIP,PKNOX1,PRDM4,PRKCH,PTPN20A,PYGO2,RAD23B,RPL32,RQCD1,SCAND1,SERPINB5,SLC25A6,SLCO6A1,SMAD2,SMARCE1,STAP1,STAT4,STAT5A,STK10,STK38L,STUB1,SUPT4H1,TBK1,TBX5,TBX6,TGIF1,TLX2,TP53,TPM1,TRAF2,TTF2,TXN2,UBE2V1,VEGFB,WAS,XBP1,XYLB,ZBTB7B,ZFP36L1,ZNF169,ZNF444,ZNF449 |
| GO.0005622 | intracellular | 103 | 0.00213 | ACVR2A,ANXA11,ASB1,BAD,BIRC5,BIRC7,BTG3,CASP7,CBFA2T3,CBLC,CCNB1,CCND1,CDC25A,CDKN2C,CEP55,CHEK2,CREB5,CWC27,DLX1,DLX3,DR1,DSTYK,EEF1D,EXT2,FAF1,FEN1,FMR1NB,FOXA3,FOXR2,GMEB1,GTF2A2,GTF2H1,HCFC2,HEXIM1,HEYL,HMGB2,HOXB6,HSFY1,IFI16,IMPA1,INPP1,JUNB,KIF9,KLF12,KLK3,MAFG,MAPK8,MAX,MEF2A,MEOX2,MLANA,MSN,MTERF,NDRG2,NFE2L2,NFYA,NLK,NME5,NR1I2,PAPSS2,PATZ1,PHIP,PKNOX1,PLD2,PRDM4,PRKCH,PTPN20A,PYGO2,RAD23B,RPL32,RQCD1,SCAND1,SERPINB5,SLC25A6,SMAD2,SMARCE1,STAP1,STAT4,STAT5A,STK10,STK38L,STUB1,SUPT4H1,TBK1,TBX5,TBX6,TGIF1,TLX2,TP53,TPM1,TTF2,TXN2,UBE2V1,USH1C,VEGFB,WAS,XBP1,XYLB,ZBTB7B,ZFP36L1,ZNF169,ZNF444,ZNF449 |
| GO.0044424 | intracellular part | 101 | 0.00214 | ACVR2A,ANXA11,BAD,BIRC5,BIRC7,BTG3,CASP7,CBFA2T3,CBLC,CCNB1,CCND1,CDC25A,CDKN2C,CEP55,CHEK2,CREB5,CWC27,DLX1,DLX3,DR1,DSTYK,EEF1D,EXT2,FAF1,FEN1,FMR1NB,FOXA3,FOXR2,GMEB1,GTF2A2,GTF2H1,HCFC2,HEXIM1,HEYL,HMGB2,HOXB6,HSFY1,IFI16,IMPA1,INPP1,JUNB,KIF9,KLF12,KLK3,MAFG,MAPK8,MAX,MEF2A,MEOX2,MLANA,MSN,MTERF,NDRG2,NFE2L2,NFYA,NLK,NR1I2,PAPSS2,PATZ1,PHIP,PKNOX1,PLD2,PRDM4,PRKCH,PTPN20A,PYGO2,RAD23B,RPL32,RQCD1,SCAND1,SERPINB5,SLC25A6,SMAD2,SMARCE1,STAP1,STAT4,STAT5A,STK10,STK38L,STUB1,SUPT4H1,TBK1,TBX5,TBX6,TGIF1,TLX2,TP53,TPM1,TTF2,TXN2,UBE2V1,USH1C,VEGFB,WAS,XBP1,XYLB,ZBTB7B,ZFP36L1,ZNF169,ZNF444,ZNF449 |
| GO.0043229 | intracellular organelle | 99 | 3.62E-05 | ANXA11,BAD,BAG3,BIRC5,BIRC7,CASP7,CBFA2T3,CBLC,CCNB1,CCND1,CDC25A,CDKN2C,CEP55,CHEK2,CKB,CREB5,CTNNA2,CWC27,DLX1,DLX3,DR1,EEF1D,EXT2,FAF1,FEN1,FMR1NB,FOXA3,FOXR2,GMEB1,GTF2A2,GTF2H1,HCFC2,HEXIM1,HEYL,HMGB2,HNF1B,HOXB6,HSFY1,IFI16,JUNB,KIF9,KLF12,KLK3,MAFG,MAPK8,MAX,MEF2A,MEOX2,MLANA,MSN,MTERF,NDRG2,NFYA,NLK,NR1I2,PATZ1,PBX1,PHIP,PKNOX1,PLD2,PPP2CB,PRDM4,PSME2,PTPN20A,PYGO2,RAD23B,RPL32,RQCD1,SCAND1,SLC25A6,SMAD2,SMARCE1,STAP1,STAT4,STAT5A,STK38L,STMN1,STUB1,SUPT4H1,TBK1,TBX5,TBX6,TGIF1,TLX2,TP53,TPM1,TRAF2,TTF2,TXN2,UBE2V1,USH1C,VEGFB,WAS,XBP1,ZBTB7B,ZFP36L1,ZNF169,ZNF444,ZNF449 |
| GO.0043226 | organelle | 99 | 0.00156 | ANXA11,BAD,BAG3,BIRC5,BIRC7,CASP7,CBFA2T3,CCNB1,CCND1,CDC25A,CDKN2C,CEP55,CHEK2,CREB5,CTNNA2,CWC27,DLX1,DLX3,DR1,EEF1D,EXT2,FAF1,FEN1,FMR1NB,FOXA3,FOXR2,GMEB1,GTF2A2,GTF2H1,HCFC2,HEXIM1,HEYL,HMGB2,HNF1B,HOXB6,HSFY1,IFI16,IMPA1,JUNB,KIF9,KLF12,KLK3,MAFG,MAPK8,MAX,MEF2A,MEOX2,MLANA,MSN,MTERF,NDRG2,NFYA,NLK,NME5,NR1I2,PATZ1,PBX1,PHIP,PKNOX1,PLD2,PRDM4,PRKCH,PSME2,PTPN20A,PYGO2,RAD23B,RPL32,RQCD1,SCAND1,SERPINB5,SLC25A6,SMAD2,SMARCE1,STAP1,STAT4,STAT5A,STK10,STK38L,STMN1,STUB1,SUPT4H1,TBK1,TBX5,TBX6,TGIF1,TLX2,TP53,TPM1,TTF2,TXN2,UBE2V1,VEGFB,XBP1,XYLB,ZBTB7B,ZFP36L1,ZNF169,ZNF444,ZNF449 |
| GO.0043227 | membrane-bounded organelle | 95 | 0.00213 | ANXA11,BAD,BIRC5,BIRC7,CASP7,CBFA2T3,CCNB1,CCND1,CDC25A,CDK16,CDK2,CDKN2C,CHEK2,CREB5,CWC27,DLX1,DLX3,DR1,EEF1D,EXT2,EZR,FAF1,FEN1,FMR1NB,FOXA3,FOXR2,GMEB1,GTF2A2,GTF2H1,HCFC2,HEXIM1,HEYL,HMGB2,HNF1B,HOXB6,HSFY1,IFI16,IMPA1,JUNB,KLF12,KLK3,MAFG,MAPK8,MAX,MEF2A,MEOX2,MLANA,MSN,MTERF,NDRG2,NFYA,NLK,NR1I2,PATZ1,PBX1,PHIP,PKNOX1,PLD2,PQBP1,PRDM4,PRKCH,PSME2,PTPN20A,PYGO2,RAD23B,RQCD1,SCAND1,SERPINB5,SLC25A6,SMAD2,SMARCE1,STAP1,STAT4,STAT5A,STK10,STMN1,STUB1,SUPT4H1,TBK1,TBX5,TBX6,TGIF1,TLX2,TP53,TTF2,TXN2,UBE2V1,VEGFB,XBP1,XYLB,ZBTB7B,ZFP36L1,ZNF169,ZNF444,ZNF449 |
| GO.0043231 | intracellular membrane-bounded organelle | 93 | 0.000103 | ANXA11,BAD,BIRC5,BIRC7,CASP7,CBFA2T3,CBLC,CCNB1,CCND1,CDC25A,CDK16,CDK2,CDKN2C,CHEK2,CKB,CREB5,CWC27,DLX1,DLX3,DR1,EEF1D,EXT2,EZR,FAF1,FEN1,FMR1NB,FOXA3,FOXR2,GMEB1,GTF2A2,GTF2H1,HCFC2,HEXIM1,HEYL,HMGB2,HNF1B,HOXB6,HSFY1,IFI16,JUNB,KLF12,KLK3,MAFG,MAPK8,MAX,MEF2A,MEOX2,MLANA,MSN,MTERF,NDRG2,NFYA,NLK,NR1I2,PATZ1,PBX1,PHIP,PKNOX1,PLD2,PPP2CB,PQBP1,PRDM4,PSME2,PTPN20A,PYGO2,RAD23B,RQCD1,SCAND1,SLC25A6,SMAD2,SMARCE1,STAP1,STAT4,STAT5A,STUB1,SUPT4H1,TBK1,TBX5,TBX6,TGIF1,TLX2,TP53,TRAF2,TTF2,TXN2,UBE2V1,VEGFB,XBP1,ZBTB7B,ZFP36L1,ZNF169,ZNF444,ZNF449 |
| GO.0005634 | nucleus | 88 | 2.54E-12 | ANXA11,BIRC5,BIRC7,CASP7,CBFA2T3,CBLC,CCNB1,CCND1,CDC25A,CDK2,CDKN2C,CHEK2,CREB5,CWC27,DLX1,DLX3,DR1,EEF1D,ELK1,EZR,FEN1,FMR1NB,FOXA3,FOXR2,GMEB1,GTF2A2,GTF2H1,HCFC2,HEXIM1,HEYL,HMGB2,HNF1B,HOXB6,HSFY1,IFI16,JUNB,KLF12,KLK3,MAFG,MAPK8,MAX,MECP2,MEF2A,MEOX2,MSN,MUTYH,NDRG2,NFE2L2,NFYA,NLK,NR1I2,PATZ1,PBX1,PDPK1,PHIP,PKNOX1,PPP2CB,PQBP1,PRDM4,PSME2,PTPN20A,PYGO2,RAC2,RAD23B,RQCD1,SCAND1,SLC25A6,SMAD2,SMARCE1,STAP1,STAT4,STAT5A,STUB1,SUPT4H1,TBX5,TBX6,TGIF1,TLX2,TP53,TTF2,TXN2,UBE2V1,XBP1,ZBTB7B,ZFP36L1,ZNF169,ZNF444,ZNF449 |
| GO.0005737 | cytoplasm | 83 | 0.0157 | ACVR2A,ANXA11,BAD,BIRC5,BIRC7,BTG3,CASP7,CBFA2T3,CCNB1,CCND1,CDC25A,CDK2,CDKN2C,CEP55,CHEK2,CTNNA2,DPF2,DSTYK,EEF1D,ELK1,EXT2,EZH2,EZR,FAF1,FEN1,GMEB1,HCFC2,HEXIM1,HEYL,HMGB2,HSFY1,IFI16,IMPA1,INPP1,IRF4,KIF9,MAX,MEF2A,MEOX2,MLANA,MSN,MTERF,MUTYH,NDRG2,NFE2L2,NLK,PAPSS2,PATZ1,PBX1,PKNOX1,PLD2,PPP2CB,PRDM4,PRKCH,PSME2,PTPN20A,RAC2,RAD23B,RPL32,RQCD1,SERPINB5,SLC25A6,SMAD2,STAP1,STAT4,STAT5A,STK10,STK38L,STUB1,TBK1,TBX5,TLX2,TP53,TPM1,TTF2,TXN2,UBE2V1,USH1C,VEGFB,WAS,XBP1,XYLB,ZFP36L1 |
| GO.0044422 | organelle part | 75 | 0.000396 | ANXA11,BAD,BAG3,BIRC5,CASP7,CBFA2T3,CCNB1,CCND1,CDC25A,CEP55,CHEK2,CWC27,DR1,EEF1D,ELK1,EXT2,EZH2,FAF1,FEN1,FMR1NB,GMEB1,GTF2A2,GTF2H1,HCFC2,HEXIM1,HEYL,HMGB2,HNF1B,HORMAD1,IFI16,JUNB,KIF9,MAFG,MAPK8,MAX,MECP2,MEF2A,MEOX2,MLANA,MTERF,MUTYH,NDRG2,NFYA,NLK,NR1I2,PATZ1,PBX1,PDPK1,PLD2,PPP2CB,PSME2,PTPN20A,PYGO2,RAD23B,RPL32,SLC25A6,SMAD2,SMARCE1,STAT4,STAT5A,STMN1,STUB1,SUPT4H1,TBK1,TBX5,TGIF1,TP53,TPM1,TTF2,TXN2,USH1C,VEGFB,WAS,XBP1,ZBTB7B |
| GO.0044446 | intracellular organelle part | 73 | 0.000644 | ANXA11,BAD,BIRC5,CASP7,CBFA2T3,CCNB1,CCND1,CDC25A,CEP55,CHEK2,CWC27,DR1,EEF1D,ELK1,EXT2,EZH2,FAF1,FEN1,FMR1NB,GMEB1,GTF2A2,GTF2H1,HCFC2,HEXIM1,HEYL,HMGB2,HNF1B,HORMAD1,IFI16,JUNB,KIF9,MAFG,MAPK8,MAX,MECP2,MEF2A,MEOX2,MLANA,MTERF,MUTYH,NDRG2,NFYA,NLK,NR1I2,PATZ1,PBX1,PDPK1,PLD2,PPP2CB,PSME2,PTPN20A,PYGO2,RAD23B,RPL32,SLC25A6,SMAD2,SMARCE1,STAT4,STAT5A,STMN1,STUB1,SUPT4H1,TBK1,TBX5,TGIF1,TP53,TPM1,TRAF2,TTF2,TXN2,VEGFB,XBP1,ZBTB7B |
| GO.0031974 | membrane-enclosed lumen | 63 | 3.76E-10 | AK2,ANXA11,BIRC5,CASP7,CBFA2T3,CCNB1,CCND1,CDC25A,CDK2,CHEK2,DPF2,DR1,EEF1D,ELK1,EZH2,EZR,FEN1,FMR1NB,GMEB1,GTF2A2,GTF2H1,HCFC2,HEXIM1,HEYL,HMGB2,HNF1B,HORMAD1,IFI16,JUNB,MAFG,MAPK8,MAX,MECP2,MEF2A,MEOX2,MTERF,MUTYH,NDRG2,NFE2L2,NFYA,NLK,NR1I2,PATZ1,PBX1,PDPK1,PQBP1,PSME2,PYGO2,RAD23B,SMAD2,SMARCE1,STAT4,STAT5A,STUB1,SUPT4H1,TBX5,TGIF1,TP53,TTF2,TXN2,VEGFB,XBP1,ZBTB7B |
| GO.0044428 | nuclear part | 62 | 5.97E-12 | ANXA11,BIRC5,CASP7,CBFA2T3,CCNB1,CCND1,CDC25A,CDK2,CHEK2,CWC27,DR1,EEF1D,ELK1,EZH2,EZR,FAF1,FEN1,FMR1NB,GMEB1,GTF2A2,GTF2H1,HCFC2,HEXIM1,HEYL,HMGB2,HNF1B,HORMAD1,IFI16,JUNB,MAFG,MAPK8,MAX,MECP2,MEF2A,MEOX2,MUTYH,NDRG2,NFE2L2,NFYA,NLK,NR1I2,PATZ1,PBX1,PDPK1,PQBP1,PSME2,PYGO2,RAC2,RAD23B,SMAD2,SMARCE1,STAT4,STAT5A,STUB1,SUPT4H1,TBX5,TGIF1,TP53,TTF2,TXN2,XBP1,ZBTB7B |
| GO.0043233 | organelle lumen | 62 | 6.17E-10 | ANXA11,BIRC5,CASP7,CBFA2T3,CCNB1,CCND1,CDC25A,CDK2,CHEK2,DPF2,DR1,EEF1D,ELK1,EZH2,EZR,FEN1,FMR1NB,GMEB1,GTF2A2,GTF2H1,HCFC2,HEXIM1,HEYL,HMGB2,HNF1B,HORMAD1,IFI16,JUNB,MAFG,MAPK8,MAX,MECP2,MEF2A,MEOX2,MTERF,MUTYH,NDRG2,NFE2L2,NFYA,NLK,NR1I2,PATZ1,PBX1,PDPK1,PQBP1,PSME2,PYGO2,RAD23B,SMAD2,SMARCE1,STAT4,STAT5A,STUB1,SUPT4H1,TBX5,TGIF1,TP53,TTF2,TXN2,VEGFB,XBP1,ZBTB7B |
| GO.0070013 | intracellular organelle lumen | 61 | 9.51E-10 | ANXA11,BIRC5,CASP7,CBFA2T3,CCNB1,CCND1,CDC25A,CDK2,CHEK2,DPF2,DR1,EEF1D,ELK1,EZH2,EZR,FEN1,FMR1NB,GMEB1,GTF2A2,GTF2H1,HCFC2,HEXIM1,HEYL,HMGB2,HNF1B,HORMAD1,IFI16,JUNB,MAFG,MAPK8,MAX,MECP2,MEF2A,MEOX2,MTERF,MUTYH,NDRG2,NFE2L2,NFYA,NLK,NR1I2,PATZ1,PBX1,PDPK1,PQBP1,PSME2,PYGO2,RAD23B,SMAD2,SMARCE1,STAT4,STAT5A,STUB1,SUPT4H1,TBX5,TGIF1,TP53,TTF2,TXN2,XBP1,ZBTB7B |
| GO.0031981 | nuclear lumen | 60 | 2.54E-12 | ANXA11,BIRC5,CASP7,CBFA2T3,CCNB1,CCND1,CDC25A,CDK2,CHEK2,DPF2,DR1,EEF1D,ELK1,EZH2,EZR,FEN1,FMR1NB,GMEB1,GTF2A2,GTF2H1,HCFC2,HEXIM1,HEYL,HMGB2,HNF1B,HORMAD1,IFI16,JUNB,MAFG,MAPK8,MAX,MECP2,MEF2A,MEOX2,MUTYH,NDRG2,NFE2L2,NFYA,NLK,NR1I2,PATZ1,PBX1,PDPK1,PQBP1,PSME2,PYGO2,RAD23B,SMAD2,SMARCE1,STAT4,STAT5A,STUB1,SUPT4H1,TBX5,TGIF1,TP53,TTF2,TXN2,XBP1,ZBTB7B |
| GO.0005654 | nucleoplasm | 55 | 2.54E-12 | ANXA11,CASP7,CBFA2T3,CCNB1,CCND1,CDC25A,CDK2,CHEK2,DPF2,DR1,ELK1,EZH2,FEN1,GMEB1,GTF2A2,GTF2H1,HCFC2,HEXIM1,HEYL,HMGB2,HNF1B,IFI16,IRF4,JUNB,MAFG,MAPK8,MAX,MECP2,MEF2A,MEOX2,MUTYH,NDRG2,NFE2L2,NFYA,NLK,NR1I2,PATZ1,PBX1,PDPK1,PQBP1,PSME2,PYGO2,RAD23B,SMAD2,SMARCE1,STAT4,STAT5A,STUB1,SUPT4H1,TBX5,TGIF1,TP53,TTF2,XBP1,ZBTB7B |
| GO.0005829 | cytosol | 44 | 5.12E-05 | BAD,BAG3,BIRC5,CASP7,CCNB1,CCND1,CDC25A,CDK2,CDKN2C,CKB,CTNNA2,EEF1D,EZR,FAF1,IFI16,IMPA1,INPP1,IRF4,MAPK8,MECP2,NDRG2,NFE2L2,NLK,PAPSS2,PDPK1,PPP2CB,PRKCH,PSME2,RAC2,RPL32,RQCD1,SMAD2,STAT5A,STMN1,STUB1,TBK1,TP53,TPM1,TRAF2,UBE2V1,USH1C,WAS,XBP1,ZFP36L1 |
| GO.0043234 | protein complex | 40 | 0.0466 | ACVR2A,BIRC5,CCNB1,CCND1,DPF2,DR1,EEF1D,EXT2,EZH2,EZR,FAF1,FEN1,GTF2A2,HBG1,HMGB2,HNF1B,IRF4,JUNB,KIF9,MAX,MEF2A,NFYA,PBX1,PKNOX1,PTPN20A,RAD23B,RQCD1,SCFD1,SLC25A6,SMAD2,SMARCE1,STAP1,STMN1,STUB1,SUPT4H1,TP53,TPM1,TRAF2,TTF2,UBE2V1 |
| GO.0043232 | intracellular non-membrane-bounded organelle | 38 | 0.0292 | ANXA11,BAG3,BIRC5,CBFA2T3,CCNB1,CDK16,CEP55,CTNNA2,DPF2,EEF1D,EZH2,FEN1,FMR1NB,HMGB2,IFI16,IRF4,JUNB,KIF9,MECP2,MEF2A,MSN,MTERF,NDRG2,PPP2CB,PQBP1,PTPN20A,RPL32,RQCD1,SMAD2,SMARCE1,STK38L,STMN1,STUB1,TP53,TPM1,TXN2,USH1C,WAS |
| GO.0044427 | chromosomal part | 16 | 0.000644 | BIRC5,CCNB1,CDK2,DPF2,EZH2,HMGB2,HORMAD1,IRF4,JUNB,MECP2,MEF2A,NFE2L2,PPP2CB,SMAD2,SMARCE1,TP53 |
| GO.0015630 | microtubule cytoskeleton | 16 | 0.0377 | ANXA11,BIRC5,CCNB1,CDK16,CDK2,CEP55,DPF2,EZR,KIF9,NDRG2,NFE2L2,PPP2CB,PQBP1,PTPN20A,RPL32,STMN1 |
| GO.0005694 | chromosome | 15 | 0.00509 | BIRC5,CCNB1,CDK2,DPF2,EZH2,HMGB2,IRF4,JUNB,MECP2,MEF2A,NFE2L2,PPP2CB,SMAD2,SMARCE1,TP53 |
| GO.0044451 | nucleoplasm part | 13 | 0.0239 | CDK2,CHEK2,DR1,EZH2,GTF2A2,GTF2H1,IFI16,MAX,MEOX2,PQBP1,SUPT4H1,TP53,TTF2 |
| GO.0000228 | nuclear chromosome | 12 | 0.00156 | BIRC5,CCNB1,DPF2,EZH2,HMGB2,HORMAD1,IRF4,JUNB,MEF2A,SMAD2,SMARCE1,TP53 |
| GO.0044454 | nuclear chromosome part | 11 | 0.00213 | CCNB1,DPF2,EZH2,HMGB2,HORMAD1,IRF4,JUNB,MEF2A,SMAD2,SMARCE1,TP53 |
| GO.0000785 | chromatin | 11 | 0.00437 | DPF2,EZH2,HMGB2,IRF4,JUNB,MECP2,MEF2A,NFE2L2,SMAD2,SMARCE1,TP53 |
| GO.0005667 | transcription factor complex | 10 | 0.00156 | CDK2,GTF2A2,GTF2H1,HNF1B,JUNB,MEF2A,NFYA,PBX1,PKNOX1,SMAD2 |
| GO.0000790 | nuclear chromatin | 9 | 0.00289 | DPF2,EZH2,HMGB2,IRF4,JUNB,MEF2A,SMAD2,SMARCE1,TP53 |
