# Supplementary material for: A diagnostic autoantibody signature for primary cutaneous melanoma
Source: Oncotarget. 2018 Jul 17;9(55):30539–51. doi: 10.18632/oncotarget.25669 (PMC6078131; doi:10.18632/oncotarget.25669)
Supplement: Supplementary file 8 [file oncotarget-09-30539-s008.docx]

| **#pathway ID (KEGG pathway)** | **pathway description** | **observed gene count** | **false discovery rate** | **matching proteins in your network (labels)** |
| --- | --- | --- | --- | --- |
| 5200 | Pathways in cancer | 16 | 1.69E-07 | BAD,BIRC5,BIRC7,CBLC,CCND1,CTNNA2,KIT,KLK3,MAPK8,MAX,RAC2,SMAD2,STAT5A,TP53,TRAF2,VEGFB |
| 5161 | Hepatitis B | 10 | 5.49E-06 | BAD,BIRC5,CCND1,CREB5,ELK1,MAPK8,STAT4,STAT5A,TBK1,TP53 |
| 5166 | HTLV-I infection | 10 | 0.000398 | CCND1,CDKN2C,CHEK2,ELK1,MAPK8,SLC25A6,SMAD2,STAT5A,TP53,XBP1 |
| 4010 | MAPK signaling pathway | 9 | 0.00134 | ELK1,HSPA1A,MAPK8,MAX,NLK,RAC2,STMN1,TP53,TRAF2 |
| 5203 | Viral carcinogenesis | 8 | 0.00097 | BAD,CCND1,CREB5,GTF2A2,GTF2H1,STAT5A,TP53,TRAF2 |
| 4014 | Ras signaling pathway | 8 | 0.00205 | BAD,ELK1,KIT,MAPK8,PLD2,RAC2,TBK1,VEGFB |
| 5205 | Proteoglycans in cancer | 8 | 0.00205 | CBLC,CCND1,ELK1,EZR,MSN,PDPK1,SMAD2,TP53 |
| 4151 | PI3K-Akt signaling pathway | 8 | 0.0191 | BAD,CCND1,CREB5,KIT,PDPK1,PPP2CB,TP53,VEGFB |
| 5210 | Colorectal cancer | 7 | 1.09E-05 | BAD,BIRC5,CCND1,MAPK8,RAC2,SMAD2,TP53 |
| 4110 | Cell cycle | 7 | 0.000781 | CCNB1,CCND1,CDC25A,CDKN2C,CHEK2,SMAD2,TP53 |
| 5160 | Hepatitis C | 7 | 0.000874 | BAD,MAPK8,PDPK1,PPP2CB,TBK1,TP53,TRAF2 |
| 5206 | MicroRNAs in cancer | 7 | 0.00134 | CCND1,CDC25A,EZH2,EZR,SERPINB5,STMN1,TP53 |
| 4141 | Protein processing in endoplasmic reticulum | 7 | 0.00205 | HSPA1A,MAPK8,NFE2L2,RAD23B,STUB1,TRAF2,XBP1 |
| 4510 | Focal adhesion | 7 | 0.00561 | BAD,CCND1,ELK1,MAPK8,PDPK1,RAC2,VEGFB |
| 5213 | Endometrial cancer | 6 | 7.87E-05 | BAD,CCND1,CTNNA2,ELK1,PDPK1,TP53 |
| 5212 | Pancreatic cancer | 6 | 0.000207 | BAD,CCND1,MAPK8,RAC2,SMAD2,TP53 |
| 5215 | Prostate cancer | 6 | 0.000874 | BAD,CCND1,CREB5,KLK3,PDPK1,TP53 |
| 4068 | FoxO signaling pathway | 6 | 0.00258 | CCNB1,CCND1,MAPK8,NLK,PDPK1,SMAD2 |
| 5162 | Measles | 6 | 0.00337 | CCND1,HSPA1A,MSN,STAT5A,TBK1,TP53 |
| 5168 | Herpes simplex infection | 6 | 0.0117 | EEF1D,HCFC2,MAPK8,TBK1,TP53,TRAF2 |
| 4115 | p53 signaling pathway | 5 | 0.00191 | CCNB1,CCND1,CHEK2,SERPINB5,TP53 |
| 4520 | Adherens junction | 5 | 0.00205 | CTNNA2,NLK,RAC2,SMAD2,WAS |
| 5220 | Chronic myeloid leukemia | 5 | 0.00205 | BAD,CBLC,CCND1,STAT5A,TP53 |
| 4012 | ErbB signaling pathway | 5 | 0.00337 | BAD,CBLC,ELK1,MAPK8,STAT5A |
| 4210 | Apoptosis | 5 | 0.00337 | BAD,BIRC7,CASP7,TP53,TRAF2 |
| 5222 | Small cell lung cancer | 5 | 0.00337 | BIRC7,CCND1,MAX,TP53,TRAF2 |
| 4668 | TNF signaling pathway | 5 | 0.00838 | CASP7,CREB5,JUNB,MAPK8,TRAF2 |
| 5145 | Toxoplasmosis | 5 | 0.0104 | BAD,BIRC7,HSPA1A,MAPK8,PDPK1 |
| 4722 | Neurotrophin signaling pathway | 5 | 0.0113 | BAD,MAPK8,PDPK1,PRDM4,TP53 |
| 4910 | Insulin signaling pathway | 5 | 0.0191 | BAD,CBLC,ELK1,MAPK8,PDPK1 |
| 4310 | Wnt signaling pathway | 5 | 0.0201 | CCND1,MAPK8,NLK,RAC2,TP53 |
| 4390 | Hippo signaling pathway | 5 | 0.0273 | BIRC5,CCND1,CTNNA2,PPP2CB,SMAD2 |
| 5223 | Non-small cell lung cancer | 4 | 0.00569 | BAD,CCND1,PDPK1,TP53 |
| 5221 | Acute myeloid leukemia | 4 | 0.00587 | BAD,CCND1,KIT,STAT5A |
| 5130 | Pathogenic Escherichia coli infection | 3 | 0.0436 | EZR,TUBB,WAS |
