# Supplementary material for: A diagnostic autoantibody signature for primary cutaneous melanoma
Source: Oncotarget. 2018 Jul 17;9(55):30539–51. doi: 10.18632/oncotarget.25669 (PMC6078131; doi:10.18632/oncotarget.25669)
Supplement: Supplementary file 9 [file oncotarget-09-30539-s009.docx]

| **#node1** | **node2** | **node1_string_internal_id** | **node2_string_internal_id** | **node1_external_id** | **node2_external_id** | **neighborhood_on_chromosome** | **gene_fusion** | **phylogenetic_cooccurrence** | **homology** | **coexpression** | **experimentally_determined_interaction** | **database_annotated** | **automated_textmining** | **combined_score** |
| --- | --- | --- | --- | --- | --- | --- | --- | --- | --- | --- | --- | --- | --- | --- |
| **NFE2L2** | **MAFG** | 1858451 | 1853584 | 9606.ENSP00000380252 | 9606.ENSP00000350369 | 0 | 0 | 0 | 0 | 0 | 0.985 | 0.9 | 0.684 | 0.999 |
| **CHEK2** | **TP53** | 1857546 | 1846083 | 9606.ENSP00000372023 | 9606.ENSP00000269305 | 0 | 0 | 0 | 0 | 0 | 0.996 | 0.9 | 0.973 | 0.999 |
| **CDK2** | **CCNB1** | 1845906 | 1844435 | 9606.ENSP00000266970 | 9606.ENSP00000256442 | 0 | 0 | 0 | 0 | 0.646 | 0.999 | 0.9 | 0.951 | 0.999 |
| **TP53** | **CDK2** | 1846083 | 1845906 | 9606.ENSP00000269305 | 9606.ENSP00000266970 | 0 | 0 | 0 | 0 | 0 | 0.982 | 0.9 | 0.979 | 0.999 |
| **TP53** | **STUB1** | 1846083 | 1842759 | 9606.ENSP00000269305 | 9606.ENSP00000219548 | 0 | 0 | 0 | 0 | 0 | 0.999 | 0 | 0.504 | 0.999 |
| **TBK1** | **TRAF2** | 1851202 | 1843873 | 9606.ENSP00000329967 | 9606.ENSP00000247668 | 0 | 0 | 0 | 0 | 0 | 0.997 | 0.9 | 0.216 | 0.999 |
| **MAPK8** | **JUNB** | 1853988 | 1848518 | 9606.ENSP00000353483 | 9606.ENSP00000303315 | 0 | 0 | 0 | 0 | 0 | 0.892 | 0.8 | 0.972 | 0.999 |
| **CDC25A** | **CDK2** | 1848560 | 1845906 | 9606.ENSP00000303706 | 9606.ENSP00000266970 | 0 | 0 | 0 | 0 | 0.076 | 0.983 | 0.9 | 0.997 | 0.999 |
| **CHEK2** | **CDC25A** | 1857546 | 1848560 | 9606.ENSP00000372023 | 9606.ENSP00000303706 | 0 | 0 | 0 | 0 | 0.137 | 0.985 | 0.9 | 0.984 | 0.999 |
| **MAPK8** | **TP53** | 1853988 | 1846083 | 9606.ENSP00000353483 | 9606.ENSP00000269305 | 0 | 0 | 0 | 0 | 0 | 0.999 | 0.9 | 0.942 | 0.999 |
| **CDK2** | **CCND1** | 1845906 | 1843117 | 9606.ENSP00000266970 | 9606.ENSP00000227507 | 0 | 0 | 0 | 0 | 0 | 0.985 | 0.72 | 0.992 | 0.999 |
| **TP53** | **SMAD2** | 1846083 | 1845079 | 9606.ENSP00000269305 | 9606.ENSP00000262160 | 0 | 0 | 0 | 0 | 0 | 0.987 | 0 | 0.912 | 0.998 |
| **CDC25A** | **CCNB1** | 1848560 | 1844435 | 9606.ENSP00000303706 | 9606.ENSP00000256442 | 0 | 0 | 0 | 0 | 0.228 | 0.899 | 0.9 | 0.839 | 0.998 |
| **TP53** | **CCNB1** | 1846083 | 1844435 | 9606.ENSP00000269305 | 9606.ENSP00000256442 | 0 | 0 | 0 | 0 | 0.07 | 0 | 0.9 | 0.96 | 0.996 |
| **TGIF1** | **SMAD2** | 1850975 | 1845079 | 9606.ENSP00000327959 | 9606.ENSP00000262160 | 0 | 0 | 0 | 0 | 0 | 0.912 | 0.9 | 0.413 | 0.994 |
| **SMAD2** | **ACVR2A** | 1845079 | 1843596 | 9606.ENSP00000262160 | 9606.ENSP00000241416 | 0 | 0 | 0 | 0 | 0.131 | 0 | 0.9 | 0.924 | 0.992 |
| **STAT5A** | **CCND1** | 1852428 | 1843117 | 9606.ENSP00000341208 | 9606.ENSP00000227507 | 0 | 0 | 0 | 0 | 0 | 0 | 0.9 | 0.925 | 0.992 |
| **UBE2V1** | **STUB1** | 1852312 | 1842759 | 9606.ENSP00000340305 | 9606.ENSP00000219548 | 0 | 0 | 0 | 0 | 0 | 0.962 | 0.72 | 0.229 | 0.991 |
| **MAPK8** | **BAD** | 1853988 | 1849142 | 9606.ENSP00000353483 | 9606.ENSP00000309103 | 0 | 0 | 0 | 0 | 0 | 0.897 | 0.9 | 0.126 | 0.99 |
| **SERPINB5** | **TP53** | 1857570 | 1846083 | 9606.ENSP00000372221 | 9606.ENSP00000269305 | 0 | 0 | 0 | 0 | 0 | 0 | 0.9 | 0.912 | 0.99 |
| **TP53** | **GTF2H1** | 1846083 | 1845828 | 9606.ENSP00000269305 | 9606.ENSP00000265963 | 0 | 0 | 0 | 0 | 0 | 0.983 | 0 | 0.33 | 0.988 |
| **PLD2** | **RAC2** | 1845281 | 1843949 | 9606.ENSP00000263088 | 9606.ENSP00000249071 | 0 | 0 | 0 | 0 | 0 | 0.663 | 0 | 0.963 | 0.987 |
| **STAT5A** | **KIT** | 1852428 | 1847112 | 9606.ENSP00000341208 | 9606.ENSP00000288135 | 0 | 0 | 0 | 0 | 0 | 0.595 | 0.9 | 0.694 | 0.986 |
| **NFYA** | **TP53** | 1852963 | 1846083 | 9606.ENSP00000345702 | 9606.ENSP00000269305 | 0 | 0 | 0 | 0 | 0 | 0.809 | 0.9 | 0.349 | 0.986 |
| **CASP7** | **BIRC5** | 1855004 | 1848314 | 9606.ENSP00000358327 | 9606.ENSP00000301633 | 0 | 0 | 0 | 0 | 0 | 0.924 | 0 | 0.799 | 0.984 |
| **RAD23B** | **GTF2H1** | 1853623 | 1845828 | 9606.ENSP00000350708 | 9606.ENSP00000265963 | 0 | 0 | 0 | 0 | 0 | 0.688 | 0.9 | 0.543 | 0.984 |
| **CDK2** | **SMAD2** | 1845906 | 1845079 | 9606.ENSP00000266970 | 9606.ENSP00000262160 | 0 | 0 | 0 | 0 | 0 | 0.567 | 0 | 0.955 | 0.98 |
| **MAPK8** | **TRAF2** | 1853988 | 1843873 | 9606.ENSP00000353483 | 9606.ENSP00000247668 | 0 | 0 | 0 | 0 | 0 | 0.576 | 0.9 | 0.581 | 0.98 |
| **TP53** | **CCND1** | 1846083 | 1843117 | 9606.ENSP00000269305 | 9606.ENSP00000227507 | 0 | 0 | 0 | 0 | 0 | 0 | 0 | 0.979 | 0.979 |
| **STAT5A** | **STAP1** | 1852428 | 1845746 | 9606.ENSP00000341208 | 9606.ENSP00000265404 | 0 | 0 | 0 | 0 | 0 | 0 | 0.9 | 0.802 | 0.979 |
| **MAPK8** | **MSN** | 1853988 | 1853976 | 9606.ENSP00000353483 | 9606.ENSP00000353408 | 0 | 0 | 0 | 0 | 0 | 0.667 | 0 | 0.938 | 0.978 |
| **CDC25A** | **CCND1** | 1848560 | 1843117 | 9606.ENSP00000303706 | 9606.ENSP00000227507 | 0 | 0 | 0 | 0 | 0 | 0 | 0.9 | 0.766 | 0.975 |
| **JUNB** | **SMAD2** | 1848518 | 1845079 | 9606.ENSP00000303315 | 9606.ENSP00000262160 | 0 | 0 | 0 | 0 | 0 | 0.098 | 0.9 | 0.728 | 0.973 |
| **CDKN2C** | **CCND1** | 1845196 | 1843117 | 9606.ENSP00000262662 | 9606.ENSP00000227507 | 0 | 0 | 0 | 0 | 0 | 0 | 0.9 | 0.7 | 0.968 |
| **IFI16** | **TP53** | 1854740 | 1846083 | 9606.ENSP00000357113 | 9606.ENSP00000269305 | 0 | 0 | 0 | 0 | 0 | 0.812 | 0 | 0.833 | 0.967 |
| **BIRC5** | **TP53** | 1848314 | 1846083 | 9606.ENSP00000301633 | 9606.ENSP00000269305 | 0 | 0 | 0 | 0 | 0 | 0 | 0 | 0.963 | 0.964 |
| **EZH2** | **CDK2** | 1850211 | 1845906 | 9606.ENSP00000320147 | 9606.ENSP00000266970 | 0 | 0 | 0 | 0 | 0 | 0.576 | 0 | 0.917 | 0.963 |
| **KIT** | **STAP1** | 1847112 | 1845746 | 9606.ENSP00000288135 | 9606.ENSP00000265404 | 0 | 0 | 0 | 0 | 0 | 0.625 | 0.9 | 0.073 | 0.962 |
| **BIRC5** | **CCND1** | 1848314 | 1843117 | 9606.ENSP00000301633 | 9606.ENSP00000227507 | 0 | 0 | 0 | 0 | 0 | 0 | 0 | 0.96 | 0.96 |
| **GTF2A2** | **GTF2H1** | 1858360 | 1845828 | 9606.ENSP00000379372 | 9606.ENSP00000265963 | 0 | 0 | 0 | 0 | 0 | 0 | 0.9 | 0.609 | 0.959 |
| **NFYA** | **CDK2** | 1852963 | 1845906 | 9606.ENSP00000345702 | 9606.ENSP00000266970 | 0 | 0 | 0 | 0 | 0 | 0.57 | 0 | 0.907 | 0.958 |
| **CASP7** | **BIRC7** | 1855004 | 1842643 | 9606.ENSP00000358327 | 9606.ENSP00000217169 | 0 | 0 | 0 | 0 | 0 | 0.614 | 0.8 | 0.476 | 0.956 |
| **IMPA1** | **INPP1** | 1860725 | 1850736 | 9606.ENSP00000408526 | 9606.ENSP00000325423 | 0 | 0 | 0 | 0 | 0 | 0 | 0.9 | 0.523 | 0.95 |
| **MEF2A** | **TBX5** | 1853046 | 1849239 | 9606.ENSP00000346389 | 9606.ENSP00000309913 | 0 | 0 | 0 | 0 | 0 | 0 | 0 | 0.945 | 0.946 |
| **BIRC5** | **CDK2** | 1848314 | 1845906 | 9606.ENSP00000301633 | 9606.ENSP00000266970 | 0 | 0 | 0 | 0 | 0.289 | 0.576 | 0 | 0.827 | 0.943 |
| **CDC25A** | **TP53** | 1848560 | 1846083 | 9606.ENSP00000303706 | 9606.ENSP00000269305 | 0 | 0 | 0 | 0 | 0 | 0 | 0 | 0.941 | 0.941 |
| **CCNB1** | **CCND1** | 1844435 | 1843117 | 9606.ENSP00000256442 | 9606.ENSP00000227507 | 0 | 0 | 0 | 0.65 | 0.105 | 0.087 | 0.9 | 0.9 | 0.939 |
| **MEF2A** | **SMAD2** | 1853046 | 1845079 | 9606.ENSP00000346389 | 9606.ENSP00000262160 | 0 | 0 | 0 | 0 | 0 | 0.576 | 0 | 0.855 | 0.936 |
| **MSN** | **EZR** | 1853976 | 1852156 | 9606.ENSP00000353408 | 9606.ENSP00000338934 | 0 | 0 | 0 | 0.98 | 0 | 0.934 | 0 | 0.943 | 0.936 |
| **FEN1** | **GTF2H1** | 1848737 | 1845828 | 9606.ENSP00000305480 | 9606.ENSP00000265963 | 0 | 0 | 0 | 0 | 0.111 | 0.305 | 0 | 0.9 | 0.933 |
| **MAX** | **TP53** | 1853721 | 1846083 | 9606.ENSP00000351490 | 9606.ENSP00000269305 | 0 | 0 | 0 | 0 | 0 | 0 | 0.9 | 0.31 | 0.928 |
| **STAT5A** | **CCNB1** | 1852428 | 1844435 | 9606.ENSP00000341208 | 9606.ENSP00000256442 | 0 | 0 | 0 | 0 | 0 | 0 | 0.9 | 0.291 | 0.926 |
| **MAPK8** | **CCND1** | 1853988 | 1843117 | 9606.ENSP00000353483 | 9606.ENSP00000227507 | 0 | 0 | 0 | 0 | 0 | 0 | 0 | 0.926 | 0.926 |
| **EZH2** | **TP53** | 1850211 | 1846083 | 9606.ENSP00000320147 | 9606.ENSP00000269305 | 0 | 0 | 0 | 0 | 0 | 0 | 0 | 0.921 | 0.921 |
| **TP53** | **PATZ1** | 1846083 | 1845864 | 9606.ENSP00000269305 | 9606.ENSP00000266269 | 0 | 0 | 0 | 0 | 0 | 0.576 | 0 | 0.822 | 0.921 |
| **MAX** | **CDC25A** | 1853721 | 1848560 | 9606.ENSP00000351490 | 9606.ENSP00000303706 | 0 | 0 | 0 | 0 | 0 | 0 | 0.9 | 0.25 | 0.921 |
| **MAPK8** | **KIT** | 1853988 | 1847112 | 9606.ENSP00000353483 | 9606.ENSP00000288135 | 0 | 0 | 0 | 0.62 | 0 | 0.107 | 0.9 | 0.421 | 0.92 |
| **KLK3** | **CCND1** | 1849687 | 1843117 | 9606.ENSP00000314151 | 9606.ENSP00000227507 | 0 | 0 | 0 | 0 | 0 | 0 | 0.9 | 0.196 | 0.916 |
| **MAPK8** | **RAC2** | 1853988 | 1843949 | 9606.ENSP00000353483 | 9606.ENSP00000249071 | 0 | 0 | 0 | 0 | 0 | 0.313 | 0.8 | 0.438 | 0.916 |
| **MAPK8** | **ELK1** | 1853988 | 1843855 | 9606.ENSP00000353483 | 9606.ENSP00000247161 | 0 | 0 | 0 | 0 | 0 | 0.576 | 0.8 | 0.089 | 0.916 |
| **MAX** | **CCNB1** | 1853721 | 1844435 | 9606.ENSP00000351490 | 9606.ENSP00000256442 | 0 | 0 | 0 | 0 | 0 | 0 | 0.9 | 0.191 | 0.915 |
| **STMN1** | **CCNB1** | 1860849 | 1844435 | 9606.ENSP00000410452 | 9606.ENSP00000256442 | 0 | 0 | 0 | 0 | 0 | 0 | 0.9 | 0.194 | 0.915 |
| **STAT5A** | **TP53** | 1852428 | 1846083 | 9606.ENSP00000341208 | 9606.ENSP00000269305 | 0 | 0 | 0 | 0 | 0 | 0 | 0 | 0.913 | 0.913 |
| **AK2** | **NME5** | 1853107 | 1845708 | 9606.ENSP00000346921 | 9606.ENSP00000265191 | 0 | 0 | 0 | 0 | 0 | 0 | 0.9 | 0.16 | 0.912 |
| **PBX1** | **PKNOX1** | 1860561 | 1847291 | 9606.ENSP00000405890 | 9606.ENSP00000291547 | 0 | 0 | 0 | 0.626 | 0 | 0.86 | 0 | 0.966 | 0.911 |
| **GTF2H1** | **SUPT4H1** | 1845828 | 1843026 | 9606.ENSP00000265963 | 9606.ENSP00000225504 | 0 | 0 | 0 | 0 | 0 | 0 | 0.9 | 0.138 | 0.91 |
| **MAX** | **BIRC5** | 1853721 | 1848314 | 9606.ENSP00000351490 | 9606.ENSP00000301633 | 0 | 0 | 0 | 0 | 0.083 | 0 | 0.9 | 0.101 | 0.91 |
| **MAX** | **CDK2** | 1853721 | 1845906 | 9606.ENSP00000351490 | 9606.ENSP00000266970 | 0 | 0 | 0 | 0 | 0 | 0 | 0.9 | 0.143 | 0.91 |
| **CHEK2** | **CCNB1** | 1857546 | 1844435 | 9606.ENSP00000372023 | 9606.ENSP00000256442 | 0 | 0 | 0 | 0 | 0.479 | 0.193 | 0 | 0.803 | 0.91 |
| **FAF1** | **CASP7** | 1855550 | 1855004 | 9606.ENSP00000360843 | 9606.ENSP00000358327 | 0 | 0 | 0 | 0 | 0 | 0 | 0.9 | 0.125 | 0.908 |
| **MAX** | **SMAD2** | 1853721 | 1845079 | 9606.ENSP00000351490 | 9606.ENSP00000262160 | 0 | 0 | 0 | 0 | 0 | 0.098 | 0.9 | 0.071 | 0.908 |
| **CTNNA2** | **MEF2A** | 1861346 | 1853046 | 9606.ENSP00000418191 | 9606.ENSP00000346389 | 0 | 0 | 0 | 0 | 0 | 0 | 0.9 | 0.102 | 0.906 |
| **MAX** | **ZFP36L1** | 1853721 | 1851991 | 9606.ENSP00000351490 | 9606.ENSP00000337386 | 0 | 0 | 0 | 0 | 0 | 0 | 0.9 | 0.062 | 0.902 |
| **PDPK1** | **UBE2V1** | 1852785 | 1852312 | 9606.ENSP00000344220 | 9606.ENSP00000340305 | 0 | 0 | 0 | 0 | 0 | 0 | 0.9 | 0.053 | 0.901 |
| **TGIF1** | **KLK3** | 1850975 | 1849687 | 9606.ENSP00000327959 | 9606.ENSP00000314151 | 0 | 0 | 0 | 0 | 0 | 0 | 0.9 | 0 | 0.9 |
| **SMARCE1** | **KLK3** | 1850602 | 1849687 | 9606.ENSP00000323967 | 9606.ENSP00000314151 | 0 | 0 | 0 | 0 | 0 | 0 | 0.9 | 0 | 0.9 |
| **MAX** | **NDRG2** | 1853721 | 1847981 | 9606.ENSP00000351490 | 9606.ENSP00000298687 | 0 | 0 | 0 | 0 | 0 | 0 | 0.9 | 0 | 0.9 |
| **MAX** | **NFYA** | 1853721 | 1852963 | 9606.ENSP00000351490 | 9606.ENSP00000345702 | 0 | 0 | 0 | 0 | 0 | 0 | 0.9 | 0 | 0.9 |
| **CDC25A** | **PSME2** | 1848560 | 1842623 | 9606.ENSP00000303706 | 9606.ENSP00000216802 | 0 | 0 | 0 | 0 | 0 | 0 | 0.9 | 0 | 0.9 |
| **PDPK1** | **BAD** | 1852785 | 1849142 | 9606.ENSP00000344220 | 9606.ENSP00000309103 | 0 | 0 | 0 | 0 | 0 | 0 | 0.9 | 0 | 0.9 |
| **KLK3** | **PATZ1** | 1849687 | 1845864 | 9606.ENSP00000314151 | 9606.ENSP00000266269 | 0 | 0 | 0 | 0 | 0 | 0 | 0.9 | 0 | 0.9 |
| **CCND1** | **PSME2** | 1843117 | 1842623 | 9606.ENSP00000227507 | 9606.ENSP00000216802 | 0 | 0 | 0 | 0 | 0 | 0 | 0.9 | 0.041 | 0.9 |
| **IRF4** | **SMARCE1** | 1857309 | 1850602 | 9606.ENSP00000370343 | 9606.ENSP00000323967 | 0 | 0 | 0 | 0 | 0 | 0 | 0.9 | 0.043 | 0.9 |
| **CDK2** | **PSME2** | 1845906 | 1842623 | 9606.ENSP00000266970 | 9606.ENSP00000216802 | 0 | 0 | 0 | 0 | 0 | 0 | 0.9 | 0 | 0.9 |
| **EZH2** | **CCND1** | 1850211 | 1843117 | 9606.ENSP00000320147 | 9606.ENSP00000227507 | 0 | 0 | 0 | 0 | 0 | 0 | 0 | 0.895 | 0.895 |
| **MAPK8** | **EZR** | 1853988 | 1852156 | 9606.ENSP00000353483 | 9606.ENSP00000338934 | 0 | 0 | 0 | 0 | 0 | 0.667 | 0 | 0.697 | 0.895 |
| **NFE2L2** | **TP53** | 1858451 | 1846083 | 9606.ENSP00000380252 | 9606.ENSP00000269305 | 0 | 0 | 0 | 0 | 0 | 0 | 0 | 0.889 | 0.889 |
| **STK38L** | **NDRG2** | 1857786 | 1847981 | 9606.ENSP00000373684 | 9606.ENSP00000298687 | 0 | 0 | 0 | 0 | 0 | 0 | 0 | 0.887 | 0.887 |
| **RAD23B** | **TP53** | 1853623 | 1846083 | 9606.ENSP00000350708 | 9606.ENSP00000269305 | 0 | 0 | 0 | 0 | 0 | 0 | 0 | 0.878 | 0.878 |
| **STMN1** | **TP53** | 1860849 | 1846083 | 9606.ENSP00000410452 | 9606.ENSP00000269305 | 0 | 0 | 0 | 0 | 0 | 0 | 0 | 0.874 | 0.874 |
| **NR1I2** | **TP53** | 1851892 | 1846083 | 9606.ENSP00000336528 | 9606.ENSP00000269305 | 0 | 0 | 0 | 0 | 0 | 0 | 0 | 0.862 | 0.862 |
| **PKNOX1** | **SMAD2** | 1847291 | 1845079 | 9606.ENSP00000291547 | 9606.ENSP00000262160 | 0 | 0 | 0 | 0 | 0 | 0.104 | 0 | 0.85 | 0.86 |
| **CASP7** | **TP53** | 1855004 | 1846083 | 9606.ENSP00000358327 | 9606.ENSP00000269305 | 0 | 0 | 0 | 0 | 0 | 0 | 0 | 0.86 | 0.86 |
| **CDK16** | **CCND1** | 1846467 | 1843117 | 9606.ENSP00000276052 | 9606.ENSP00000227507 | 0 | 0 | 0 | 0 | 0 | 0.36 | 0 | 0.778 | 0.852 |
| **CDK2** | **CDKN2C** | 1845906 | 1845196 | 9606.ENSP00000266970 | 9606.ENSP00000262662 | 0 | 0 | 0 | 0 | 0.094 | 0.149 | 0 | 0.819 | 0.848 |
| **VEGFB** | **KIT** | 1849383 | 1847112 | 9606.ENSP00000311127 | 9606.ENSP00000288135 | 0 | 0 | 0 | 0 | 0 | 0 | 0.8 | 0.242 | 0.841 |
| **CDC25A** | **CDK16** | 1848560 | 1846467 | 9606.ENSP00000303706 | 9606.ENSP00000276052 | 0 | 0 | 0 | 0 | 0 | 0.301 | 0 | 0.778 | 0.839 |
| **PAPSS2** | **NR1I2** | 1860582 | 1851892 | 9606.ENSP00000406157 | 9606.ENSP00000336528 | 0 | 0 | 0 | 0 | 0 | 0 | 0 | 0.834 | 0.834 |
| **HEXIM1** | **CCND1** | 1851074 | 1843117 | 9606.ENSP00000328773 | 9606.ENSP00000227507 | 0 | 0 | 0 | 0 | 0 | 0 | 0 | 0.832 | 0.832 |
| **NR1I2** | **CDK2** | 1851892 | 1845906 | 9606.ENSP00000336528 | 9606.ENSP00000266970 | 0 | 0 | 0 | 0 | 0 | 0 | 0 | 0.832 | 0.832 |
| **BIRC5** | **BIRC7** | 1848314 | 1842643 | 9606.ENSP00000301633 | 9606.ENSP00000217169 | 0 | 0 | 0 | 0 | 0 | 0 | 0 | 0.83 | 0.83 |
| **CEP55** | **TP53** | 1855493 | 1846083 | 9606.ENSP00000360540 | 9606.ENSP00000269305 | 0 | 0 | 0 | 0 | 0 | 0.063 | 0 | 0.825 | 0.829 |
| **NLK** | **MEF2A** | 1858963 | 1853046 | 9606.ENSP00000384625 | 9606.ENSP00000346389 | 0 | 0 | 0 | 0 | 0 | 0 | 0 | 0.826 | 0.826 |
| **MAPK8** | **SMAD2** | 1853988 | 1845079 | 9606.ENSP00000353483 | 9606.ENSP00000262160 | 0 | 0 | 0 | 0 | 0 | 0.576 | 0 | 0.601 | 0.823 |
| **HSPA1A** | **BAG3** | 1856351 | 1854947 | 9606.ENSP00000364802 | 9606.ENSP00000358081 | 0 | 0 | 0 | 0 | 0.115 | 0.685 | 0 | 0.414 | 0.822 |
| **TBX6** | **SMAD2** | 1846629 | 1845079 | 9606.ENSP00000279386 | 9606.ENSP00000262160 | 0 | 0 | 0 | 0 | 0 | 0 | 0 | 0.821 | 0.822 |
| **JUNB** | **CDK2** | 1848518 | 1845906 | 9606.ENSP00000303315 | 9606.ENSP00000266970 | 0 | 0 | 0 | 0 | 0 | 0.337 | 0 | 0.737 | 0.819 |
| **PKNOX1** | **TP53** | 1847291 | 1846083 | 9606.ENSP00000291547 | 9606.ENSP00000269305 | 0 | 0 | 0 | 0 | 0 | 0 | 0 | 0.819 | 0.819 |
| **BIRC5** | **CCNB1** | 1848314 | 1844435 | 9606.ENSP00000301633 | 9606.ENSP00000256442 | 0 | 0 | 0 | 0 | 0.289 | 0 | 0 | 0.755 | 0.818 |
| **KIT** | **CBLC** | 1847112 | 1846144 | 9606.ENSP00000288135 | 9606.ENSP00000270279 | 0 | 0 | 0 | 0 | 0 | 0 | 0.8 | 0.129 | 0.818 |
| **RING1** | **EZH2** | 1856158 | 1850211 | 9606.ENSP00000363787 | 9606.ENSP00000320147 | 0 | 0 | 0 | 0 | 0 | 0 | 0 | 0.807 | 0.807 |
| **HSPA1A** | **TP53** | 1856351 | 1846083 | 9606.ENSP00000364802 | 9606.ENSP00000269305 | 0 | 0 | 0 | 0 | 0 | 0.576 | 0 | 0.539 | 0.796 |
| **CDK16** | **CCNB1** | 1846467 | 1844435 | 9606.ENSP00000276052 | 9606.ENSP00000256442 | 0 | 0 | 0 | 0 | 0.11 | 0.339 | 0 | 0.681 | 0.796 |
| **FEN1** | **CDK2** | 1848737 | 1845906 | 9606.ENSP00000305480 | 9606.ENSP00000266970 | 0 | 0 | 0 | 0 | 0.138 | 0.661 | 0 | 0.347 | 0.792 |
| **CDK18** | **CDC25A** | 1861603 | 1848560 | 9606.ENSP00000423665 | 9606.ENSP00000303706 | 0 | 0 | 0 | 0 | 0 | 0.301 | 0 | 0.712 | 0.79 |
| **JUNB** | **CDK16** | 1848518 | 1846467 | 9606.ENSP00000303315 | 9606.ENSP00000276052 | 0 | 0 | 0 | 0 | 0 | 0.337 | 0 | 0.694 | 0.789 |
| **HSPA1A** | **STUB1** | 1856351 | 1842759 | 9606.ENSP00000364802 | 9606.ENSP00000219548 | 0 | 0 | 0 | 0 | 0 | 0.709 | 0 | 0.296 | 0.787 |
| **NDRG2** | **PPP2CB** | 1847981 | 1842815 | 9606.ENSP00000298687 | 9606.ENSP00000221138 | 0 | 0 | 0 | 0 | 0 | 0 | 0 | 0.78 | 0.78 |
| **CDK18** | **JUNB** | 1861603 | 1848518 | 9606.ENSP00000423665 | 9606.ENSP00000303315 | 0 | 0 | 0 | 0 | 0 | 0.337 | 0 | 0.672 | 0.774 |
| **PBX1** | **DLX1** | 1860561 | 1854126 | 9606.ENSP00000405890 | 9606.ENSP00000354478 | 0 | 0 | 0 | 0 | 0 | 0 | 0 | 0.772 | 0.773 |
| **CDK18** | **CCND1** | 1861603 | 1843117 | 9606.ENSP00000423665 | 9606.ENSP00000227507 | 0 | 0 | 0 | 0 | 0 | 0.36 | 0 | 0.619 | 0.746 |
| **PBX1** | **DLX3** | 1860561 | 1859461 | 9606.ENSP00000405890 | 9606.ENSP00000389870 | 0 | 0 | 0 | 0 | 0 | 0 | 0 | 0.74 | 0.741 |
| **NFE2L2** | **MAPK8** | 1858451 | 1853988 | 9606.ENSP00000380252 | 9606.ENSP00000353483 | 0 | 0 | 0 | 0 | 0 | 0.57 | 0 | 0.418 | 0.739 |
| **MAPK8** | **CREB5** | 1853988 | 1853582 | 9606.ENSP00000353483 | 9606.ENSP00000350359 | 0 | 0 | 0 | 0 | 0 | 0.263 | 0 | 0.654 | 0.735 |
| **CDK18** | **CCNB1** | 1861603 | 1844435 | 9606.ENSP00000423665 | 9606.ENSP00000256442 | 0 | 0 | 0 | 0 | 0.11 | 0.339 | 0 | 0.578 | 0.73 |
| **RAD23B** | **STUB1** | 1853623 | 1842759 | 9606.ENSP00000350708 | 9606.ENSP00000219548 | 0 | 0 | 0 | 0 | 0.098 | 0.684 | 0 | 0.127 | 0.729 |
| **PKNOX1** | **HOXB6** | 1847291 | 1843039 | 9606.ENSP00000291547 | 9606.ENSP00000225648 | 0 | 0 | 0 | 0 | 0 | 0.602 | 0 | 0.347 | 0.729 |
| **BAG3** | **STUB1** | 1854947 | 1842759 | 9606.ENSP00000358081 | 9606.ENSP00000219548 | 0 | 0 | 0 | 0 | 0 | 0.526 | 0 | 0.445 | 0.726 |
| **CCNB1** | **PPP2CB** | 1844435 | 1842815 | 9606.ENSP00000256442 | 9606.ENSP00000221138 | 0 | 0 | 0 | 0 | 0 | 0.281 | 0 | 0.633 | 0.725 |
| **DR1** | **GTF2H1** | 1855206 | 1845828 | 9606.ENSP00000359290 | 9606.ENSP00000265963 | 0 | 0 | 0 | 0 | 0 | 0.665 | 0 | 0.211 | 0.724 |
| **MAPK8** | **CDC25A** | 1853988 | 1848560 | 9606.ENSP00000353483 | 9606.ENSP00000303706 | 0 | 0 | 0 | 0 | 0 | 0 | 0 | 0.722 | 0.722 |
| **KIT** | **TP53** | 1847112 | 1846083 | 9606.ENSP00000288135 | 9606.ENSP00000269305 | 0 | 0 | 0 | 0 | 0 | 0 | 0 | 0.72 | 0.72 |
| **TTF2** | **SUPT4H1** | 1855031 | 1843026 | 9606.ENSP00000358478 | 9606.ENSP00000225504 | 0 | 0 | 0 | 0 | 0 | 0 | 0.72 | 0 | 0.72 |
| **HMGB2** | **TP53** | 1847742 | 1846083 | 9606.ENSP00000296503 | 9606.ENSP00000269305 | 0 | 0 | 0 | 0 | 0 | 0.576 | 0 | 0.36 | 0.717 |
| **DPF2** | **SMARCE1** | 1862050 | 1850602 | 9606.ENSP00000436901 | 9606.ENSP00000323967 | 0 | 0 | 0 | 0 | 0 | 0.588 | 0 | 0.31 | 0.704 |
| **MAPK8** | **HSFY1** | 1853988 | 1848556 | 9606.ENSP00000353483 | 9606.ENSP00000303599 | 0 | 0 | 0 | 0 | 0 | 0 | 0 | 0.693 | 0.693 |
| **DLX1** | **SMAD2** | 1854126 | 1845079 | 9606.ENSP00000354478 | 9606.ENSP00000262160 | 0 | 0 | 0 | 0 | 0 | 0 | 0 | 0.691 | 0.691 |
| **UBE2V1** | **TRAF2** | 1852312 | 1843873 | 9606.ENSP00000340305 | 9606.ENSP00000247668 | 0 | 0 | 0 | 0 | 0 | 0.575 | 0 | 0.301 | 0.69 |
| **PBX1** | **HOXB6** | 1860561 | 1843039 | 9606.ENSP00000405890 | 9606.ENSP00000225648 | 0 | 0 | 0 | 0 | 0 | 0.301 | 0 | 0.571 | 0.687 |
| **MEF2A** | **CDK16** | 1853046 | 1846467 | 9606.ENSP00000346389 | 9606.ENSP00000276052 | 0 | 0 | 0 | 0 | 0 | 0.109 | 0 | 0.653 | 0.678 |
| **EEF1D** | **RPL32** | 1859610 | 1858438 | 9606.ENSP00000391944 | 9606.ENSP00000380156 | 0 | 0 | 0 | 0 | 0.641 | 0 | 0 | 0.124 | 0.672 |
| **CREB5** | **HSFY1** | 1853582 | 1848556 | 9606.ENSP00000350359 | 9606.ENSP00000303599 | 0 | 0 | 0 | 0 | 0 | 0 | 0 | 0.658 | 0.658 |
| **MAPK8** | **PDPK1** | 1853988 | 1852785 | 9606.ENSP00000353483 | 9606.ENSP00000344220 | 0 | 0 | 0.282 | 0.649 | 0 | 0.566 | 0 | 0.381 | 0.653 |
| **HEXIM1** | **TP53** | 1851074 | 1846083 | 9606.ENSP00000328773 | 9606.ENSP00000269305 | 0 | 0 | 0 | 0 | 0 | 0.564 | 0 | 0.234 | 0.651 |
| **TUBB** | **EZR** | 1852162 | 1852156 | 9606.ENSP00000339001 | 9606.ENSP00000338934 | 0 | 0 | 0 | 0 | 0 | 0 | 0 | 0.648 | 0.648 |
| **CCDC33** | **HORMAD1** | 1858658 | 1854315 | 9606.ENSP00000381795 | 9606.ENSP00000355167 | 0 | 0 | 0 | 0 | 0 | 0 | 0 | 0.645 | 0.645 |
| **CDK18** | **MEF2A** | 1861603 | 1853046 | 9606.ENSP00000423665 | 9606.ENSP00000346389 | 0 | 0 | 0 | 0 | 0 | 0.109 | 0 | 0.616 | 0.643 |
| **MUTYH** | **FEN1** | 1855617 | 1848737 | 9606.ENSP00000361170 | 9606.ENSP00000305480 | 0.073 | 0 | 0 | 0 | 0.067 | 0 | 0 | 0.621 | 0.643 |
| **TRAF2** | **STUB1** | 1843873 | 1842759 | 9606.ENSP00000247668 | 9606.ENSP00000219548 | 0 | 0 | 0 | 0 | 0 | 0.626 | 0 | 0.081 | 0.642 |
| **PRKCH** | **CDK2** | 1851111 | 1845906 | 9606.ENSP00000329127 | 9606.ENSP00000266970 | 0 | 0 | 0 | 0.746 | 0 | 0.623 | 0 | 0.211 | 0.64 |
| **HSPA1A** | **XBP1** | 1856351 | 1842534 | 9606.ENSP00000364802 | 9606.ENSP00000216037 | 0 | 0 | 0 | 0 | 0.072 | 0 | 0 | 0.627 | 0.64 |
| **SMAD2** | **STUB1** | 1845079 | 1842759 | 9606.ENSP00000262160 | 9606.ENSP00000219548 | 0 | 0 | 0 | 0 | 0 | 0.567 | 0 | 0.204 | 0.64 |
| **TP53** | **CDKN2C** | 1846083 | 1845196 | 9606.ENSP00000269305 | 9606.ENSP00000262662 | 0 | 0 | 0 | 0 | 0 | 0 | 0 | 0.633 | 0.633 |
| **HMGB2** | **PKNOX1** | 1847742 | 1847291 | 9606.ENSP00000296503 | 9606.ENSP00000291547 | 0 | 0 | 0 | 0 | 0 | 0.621 | 0 | 0.064 | 0.63 |
| **CDK16** | **RAC2** | 1846467 | 1843949 | 9606.ENSP00000276052 | 9606.ENSP00000249071 | 0 | 0 | 0 | 0 | 0 | 0.105 | 0 | 0.602 | 0.629 |
| **MAPK8** | **CDKN2C** | 1853988 | 1845196 | 9606.ENSP00000353483 | 9606.ENSP00000262662 | 0 | 0 | 0 | 0 | 0 | 0.567 | 0 | 0.171 | 0.625 |
| **MSN** | **TUBB** | 1853976 | 1852162 | 9606.ENSP00000353408 | 9606.ENSP00000339001 | 0 | 0 | 0 | 0 | 0 | 0 | 0 | 0.612 | 0.612 |
| **HSFY1** | **TBX6** | 1848556 | 1846629 | 9606.ENSP00000303599 | 9606.ENSP00000279386 | 0 | 0 | 0 | 0 | 0 | 0.612 | 0 | 0 | 0.612 |
| **HSPA1A** | **SMAD2** | 1856351 | 1845079 | 9606.ENSP00000364802 | 9606.ENSP00000262160 | 0 | 0 | 0 | 0 | 0 | 0 | 0 | 0.607 | 0.607 |
| **CHEK2** | **CCND1** | 1857546 | 1843117 | 9606.ENSP00000372023 | 9606.ENSP00000227507 | 0 | 0 | 0 | 0 | 0.1 | 0 | 0 | 0.582 | 0.607 |
| **MEF2A** | **CDK2** | 1853046 | 1845906 | 9606.ENSP00000346389 | 9606.ENSP00000266970 | 0 | 0 | 0 | 0 | 0 | 0.195 | 0 | 0.526 | 0.603 |
| **NLK** | **TP53** | 1858963 | 1846083 | 9606.ENSP00000384625 | 9606.ENSP00000269305 | 0 | 0 | 0 | 0 | 0 | 0.564 | 0 | 0.126 | 0.602 |
| **CDK16** | **PPP2CB** | 1846467 | 1842815 | 9606.ENSP00000276052 | 9606.ENSP00000221138 | 0 | 0 | 0 | 0 | 0 | 0.538 | 0 | 0.171 | 0.601 |
| **CDK18** | **PPP2CB** | 1861603 | 1842815 | 9606.ENSP00000423665 | 9606.ENSP00000221138 | 0 | 0 | 0 | 0 | 0 | 0.559 | 0 | 0.126 | 0.598 |
| **PRKCH** | **RAC2** | 1851111 | 1843949 | 9606.ENSP00000329127 | 9606.ENSP00000249071 | 0 | 0 | 0 | 0 | 0 | 0.302 | 0 | 0.444 | 0.595 |
| **CASP7** | **MEF2A** | 1855004 | 1853046 | 9606.ENSP00000358327 | 9606.ENSP00000346389 | 0 | 0 | 0 | 0 | 0 | 0.576 | 0 | 0.078 | 0.592 |
| **TUBB** | **CCNB1** | 1852162 | 1844435 | 9606.ENSP00000339001 | 9606.ENSP00000256442 | 0 | 0 | 0 | 0 | 0 | 0.088 | 0 | 0.569 | 0.59 |
| **NLK** | **STAT5A** | 1858963 | 1852428 | 9606.ENSP00000384625 | 9606.ENSP00000341208 | 0 | 0 | 0 | 0 | 0 | 0.567 | 0 | 0.081 | 0.585 |
| **CASP7** | **MAX** | 1855004 | 1853721 | 9606.ENSP00000358327 | 9606.ENSP00000351490 | 0 | 0 | 0 | 0 | 0 | 0.576 | 0 | 0.053 | 0.581 |
| **CDK16** | **TP53** | 1846467 | 1846083 | 9606.ENSP00000276052 | 9606.ENSP00000269305 | 0 | 0 | 0 | 0 | 0 | 0 | 0 | 0.581 | 0.581 |
| **CDKN2C** | **CCNB1** | 1845196 | 1844435 | 9606.ENSP00000262662 | 9606.ENSP00000256442 | 0 | 0 | 0 | 0 | 0.071 | 0.129 | 0 | 0.515 | 0.574 |
| **KIT** | **CCND1** | 1847112 | 1843117 | 9606.ENSP00000288135 | 9606.ENSP00000227507 | 0 | 0 | 0 | 0 | 0 | 0 | 0 | 0.573 | 0.573 |
| **RAD23B** | **FEN1** | 1853623 | 1848737 | 9606.ENSP00000350708 | 9606.ENSP00000305480 | 0 | 0 | 0 | 0 | 0.112 | 0.092 | 0 | 0.511 | 0.571 |
| **IRF4** | **XBP1** | 1857309 | 1842534 | 9606.ENSP00000370343 | 9606.ENSP00000216037 | 0 | 0 | 0 | 0 | 0 | 0 | 0 | 0.57 | 0.57 |
| **NFE2L2** | **PRKCH** | 1858451 | 1851111 | 9606.ENSP00000380252 | 9606.ENSP00000329127 | 0 | 0 | 0 | 0 | 0 | 0.57 | 0 | 0 | 0.57 |
| **CDK18** | **RAC2** | 1861603 | 1843949 | 9606.ENSP00000423665 | 9606.ENSP00000249071 | 0 | 0 | 0 | 0 | 0 | 0.105 | 0 | 0.537 | 0.568 |
| **MAPK8** | **XBP1** | 1853988 | 1842534 | 9606.ENSP00000353483 | 9606.ENSP00000216037 | 0 | 0 | 0 | 0 | 0 | 0 | 0 | 0.568 | 0.568 |
| **MAPK8** | **PPP2CB** | 1853988 | 1842815 | 9606.ENSP00000353483 | 9606.ENSP00000221138 | 0 | 0 | 0 | 0 | 0 | 0.567 | 0 | 0 | 0.567 |
| **MLANA** | **KIT** | 1857386 | 1847112 | 9606.ENSP00000370880 | 9606.ENSP00000288135 | 0 | 0 | 0 | 0 | 0 | 0 | 0 | 0.566 | 0.566 |
| **STK38L** | **BAG3** | 1857786 | 1854947 | 9606.ENSP00000373684 | 9606.ENSP00000358081 | 0 | 0 | 0 | 0 | 0.092 | 0.3 | 0 | 0.371 | 0.565 |
| **CASP7** | **MAPK8** | 1855004 | 1853988 | 9606.ENSP00000358327 | 9606.ENSP00000353483 | 0 | 0 | 0 | 0 | 0 | 0 | 0 | 0.562 | 0.562 |
| **HSPA1A** | **HSFY1** | 1856351 | 1848556 | 9606.ENSP00000364802 | 9606.ENSP00000303599 | 0 | 0 | 0 | 0 | 0 | 0.177 | 0 | 0.486 | 0.558 |
| **DR1** | **TTF2** | 1855206 | 1855031 | 9606.ENSP00000359290 | 9606.ENSP00000358478 | 0 | 0 | 0 | 0 | 0 | 0.556 | 0 | 0 | 0.556 |
| **CREB5** | **JUNB** | 1853582 | 1848518 | 9606.ENSP00000350359 | 9606.ENSP00000303315 | 0 | 0 | 0 | 0.63 | 0 | 0.362 | 0 | 0.741 | 0.534 |
| **TUBB** | **CDK2** | 1852162 | 1845906 | 9606.ENSP00000339001 | 9606.ENSP00000266970 | 0 | 0 | 0 | 0 | 0 | 0.092 | 0 | 0.505 | 0.532 |
| **CDK2** | **RAC2** | 1845906 | 1843949 | 9606.ENSP00000266970 | 9606.ENSP00000249071 | 0 | 0 | 0 | 0 | 0 | 0.105 | 0 | 0.497 | 0.53 |
| **MEF2A** | **PRKCH** | 1853046 | 1851111 | 9606.ENSP00000346389 | 9606.ENSP00000329127 | 0 | 0 | 0 | 0 | 0 | 0.107 | 0 | 0.492 | 0.527 |
| **STAT5A** | **CDK2** | 1852428 | 1845906 | 9606.ENSP00000341208 | 9606.ENSP00000266970 | 0 | 0 | 0 | 0 | 0 | 0.164 | 0 | 0.457 | 0.526 |
| **SMAD2** | **CCND1** | 1845079 | 1843117 | 9606.ENSP00000262160 | 9606.ENSP00000227507 | 0 | 0 | 0 | 0 | 0 | 0 | 0 | 0.523 | 0.523 |
| **CDK18** | **TUBB** | 1861603 | 1852162 | 9606.ENSP00000423665 | 9606.ENSP00000339001 | 0 | 0 | 0 | 0 | 0 | 0.092 | 0 | 0.492 | 0.519 |
| **TUBB** | **CDK16** | 1852162 | 1846467 | 9606.ENSP00000339001 | 9606.ENSP00000276052 | 0 | 0 | 0 | 0 | 0 | 0.092 | 0 | 0.492 | 0.519 |
| **PTPN20A** | **DSTYK** | 1856098 | 1854531 | 9606.ENSP00000363459 | 9606.ENSP00000356130 | 0 | 0 | 0 | 0 | 0 | 0.074 | 0 | 0.496 | 0.513 |
| **JUNB** | **ELK1** | 1848518 | 1843855 | 9606.ENSP00000303315 | 9606.ENSP00000247161 | 0 | 0 | 0 | 0 | 0 | 0.204 | 0 | 0.41 | 0.511 |
| **RPL32** | **RAD23B** | 1858438 | 1853623 | 9606.ENSP00000380156 | 9606.ENSP00000350708 | 0 | 0 | 0 | 0 | 0.237 | 0.347 | 0 | 0.091 | 0.508 |
| **MAPK8** | **STAT5A** | 1853988 | 1852428 | 9606.ENSP00000353483 | 9606.ENSP00000341208 | 0 | 0 | 0 | 0 | 0 | 0 | 0 | 0.507 | 0.507 |
| **SERPINB5** | **TPM1** | 1857570 | 1845983 | 9606.ENSP00000372221 | 9606.ENSP00000267996 | 0 | 0 | 0 | 0 | 0 | 0 | 0 | 0.506 | 0.506 |
| **CKB** | **HMGB2** | 1848031 | 1847742 | 9606.ENSP00000299198 | 9606.ENSP00000296503 | 0 | 0 | 0 | 0 | 0 | 0.506 | 0 | 0 | 0.506 |
| **MLANA** | **TP53** | 1857386 | 1846083 | 9606.ENSP00000370880 | 9606.ENSP00000269305 | 0 | 0 | 0 | 0 | 0 | 0 | 0 | 0.506 | 0.506 |
| **MAPK8** | **BIRC5** | 1853988 | 1848314 | 9606.ENSP00000353483 | 9606.ENSP00000301633 | 0 | 0 | 0 | 0 | 0 | 0 | 0 | 0.505 | 0.505 |
| **MECP2** | **EZH2** | 1859873 | 1850211 | 9606.ENSP00000395535 | 9606.ENSP00000320147 | 0 | 0 | 0 | 0 | 0 | 0 | 0 | 0.503 | 0.503 |
| **CHEK2** | **FEN1** | 1857546 | 1848737 | 9606.ENSP00000372023 | 9606.ENSP00000305480 | 0 | 0 | 0 | 0 | 0.09 | 0.165 | 0 | 0.395 | 0.5 |
| **PDPK1** | **TP53** | 1852785 | 1846083 | 9606.ENSP00000344220 | 9606.ENSP00000269305 | 0 | 0 | 0 | 0 | 0 | 0 | 0 | 0.5 | 0.499 |
| **MAPK8** | **MEF2A** | 1853988 | 1853046 | 9606.ENSP00000353483 | 9606.ENSP00000346389 | 0 | 0 | 0 | 0 | 0 | 0 | 0 | 0.498 | 0.498 |
| **CDK16** | **CDKN2C** | 1846467 | 1845196 | 9606.ENSP00000276052 | 9606.ENSP00000262662 | 0 | 0 | 0 | 0 | 0 | 0.149 | 0 | 0.432 | 0.496 |
| **TUBB** | **RAC2** | 1852162 | 1843949 | 9606.ENSP00000339001 | 9606.ENSP00000249071 | 0 | 0 | 0 | 0 | 0 | 0.11 | 0 | 0.454 | 0.493 |
| **CHEK2** | **BIRC5** | 1857546 | 1848314 | 9606.ENSP00000372023 | 9606.ENSP00000301633 | 0 | 0 | 0 | 0 | 0.071 | 0 | 0 | 0.472 | 0.488 |
| **GMEB1** | **CBFA2T3** | 1847483 | 1846034 | 9606.ENSP00000294409 | 9606.ENSP00000268679 | 0 | 0 | 0 | 0 | 0 | 0 | 0 | 0.486 | 0.485 |
| **SLC25A6** | **NR1I2** | 1857377 | 1851892 | 9606.ENSP00000370808 | 9606.ENSP00000336528 | 0 | 0 | 0 | 0 | 0 | 0 | 0 | 0.485 | 0.485 |
| **MEF2A** | **KIT** | 1853046 | 1847112 | 9606.ENSP00000346389 | 9606.ENSP00000288135 | 0 | 0 | 0 | 0 | 0 | 0.064 | 0 | 0.468 | 0.481 |
| **TBX5** | **FOXA3** | 1849239 | 1848592 | 9606.ENSP00000309913 | 9606.ENSP00000304004 | 0 | 0 | 0 | 0 | 0 | 0.149 | 0 | 0.415 | 0.481 |
| **STAT5A** | **BIRC5** | 1852428 | 1848314 | 9606.ENSP00000341208 | 9606.ENSP00000301633 | 0 | 0 | 0 | 0 | 0 | 0 | 0 | 0.48 | 0.48 |
| **PKNOX1** | **CDK2** | 1847291 | 1845906 | 9606.ENSP00000291547 | 9606.ENSP00000266970 | 0 | 0 | 0 | 0 | 0 | 0.45 | 0 | 0.09 | 0.478 |
| **CHEK2** | **CDK2** | 1857546 | 1845906 | 9606.ENSP00000372023 | 9606.ENSP00000266970 | 0 | 0 | 0 | 0.713 | 0.11 | 0.26 | 0 | 0.843 | 0.478 |
| **PBX1** | **MEOX2** | 1860561 | 1845048 | 9606.ENSP00000405890 | 9606.ENSP00000262041 | 0 | 0 | 0 | 0 | 0 | 0.149 | 0 | 0.409 | 0.475 |
| **TP53** | **XBP1** | 1846083 | 1842534 | 9606.ENSP00000269305 | 9606.ENSP00000216037 | 0 | 0 | 0 | 0 | 0 | 0 | 0 | 0.472 | 0.472 |
| **HSPA1A** | **TUBB** | 1856351 | 1852162 | 9606.ENSP00000364802 | 9606.ENSP00000339001 | 0 | 0 | 0 | 0 | 0 | 0.101 | 0 | 0.435 | 0.471 |
| **CDK18** | **STUB1** | 1861603 | 1842759 | 9606.ENSP00000423665 | 9606.ENSP00000219548 | 0 | 0 | 0 | 0 | 0 | 0.471 | 0 | 0 | 0.471 |
| **MSN** | **CDK2** | 1853976 | 1845906 | 9606.ENSP00000353408 | 9606.ENSP00000266970 | 0 | 0 | 0 | 0 | 0 | 0.415 | 0 | 0.125 | 0.466 |
| **CDC25A** | **BIRC5** | 1848560 | 1848314 | 9606.ENSP00000303706 | 9606.ENSP00000301633 | 0 | 0 | 0 | 0 | 0.094 | 0 | 0 | 0.434 | 0.465 |
| **FEN1** | **HMGB2** | 1848737 | 1847742 | 9606.ENSP00000305480 | 9606.ENSP00000296503 | 0 | 0 | 0 | 0 | 0.081 | 0.11 | 0 | 0.396 | 0.462 |
| **PDPK1** | **PRKCH** | 1852785 | 1851111 | 9606.ENSP00000344220 | 9606.ENSP00000329127 | 0 | 0 | 0 | 0.738 | 0 | 0.36 | 0 | 0.618 | 0.46 |
| **IRF4** | **STAT5A** | 1857309 | 1852428 | 9606.ENSP00000370343 | 9606.ENSP00000341208 | 0 | 0 | 0 | 0 | 0 | 0 | 0 | 0.458 | 0.457 |
| **TRAF2** | **XBP1** | 1843873 | 1842534 | 9606.ENSP00000247668 | 9606.ENSP00000216037 | 0 | 0 | 0 | 0 | 0 | 0 | 0 | 0.456 | 0.456 |
| **BAG3** | **RAD23B** | 1854947 | 1853623 | 9606.ENSP00000358081 | 9606.ENSP00000350708 | 0 | 0 | 0 | 0 | 0 | 0.149 | 0 | 0.388 | 0.456 |
| **HBG1** | **SMARCE1** | 1850918 | 1850602 | 9606.ENSP00000327431 | 9606.ENSP00000323967 | 0 | 0 | 0 | 0 | 0 | 0 | 0 | 0.454 | 0.454 |
| **STAT5A** | **JUNB** | 1852428 | 1848518 | 9606.ENSP00000341208 | 9606.ENSP00000303315 | 0 | 0 | 0 | 0 | 0 | 0.154 | 0 | 0.378 | 0.451 |
| **NFYA** | **CCNB1** | 1852963 | 1844435 | 9606.ENSP00000345702 | 9606.ENSP00000256442 | 0 | 0 | 0 | 0 | 0.065 | 0.073 | 0 | 0.414 | 0.447 |
| **MAPK8** | **CCNB1** | 1853988 | 1844435 | 9606.ENSP00000353483 | 9606.ENSP00000256442 | 0 | 0 | 0 | 0 | 0 | 0 | 0 | 0.446 | 0.446 |
| **FOXA3** | **TBX6** | 1848592 | 1846629 | 9606.ENSP00000304004 | 9606.ENSP00000279386 | 0 | 0 | 0 | 0 | 0 | 0.149 | 0 | 0.376 | 0.446 |
| **CHEK2** | **CDK16** | 1857546 | 1846467 | 9606.ENSP00000372023 | 9606.ENSP00000276052 | 0 | 0 | 0 | 0.625 | 0 | 0.26 | 0 | 0.686 | 0.446 |
| **JUNB** | **CCND1** | 1848518 | 1843117 | 9606.ENSP00000303315 | 9606.ENSP00000227507 | 0 | 0 | 0 | 0 | 0 | 0 | 0 | 0.443 | 0.443 |
| **HMGB2** | **CDK2** | 1847742 | 1845906 | 9606.ENSP00000296503 | 9606.ENSP00000266970 | 0 | 0 | 0 | 0 | 0.112 | 0.309 | 0 | 0.163 | 0.442 |
| **CDK2** | **ASB1** | 1845906 | 1845574 | 9606.ENSP00000266970 | 9606.ENSP00000264607 | 0 | 0 | 0 | 0 | 0 | 0.149 | 0 | 0.37 | 0.44 |
| **CDK18** | **CDKN2C** | 1861603 | 1845196 | 9606.ENSP00000423665 | 9606.ENSP00000262662 | 0 | 0 | 0 | 0 | 0 | 0.149 | 0 | 0.37 | 0.44 |
| **CDK18** | **ASB1** | 1861603 | 1845574 | 9606.ENSP00000423665 | 9606.ENSP00000264607 | 0 | 0 | 0 | 0 | 0 | 0.149 | 0 | 0.37 | 0.44 |
| **CDK16** | **ASB1** | 1846467 | 1845574 | 9606.ENSP00000276052 | 9606.ENSP00000264607 | 0 | 0 | 0 | 0 | 0 | 0.149 | 0 | 0.37 | 0.44 |
| **RPL32** | **CDK2** | 1858438 | 1845906 | 9606.ENSP00000380156 | 9606.ENSP00000266970 | 0 | 0 | 0 | 0 | 0 | 0.309 | 0 | 0.214 | 0.434 |
| **TPM1** | **STUB1** | 1845983 | 1842759 | 9606.ENSP00000267996 | 9606.ENSP00000219548 | 0 | 0 | 0 | 0 | 0 | 0.407 | 0 | 0.08 | 0.431 |
| **CDC25A** | **CDKN2C** | 1848560 | 1845196 | 9606.ENSP00000303706 | 9606.ENSP00000262662 | 0 | 0 | 0 | 0 | 0 | 0 | 0 | 0.431 | 0.431 |
| **STK38L** | **CCNB1** | 1857786 | 1844435 | 9606.ENSP00000373684 | 9606.ENSP00000256442 | 0 | 0 | 0 | 0 | 0 | 0.361 | 0 | 0.146 | 0.43 |
| **FOXA3** | **HNF1B** | 1848592 | 1843059 | 9606.ENSP00000304004 | 9606.ENSP00000225893 | 0 | 0 | 0 | 0 | 0.106 | 0 | 0 | 0.387 | 0.429 |
| **CASP7** | **STAT4** | 1855004 | 1853690 | 9606.ENSP00000358327 | 9606.ENSP00000351255 | 0 | 0 | 0 | 0 | 0 | 0 | 0 | 0.427 | 0.427 |
| **PBX1** | **TGIF1** | 1860561 | 1850975 | 9606.ENSP00000405890 | 9606.ENSP00000327959 | 0 | 0 | 0 | 0.626 | 0 | 0.3 | 0 | 0.495 | 0.424 |
| **FEN1** | **CDK16** | 1848737 | 1846467 | 9606.ENSP00000305480 | 9606.ENSP00000276052 | 0 | 0 | 0 | 0 | 0.09 | 0.246 | 0 | 0.226 | 0.422 |
| **EZR** | **TP53** | 1852156 | 1846083 | 9606.ENSP00000338934 | 9606.ENSP00000269305 | 0 | 0 | 0 | 0 | 0 | 0 | 0 | 0.423 | 0.422 |
| **TP53** | **BIRC7** | 1846083 | 1842643 | 9606.ENSP00000269305 | 9606.ENSP00000217169 | 0 | 0 | 0 | 0 | 0 | 0 | 0 | 0.42 | 0.42 |
| **FEN1** | **CCNB1** | 1848737 | 1844435 | 9606.ENSP00000305480 | 9606.ENSP00000256442 | 0 | 0 | 0 | 0 | 0.16 | 0.113 | 0 | 0.283 | 0.419 |
| **PDPK1** | **CCND1** | 1852785 | 1843117 | 9606.ENSP00000344220 | 9606.ENSP00000227507 | 0 | 0 | 0 | 0 | 0 | 0 | 0 | 0.419 | 0.418 |
| **MUTYH** | **TP53** | 1855617 | 1846083 | 9606.ENSP00000361170 | 9606.ENSP00000269305 | 0 | 0 | 0 | 0 | 0 | 0 | 0 | 0.418 | 0.418 |
| **JUNB** | **TP53** | 1848518 | 1846083 | 9606.ENSP00000303315 | 9606.ENSP00000269305 | 0 | 0 | 0 | 0 | 0 | 0 | 0 | 0.419 | 0.418 |
| **TP53** | **TPM1** | 1846083 | 1845983 | 9606.ENSP00000269305 | 9606.ENSP00000267996 | 0 | 0 | 0 | 0 | 0 | 0.173 | 0 | 0.323 | 0.416 |
| **BIRC5** | **KIT** | 1848314 | 1847112 | 9606.ENSP00000301633 | 9606.ENSP00000288135 | 0 | 0 | 0 | 0 | 0 | 0 | 0 | 0.416 | 0.416 |
| **CHEK2** | **TUBB** | 1857546 | 1852162 | 9606.ENSP00000372023 | 9606.ENSP00000339001 | 0 | 0 | 0 | 0 | 0 | 0 | 0 | 0.41 | 0.411 |
| **MSN** | **RAC2** | 1853976 | 1843949 | 9606.ENSP00000353408 | 9606.ENSP00000249071 | 0 | 0 | 0 | 0 | 0 | 0.094 | 0 | 0.375 | 0.409 |
| **FEN1** | **TP53** | 1848737 | 1846083 | 9606.ENSP00000305480 | 9606.ENSP00000269305 | 0 | 0 | 0 | 0 | 0 | 0 | 0 | 0.408 | 0.408 |
| **CHEK2** | **PPP2CB** | 1857546 | 1842815 | 9606.ENSP00000372023 | 9606.ENSP00000221138 | 0 | 0 | 0 | 0 | 0 | 0.301 | 0 | 0.189 | 0.408 |
| **KIT** | **RAC2** | 1847112 | 1843949 | 9606.ENSP00000288135 | 9606.ENSP00000249071 | 0 | 0 | 0 | 0 | 0 | 0.113 | 0 | 0.36 | 0.408 |
| **JUNB** | **PPP2CB** | 1848518 | 1842815 | 9606.ENSP00000303315 | 9606.ENSP00000221138 | 0 | 0 | 0 | 0 | 0 | 0 | 0 | 0.407 | 0.407 |
| **STAT4** | **HNF1B** | 1853690 | 1843059 | 9606.ENSP00000351255 | 9606.ENSP00000225893 | 0 | 0 | 0 | 0 | 0 | 0 | 0 | 0.407 | 0.407 |
| **HSPA1A** | **MAPK8** | 1856351 | 1853988 | 9606.ENSP00000364802 | 9606.ENSP00000353483 | 0 | 0 | 0 | 0 | 0 | 0 | 0 | 0.404 | 0.404 |
